# Computational modelling of the regulation of Insulin signalling by oxidative stress

**DOI:** 10.1186/1752-0509-7-41

**Published:** 2013-05-24

**Authors:** Graham R Smith, Daryl P Shanley

**Affiliations:** 1 Centre for Integrated Systems Biology of Ageing & Nutrition (CISBAN), Institute for Ageing and Health, Newcastle University, Campus for Ageing and Vitality, Newcastle upon Tyne NE4 5PL, UK

**Keywords:** Insulin signalling, FOXO, Oxidative stress, Kinetic computational modelling

## Abstract

**Background:**

Existing models of insulin signalling focus on short term dynamics, rather than the longer term dynamics necessary to understand many physiologically relevant behaviours. We have developed a model of insulin signalling in rodent adipocytes that includes both transcriptional feedback through the Forkhead box type O (FOXO) transcription factor, and interaction with oxidative stress, in addition to the core pathway. In the model Reactive Oxygen Species are both generated endogenously and can be applied externally. They regulate signalling though inhibition of phosphatases and induction of the activity of Stress Activated Protein Kinases, which themselves modulate feedbacks to insulin signalling and FOXO.

**Results:**

Insulin and oxidative stress combined produce a lower degree of activation of insulin signalling than insulin alone. Fasting (nutrient withdrawal) and weak oxidative stress upregulate antioxidant defences while stronger oxidative stress leads to a short term activation of insulin signalling but if prolonged can have other effects including degradation of the insulin receptor substrate (IRS1) and FOXO. At high insulin the protective effect of moderate oxidative stress may disappear.

**Conclusion:**

Our model is consistent with a wide range of experimental data, some of which is difficult to explain. Oxidative stress can have effects that are both up- and down-regulatory on insulin signalling. Our model therefore shows the complexity of the interaction between the two pathways and highlights the need for such integrated computational models to give insight into the dysregulation of insulin signalling along with more data at the individual level.

A complete SBML model file can be downloaded from BIOMODELS (https://www.ebi.ac.uk/biomodels-main) with unique identifier MODEL1212210000.

Other files and scripts are available as additional files with this journal article and can be downloaded from https://github.com/graham1034/Smith2012_insulin_signalling.

## Background

Nutrient response signalling pathways are activated in response to feeding and control such aspects of an organism’s response to feeding as satiety and the generation, consumption and storage of energy. They also control the allocation of energy and biological substrates to somatic growth, maintenance and repair, and so implicitly underlie theories of ageing such as the Disposable Soma Theory [1] which are based on the idea of trade-offs between these processes. In the animal kingdom the insulin-signalling (IS) pathway is of particular importance [2]. Dietary sugars trigger the production of insulin or insulin-like peptides which are sensed by Insulin receptors at the cell surface and initiate a kinase cascade, leading to the activation of the downstream kinase Akt (PKB), the translocation of GLUT4 glucose transporters to the cell surface, and ultimately glucose uptake. Longer-term adaptive effects are controlled through transcription factors such as FOXO, which is deactivated (via cytoplasmic translocation) by Akt-mediated phosphorylation when IS is active [3]. When active, FOXO transcribes genes regulating cell cycle arrest, apoptosis, metabolism and maintenance [4,5]. Animal lifespan may be increased by nutrient restriction in many animals including nematodes, flies and mice [6], at least in part through the decrease in activity of IS [2,7,8]. In addition to its role in ageing, dysregulation of IS is central in metabolic diseases such as Type II Diabetes (T2D).

Oxidative stress is an inescapable concomitant of the life of aerobic organisms, given that reactive oxygen species (ROS) are produced by energy generation by oxidative phosphorylation in mitochondria. ROS can damage all main categories of biomolecule including DNA, protein and lipids. Accordingly, organisms protect themselves by the production of many classes of antioxidant enzymes, including superoxide dismutase and catalase (that catalyse reactions that lead ultimately to the conversion of ROS to water), and thioredoxins and peroxiredoxins (that use reversible oxidation of sulphydryl groups to remove ROS) [9]. The extent of cellular resistance to oxidative stress varies between organisms and closely correlates with lifespan [10], and in the free radical theory of ageing [11] the damage caused by ROS is hypothesised to be the primary cause of age-related degeneration [12]. Notwithstanding this, recent studies have tended to emphasise that the levels of antioxidant enzymes do not correlate with lifespan [13], and the effects of overexpression or knockout of antioxidants, or of dietary supplementation, are not always as might be expected [14,15]. In addition ROS activate several stress-activated protein kinases (SAPKs) such as JNK, IKK [16] and p38 MAPK [17]. The response of IS to oxidative stress is complicated by the fact that ROS has been co-opted into the active regulation of the pathway, ROS produced by NOX enzymes affecting IS immediately through the reversible oxidation and inhibition of the catalytic cysteine residues of the protein tyrosine phosphatases and lipid phosphatases that deactivate IS [18-21]. The effect of ROS on IS has also been strongly implicated in the development of metabolic disease such as T2D [22-24] and neurodegenerative disease [25].

Given the complicated nature of these two important processes and their interaction, especially the multiple countervailing effects of ROS, a quantitative model is essential to understand the biological consequences. In particular, the ability of a computational model to quantify multiple effects and study the effects of variation of experimentally-inaccessible parameters may be expected to lead to some insight. Motivated by this, we here present a kinetic computational model of IS in rodent adipocytes including FOXO and some FOXO-mediated outputs, and including interactions with ROS and some oxidative stress activated kinases. Previous modelling work on IS has focused on receptor binding, the activation of Akt and Glucose uptake [26,27]. In recent years it has expanded to include a more detailed description of Insulin-InR binding [28], integration with connected signalling pathways such as mTOR [29,30], EGF [31], MAPK [32] and whole-body insulin/glucose dynamics [33], though these aspects are not included in the model presented here. We have used work by Sedaghat et al. [26] as the basis of our modelling of IS and integrated it with a model we previously developed of the regulation of the FOXO transcription factor by multiple post-translational modifications (PTMS) [34]. The role of stress-activated kinases JNK and IKK in regulating FOXO was already included in this model; here we have extended this to include their regulation of IS through phosphorylation of IRS. ROS is included in the model and leads to activation of SAPKs while also having a direct effect on IS through the phosphatase-dependent mechanism described above. In the model, insulin stimulation leads to signalling-induced ROS through NOX but not additional mitochondrially generated ROS resulting from glucose uptake (this would be an oversimplification in other tissues such as muscle, where mitochondrially generated ROS makes up a larger fraction of the total). Aspects of a recent model of the antioxidant system [35] describing the transport of oxidants between the extracellular and cytoplasmic compartment were included; this enables connection with experiments in which oxidants (hydrogen peroxide) are externally applied. We develop a model integrating multiple experimental findings on the IS and oxidative stress systems and study its behaviour in scenarios of nutrient withdrawal, the simultaneous applications of Insulin and ROS, and the effects of low and high oxidative stress on insulin response.

## Results

**Figure 1:**
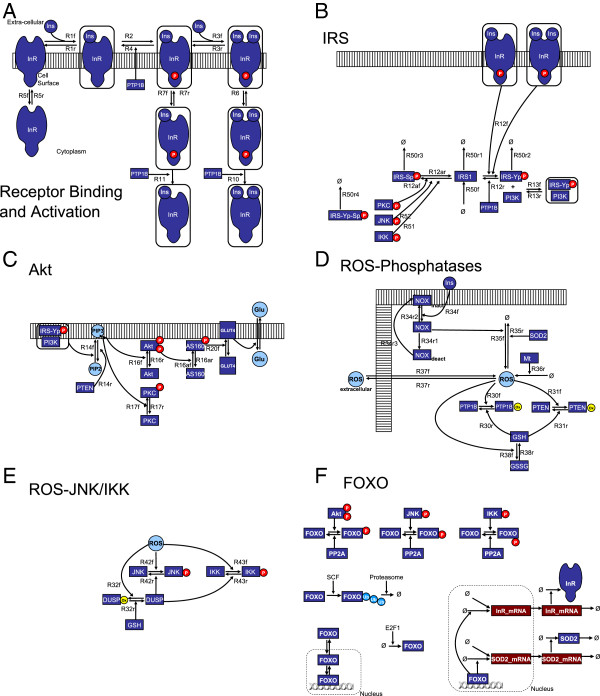
**Block diagrams of the model.** (**A**): Insulin – Insulin Receptor binding and InR activation; (**B**): IRS regulation by active InR, PKC, JNK and IKK and IRS – PI3K binding; (**C**) downstream activation of Akt though the IRS– PI3K complex and PIP3, and GLUT4 transport to the plasma membrane; (**D**) ROS production basally (by Mt) and insulin-stimulated (by NOX); ROS transport across the plasma membrane; ROS degradation by SOD2; inhibition of PTP and PTEN by ROS and reactivation by GSH; (**E**) activation of JNK and IKK by ROS; (**F**) FOXO regulation by Akt, JNK and IKK; FOXO synthesis and degradation; FOXO transport between compartments; transcriptional regulation of InR and SOD2 by FOXO. The presence of the PTMs applied to FOXO by the kinases affects the transport and degradation rates, as detailed in Table 3.

**Figure 2:**
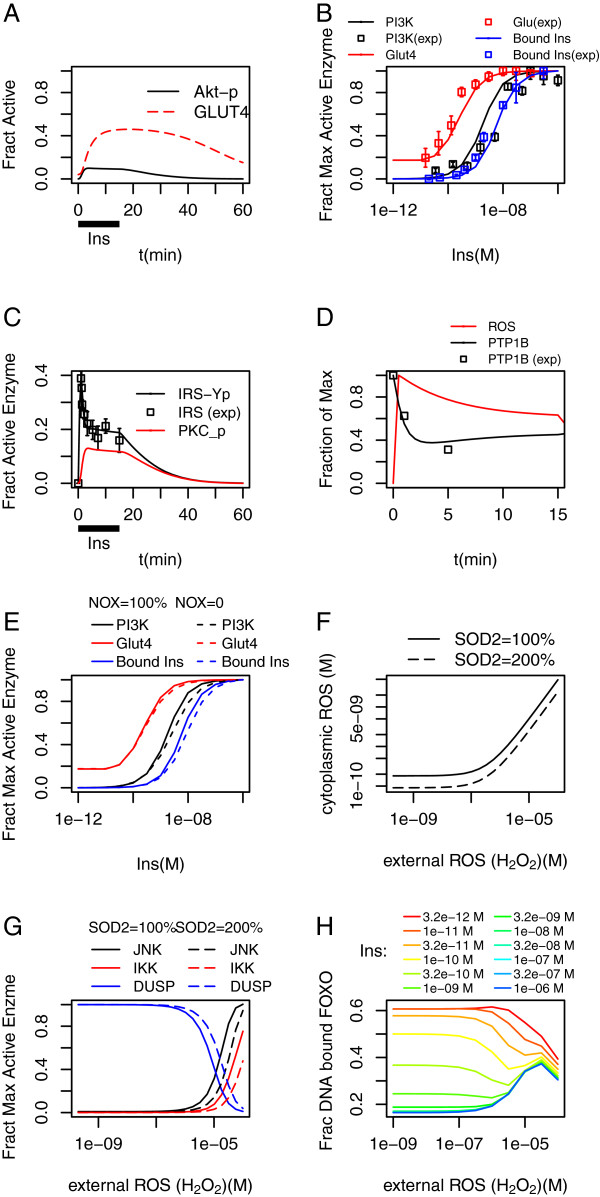
**Short-term effects of insulin signalling and Oxidative stress.** (**A**) Insulin (100 nM; dosing bar) activates Akt leading to Glut4 translocation and Glucose uptake (timecourses). Akt timecourse reproduces Sedaghat et al. [26]; (**B**) Dose response curves for bound insulin, PI3K activation and Glucose uptake; data adapted from experiments by Stagstad et al. [36] in rat adipocytes; (**C**) Insulin activates IRS through Tyrosine phosphorylation (IRS_Yp) and PKC inhibits through serine phosphorylation. (data adapted from experiments by Cedersund et al. [27] in human adipocytes); (**D**) Endogenous ROS inhibits PTP1B (data adapted from experiments by Mahadev et al. [18] in mouse 3T3-L1 adiopocytes); (**E**) Inhibition of NOX prevents oxidation-mediated inactivation of PTP1B and moves D-R curves of IS components to higher insulin concentrations; (**F**) Antioxidants control internal ROS until external ROS passes a threshold (calculations based on a model developed by Adimora et al. [35] in human Jurkat T cells); (**G**) ROS activates JNK/IKK; (**H**) Insulin (through Akt) and ROS (through JNK) modulate FOXO subcellular localization.

The modules making up the model are shown diagrammatically in Figure 1, with outputs shown in Figure 2. Molecule numbers are given in Table 1 and kinetic parameters in Tables 2, 3 and 4. Differential equations are given in Additional file 1. Mass action kinetics are used for the majority of equations, except activation of Akt and PKC and JNK/IKK mediated feedbacks on IRS. The modules in Figure 1A-C make up the core insulin signalling pathway and Figure 2A-C show its behaviour in response to insulin. ROS production and SAPK activation modules are described in Figure 1D-E and their behaviour is shown in Figure 2D-G. The FOXO module is shown in Figure 1F and its behaviour in Figure 2H.

**Table 1:** Particle numbers in initial conditions

**Module**	**Species**	**Initial value (number of molecules)**	**Comments & References**
Receptor binding	Ins	5 × 10^5^, boundary	10^-7^ M
	InR	9 × 10^4^	BioNumbers [37]
	Cytoplasm_InR	1 × 10^4^	<< cell surface
	PTP1B	1 × 10^5^	assigned (PaxDb suggests PTPN1 5 × 10^5^ in human, 5 × 10^4^ in mouse) http://pax-db.org &[38,39]
IRS	IRS1	1 × 10^5^	[26] Appdx B
	PI3K	1 × 10^4^	[26] Appdx B
	PP2A	5 × 10^5^	[40]
Phosphatases	PTEN	1 × 10^5^	PaxDb, human
	GSH	100	assigned
	NOX_inact	100	assigned
	Cytoplasm_SOD2	4.17 × 10^4^	scale 10^-3^ * PaxDb
	Mt	50	assigned
	extracellular_ROS	5 × 10^4^, boundary	scale 10^-3^ * 10 μM
Akt	Akt	1 × 10^5^	[41]
	AS160	2 × 10^4^	assigned
	PKC	1 × 10^5^	assigned same as Akt
	PIP2	2 × 10^5^	scale 10^-3^ *[26]; [42]
	PIP3	1 × 10^3^	scale 10^-3^ * [26]; [42]
	cytoplasm_GLUT4	9.6 × 10^4^	Total 1 × 10^5^, assigned ((PaxDb suggests Slc2a4 5 × 10^4^ in mouse)
	cellsurface_GLUT4	4 × 10^3^	
JNK	JNK	16000	PaxDB (Mapk9)
	IKK	2000	PaxDb (Ikbkb)
	DUSP	1 × 10^5^	assigned
FOXO	E2F1	300	assigned, to keep FOXO ~ 1000
	cytoplasm_Foxo1_Pa0 Pd0_Pe0_pUb0 (Foxo with no PTMs)	1000	see [34]

All other species are initially zero.

Initial particle numbers in the model (all other species are initially zero).

**Table 2:** Parameter values of the model (except for FOXO part)

**Module**	**Reaction**	**Name**	**Parameter**	**Value**	**Order**	**Comments & Ref**
All		Volume	extracellular	8.3 × 10^-12^ l		0.5 × cytoplasm
		Volume	cell surface	6.4 × 10^-14^ l		assumes thickness of 20 nm
		Volume	cytoplasm	1.65 × 10^-11^ l		typical of many cells
		Volume	nucleus	5 × 10^-13^ l		
		Volume	DNA-bound	1 × 10^-13^ l		
Receptor binding	R1f		k1	2.0 × 10^-5^	2	[36] Figure 1
m8b2_recep.6mod	R1r		kminus1	12072	1	[36]
	R2		k3	2500	1	[36]
	R3f		k2	1.2 × 10^-5^	2	[36]
	R3r		kminus2	9 × 10^4^	1	> > kminus1
	R4		kminus3	2 × 10^-6^	2 (cat)	
	R5f		k4	0.0333	1	forward/reverse ratio 10, much faster than [26]
	R5r		kminus4	0.3	1	see R5f
	R6f		k4’	0.0021	1	unchanged from [26]
	R6r		kminus4’	2.1 × 10^-4^	1	[26]
	R7f		k4’	0.0021	1	[26]
	R7r		kminus4’	2.1 × 10^-4^	1	[26]
	R10		k6	4.61 × 10^-6^	2 (cat)	[26] corrected for mol number of PTP1B
	R11		k6	4.61 × 10^-6^	2 (cat)	see R10
IRS	R12f		k7	5.8	2 (cat)	fitted to IRSYp
m8b2_irs.6.mod	R12r		kminus7a	8.75 × 10^-5^	2 (cat)	as R12f
	R12_a_f	IRS-SerP by PKC_P	kpsp2	2.2 × 10^-4^	2	changed to simple MA; [27] used for fitting
	R12_a_r		kminus7b	0.28 × 10^-5^	2 (cat)	as R12_a_f
	R12_b_(f,r)		as R12_a_(f,r)			
	R13f		k8	2.6 × 10^-6^	2	[36] Figure 3
	R13r		kminus8	1.55	1	as R13f
	R50f	IRS basal synth	k_irs1_basal_syn	260	0	increased to keep IRS1 const
	R50r1	IRS degrade	k_irs_basal_degr	10^-3^	1	Lifetime of ~2 days
	R50r2	IRS-Yp degr	k_irs_basal_degr	10^-3^	1	as R50r2
	R50r3	IRS1-Sp degr	k_irs_polyserp_degr	10^-2^	1	10 × faster than r50r2 [43]
	R51	IRS-SerP by IKK_P	kcat51	0.87		consistent with [43] and IKK/JNK activation
		IRS-SerP by IKK_P	Km51	100		as above
	R52	IRS-SerP by JNK_P	kcat52	6.95		as R51
		IRS-SerP by JNK_P	Km52	100		as above
Phosphatases	R30f	oxidation of PTP1B	k30f	0.08	2	[18,19]
m8b2_phosph.6.mod	R30r	reduction of PTP1B by GSH	k30r	5 × 10^-3^	2	[18-20]
	R31f	oxidation of PTEN	k31f	2.7 × 10^-4^	2	slower than R30f
	R31r	reduction of PTEN by GSH	k31r	2 × 10^-3^	2	similar to R30r
	R34f	Activation of NOX	k34f	2 × 10^-5^	2	consistent with R35
	R34r1	NOX deactivation	k34r1	10^-3^	2	consistent with R35
	R34r2	NOX inactivation(1)	k34r2	0.25	1	consistent with R35
	R34r3	NOX deact- > inact	k34r3	10^-3^	2	consistent with R35
	R35f	ROS production by NOX	k35f	450	1	fitted to get desired ROS level (30/5) and PTP1B inhibition [18]
	R35r	ROS elimination by SOD2	k35r	0.12	2	lifetime < 0.01 s
	R36f	ROS production by Mitoch	k36f	180	1	
	R37f, R37r	ROS transport across plasma membrane	ros_perm	7.8 × 10^8^ (thousands cm^-2^min^-1^)	1	[35]
			membrane_area	6.5 × 10^-9^ cm^2^		from volume
			k_ros_perm	4.8 thousands min^-1^		product of above
	R38f	Glutathione oxidation	k38f	0.05	2	see R38f
	R38r	Glutathione reduction	k38r	2	1	(auto-reduction)
Akt	R14f	PI(3,4,5)P3 generation, basal	k9_basal	0.13145	1	[26]
m8b2_akt.6.mod		By PI3K complex	k9	0.055	2 (cat)	derived from [26]
	R14r	PIP3 breakdown, basal	kminus9_basal	2.7	1	[26]
		by PTEN	kminus9	0.0014	2 (cat)	
	R16f	Akt activation	k11	2.5 × 10^-5^	2	modified mass action, effect similar to express in [26]
			pip3_basal	200	2 (cat)	offset
	R16r	Akt deactivation by PP2A	kminus11	1.188 × 10^-6^	2 (cat)	[26]
	R17f	PKC activation	k12	3.5 × 10^-5^	2 (cat)	as R16f
	R17r	PKC deactivation	kminus12	1.25 × 10^-6^	2 (cat)	as R16r
	R16a	AS160 activation by AKT	kr16a	3.33 × 10^-4^	2 (cat)	see methods; [26,36]
	R16r	AS160 deactivation	kminusr16a	1 × 10^-6^	2 (cat)	see R16a
	R20f	GLUT4 translocation to cell surface; basal	k13_basal	0.015	1	see R16a
		and by AS160	k13	7.5 × 10^-6^	2 (cat)	see R16a
	R20r	GLUT4 translocation to cytoplasm	kminus13	0.167	1	see R16a
JNK	R32f	DUSP oxidation	k32f	6 × 10^-4^	2 (cat)	consistent with [16,35,44]
m8b2_jnk.6.mod	R32r	DUSP reduction by GSH	k32r	4 × 10^-4^	2 (cat)	as R32f
	R42f	JNK activation by ROS	k42f^a^	2.5 × 10^-4^	2 (cat)	as R32f
	R42r	JNK deactivation by DUSP	k42r	0.5 × 10^-6^	2 (cat)	as R32f
	R43f	IKK activation by ROS	k43f	0.5 × 10^-4^	2 (cat)	as R32f
	R43r	IKK deactivation	k43r	0.5 × 10^-6^	2 (cat)	as R32f

^a^ k42f expressed as α × k42f’ where α = 5 and k42f’ = 0.5 × 10^-4^.

(phosphatases, Akt, JNK, FOXO).

Kinetic parameters of the model, except for the FOXO components. Units of rate constants are min-1 for first order or (number)-1 min-1 for second order.

**Table 3:** FOXO parameters for synthesis, degradation, transport and PTMs

**Process**	**2**^**o **^**rate constant (#min**^**-1**^**)**	**Enzyme**	**Enz #**	**1**^**o **^**rate const (min**^**-1**^**)**	**PTM modifying rate**	**Rate MF**	**Reference**
Synthesis (NULL → FOXO)	0.0055	E2F1	300	5.5			[45] Figure 1
Cytoplasm → nuclear transport	-	-	-	0.1/0.55 = 0.182			[3], [46]
					Akt-Phos Pa	0.1	[46]
					IKK-Phos Pd	0.5	[47] Figure 1
					JNK-Phos Pe	10	[44]
Nuclear → cytoplasm transport	-	-	-	0.1 × 0.55 = 0.055			[3], [46]
					Akt-Phos Pa	10	[46]
					IKK-phos Pd	10	[47]
					JNK-Phos Pe	0.1	[44]
Nuclear → DNAbound transport	-	-	-	0.25			[48]
					Akt-Phos Pa	0.5	[49] Figure 6B
DNAbound → Nuclear transport	-	-	-	0.25 × 0.5 = 0.125			[48]
basal phosphorylation	5 × 10^-5^						
basal dephosphorylation	1 × 10^-6^						
akt_phos_ factor	6						
ikk phos_factor	3						
jnk phos_factor	2						
FOXO → FOXO_Pa	akt phos_factor × basal phosphorylation	Akt	(10^4^)	0.5			[50] Figure 1C
FOXO_Pa → FOXO	basal dephosphorylation	PP2A	5 × 10^5^	0.5			
FOXO → FOXO_Pd	ikk phos_factor × basal phosphorylation	IKK	(10^4^)	0.5			[47]
FOXO_Pd → FOXO	basal dephosphorylation	PP2A	5 × 10^5^	0.5			
FOXO → FOXO_Pe	jnk phos_factor × basal phosphorylation	JNK	(10^4^)	0.5			[44]
FOXO_Pe → FOXO	basal dephosphorylation	PP2A	5 × 10^5^	0.5			
FOXO → FOXO_pUb	1 × 10^-6^	SCF/MDM2	10^3^	10^-3^			[45] Figure 1; also [51]
					Akt (Pa)	3	[45] Figure 1
					IKK (Pd)	22	[47] Figure 5F
Degradation (FOXO_pUb → NULL)	1 × 10^-4^	Proteasome	10^3^	0.1			[45] Figure 1

Kinetic parameters of the FOXO module of the model. Where a second-order rate constant is given and the Enzyme is time-varying, the Enz # and first order rate constant columns show typical values when the process is activated. The PTM modifying rate refers to the way modifiable species are implemented in this system: if the PTM is present, then the basal rate for the process (in the 1o rate column) is multiplied by the entry in the Rate MF (Multiplicative Factor) column.

**Table 4:** Parameters for FOXO mediated transcription

**Protein**	**FOXO- simulated transcription k**_**transl**_	**Basal transcription k**_**basalt**_	**RNA export k**_**exp**_	**RNA degradation k**_**mdeg**_	**RNA translation k**_**transl**_	**Protein degradation k**_**pdeg**_	**Ref**
InR	0.24	5.0	0.22	5.622	2.46	4.4× 10^-3^	[52,53]
SOD2	0.95	15.0	0.22	5.622	1.23	1.9 × 10^-3^	[52][44,54]

Parameters for transcription, transport, translation and degradation of the FOXO-regulated InR and SOD2 genes and proteins.

### Model development and parameter estimation

#### Insulin signalling

**Figure 3:**
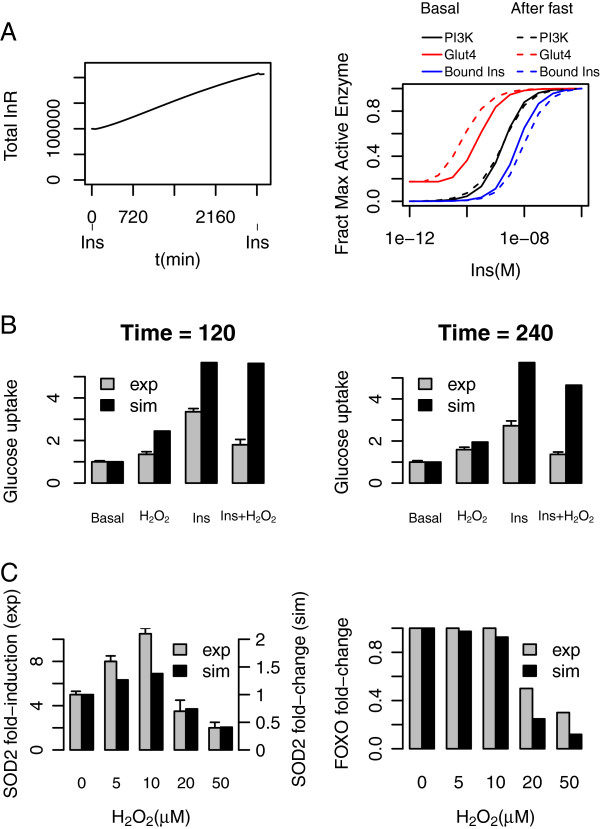
**Long term effects and effects of inter-subsystem interactions (A) Fasting; Insulin receptors are upregulated during a fast (left panel).** Insulin is applied for 15 minutes at the beginning and end of the two-day period (dosing bars at t = 0 and t = 2785 in left panel) producing the effect on the Insulin dose–response curve at the end of the fasting period shown in the right panel; (**B**) Interaction of Insulin Stimulation and Oxidative stress; Insulin activates glucose uptake strongly; oxidative stress activates it weakly, but reduces the effect of insulin. Data adapted from experiments by Archuleta et al. [17] in rat skeletal muscle; (**C**) Low oxidative stress increase antioxidant defence, high oxidative stress decreases it. Data adapted from experiments by Essers et al. [44] in human DLD1 human colon carcinoma cells. Both simulation and experiment at t = 960 min (16 h) after exposure.

The parameters in the insulin signalling pathway itself were arrived at as follows. Model development was started with the model in Sedaghat et al. [26] as a basis, but was substantially refitted in the light of the timecourse of PTP1B inhibition and extra data. Various sources were used to obtain particle numbers, particularly PaxDb [38,39]. In the receptor binding module, insulin binding and unbinding (k1 and kminus1) were fitted using data on bound insulin in rat adipocytes from Stagsted et al. Figure 1 [36]. In the IRS module k7 and kminus7 were fitted to IRS_tyrosine phosphorylation data in human adipocytes from Cedersund et al. [27] to get the rapid rise. k8 and k-8, the rate constants for Reaction 13, the formation of the IRS-PI3K complex, were fitted to PI3K activation kinetics from Stagsted et al. Figure 3 [36], with the additional information that max PI3K = 0.05 of total [26]. Parameters of PIP3 production and degradation were fitted to give PIP3 activation on a timescale of 30s, rising from 0.003 (basal) to 0.03 (maximum stimulated) [26]. Parameters of Akt and PKC activation (reactions R16 and R17) were chosen to give maximum insulin-stimulated Akt and PKC phosphorylation = 10% of total after 2 minutes. The exact time is uncertain given the data used here but must be rapid to enable parameters of IRS – serine phosphorylation (in R12af) to produce the observed feedback inhibition of IRS1. In the rate law, an offset to the mass-action dependence on PIP3 was used to give zero Akt/PKC activation below the basal PIP3 level (parameter pip3_basal; see Additional file 1). Glucose uptake is proxied by cell surface GLUT4, and so GLUT4 parameters were chosen so that a fraction 0.1 of GLUT4 is located at the cell surface basally, and 0.6 at maximum. The fact that GLUT4 is not entirely surface located at its maximum, but this maximum must occur at a lower Insulin concentration than the peak activation of PI3K (and Akt) activation, necessitates the addition of AS160 [55-57] between Akt and GLUT4 (otherwise, the further activation of PI3K produces more GLUT4 translocation). AS160 is fully activated by partial activation of Akt, at an Insulin concentration of around 1 nM, but even when fully activated leads to only a partial translocation of GLUT4.

#### ROS production and signalling

Insulin stimulation is associated with the production of ROS (hydrogen peroxide) intracellularly [18] and it has been shown in experiments on mouse 3T3-L1 adipocytes that this involves the activation of NOX in a way independent of downstream components of IS that are downstream of IRS [18] Accordingly we include NOX activated directly by Insulin, the NOX passing spontaneously into a deactivated state from which it only slowly returns to the inactive state (Additional file 2: Figure S1A). The NOX-generated ROS is about fivefold above the background level of Mitochondrially-generated ROS (qualitative data from Mahadev et al. [19], (Additional file 2: Figure S1A)) and decays in about 10 min.

It is difficult to quantify the difference between the background and Ins-stimulated ROS production in Mahadev et al.; it has been taken that the NOX generated ROS is about 5× the background level of Mt-generated ROS, and that even the NOX-generated ROS is low enough that JNK activation by insulin is very low, enough to maintain Akt-control of FOXO localization under insulin stimulation. The parameters k30f, k30r and k35f were varied to fit the timecourse of Insulin-stimulated PTP deactivation in 3T3-L1 cells [19] and reactivation in human A431 carcinoma cells [58]. K35f is essential for a good fit.

Inhibition of PTEN by ROS is much weaker but occurs at high cytoplasmic ROS levels in 3T3 cells [20] and human neuroblastoma cells [21].

ROS transport into and out of the cell was based on the work reported in Adimora et al. in Jurkat T cells [35]. The differences in the effect of membrane permeability, SOD2 concentration and basal ROS production are shown in Additional file 2: Figure S1C-E. We remark that the molecule numbers of the ROS species in the model are scaled such that each represents 1000 real molecules (the same is true of the cytoplasmic SOD2 and PIP2/3 species).

JNK and IKK are activated by ROS directly, and by ROS deactivating the DUSP that reverses the SAPK activation [59], and deactivating the GSH that reactivates the DUSP (Figure 1E). The timescale of this activation is about one hour in response to external ROS in Fao cells [16]. The effects of ROS on activation of JNK and IKK were arranged to reproduce the data from DLD1 cells (human colon carcinoma) in Essers et al. [44]; with the attribution of the inhibition at high oxidative stress to IKK- mediated degradation of FOXO (measured in 293T and MCF-7 cells by Hu et al. [47]). IKK and JNK also lead to degradation of IRS1 through serine phosphorylation (Additional file 2: Figure S1B). Michaelis-Menten rate laws were used for these phosphorylation reactions.

#### FOXO

The FOXO model was extended based on previous work, using the same approach of scripting to generate the species and reactions required to describe the multiple FOXO species with all allowed combinations of PTMs. Compared to the previous paper, acetylation and modifications by AMPK were omitted, but modifications by Akt observed in CCL39 fibroblasts [3], by JNK in DL23 cells (related to DLD-1 human colon carcinoma) [60] and by IKK in 293T human kidney and MCF-7 human breast cancer cells [47] were included, with Akt modification altering translocation (measured in CV1 African green monkey kidney fibroblasts [46]), opposed by JNK (measured in human endothelial cells [61] and DLD1 cells [44]), and with the modifications by Akt and IKK [47] modulating degradation (for Akt, measured in HepG2 human liver carcinoma cells [45]). The model of transcription and translation was based originally on one of the effect of osmotic stress on yeast [52], but some parameters are substantially different. With the mass-action laws, the fold-change on upregulation is dependent only on the number of DNA-bound FOXO molecules and the FOXO-stimulated and basal transcription rates, with the other parameters affecting the final absolute protein and mRNA levels and the timescales. The timescale of protein regulation is several hours while the timescale of mRNA up- or down-regulation is taken as being much more rapid than this (tens of minutes); given this, it has little effect on the timescale of protein regulation. Transcriptional parameters were tuned so that SOD2 and InR showed stable cycles under physiological insulin variation (Additional file 3: Figure S2).

### Model simulations

The model reproduces previously calculated time courses of downstream activation in response to high insulin [26] (Figure 2A) and the corresponding observed dose–response curves to insulin, which were taken from experiments on rat adipocytes [36](Figure 2B). We use GLUT4 cell surface localization as a proxy for glucose uptake throughout. It will be noted that it is found experimentally that insulin-stimulated glucose uptake reaches saturation before either the Insulin receptors are fully occupied or the downstream kinases are fully active [36], though (at least in muscle) at this maximum only about 60% of GLUT4 receptors are at the cell surface [62]. It was found difficult to reproduce these observations with the original model architecture, where Akt activation and GLUT4 translocation are described by algebraic expressions depending on PIP3 levels; moreover, it was felt preferable to use differential equations to describe all reactions. By the introduction of an additional species, AS160, which mediates between Akt and GLUT4 [55-57], it was possible to obtain the dose–response curve in Figure 2B while still maintaining the fractional activations both in the unstimulated and fully stimulated states.

#### Signalling and feedback through IRS1

The insulin receptor substrate IRS1 is phosphorylated on multiple serine as well as tyrosine residues, and the serine phosphorylations tend to have the effect of reducing downstream signalling, by reducing the rate of tyrosine phosphorylation and reducing the extent to which a jointly Ser and Tyr phosphorylated IRS can signal to PI3K [16,63] or interact with the insulin receptor InR [64], though the details are complex and not fully elucidated [65,66]. We have included a single “composite” modification, IRS_PolySerP which may be present with or without Tyr phosphorylation (so there are four IRS species in the model, IRS1, IRS1_PolySerP, IRS1_TyrP and IRS1_TyrP_PolySerP, the latter representing IRS phosphorylated on both Ser and Tyr; this latter was not included in the Sedaghat model, although the others werer); IRS_PolySerP may be produced by SAPKs and the activated form of PKC (representing the PKC-ζ isoform, activated by IS [67,68]). The rates of PKC modification were set by fitting data of the timecourse of IRS tyrosine phosphorylation from human adipocytes [27]. The Serine phosphorylation by PKC is a simple representation in the model of the negative feedback loop that stabilizes the basal response of insulin signalling [69]. The result is the model IRS tyrosine phosphorylation timecourse of Figure 2C: initially it rises, within the first minute of insulin stimulation; then as PKC becomes active (after about 5 minutes) and produces serine phosphorylated forms of IRS, which do not signal downstream, IRS_TypP declines again.

Serine phosphorylation is also thought under many circumstances to lead to accelerated degradation of IRS1 [43,70,71], though this may depend on the kinases involved and on which and how many IRS1 sites are modified. Therefore we have extended the Sedaghat model by introducing synthesis and degradation reactions for the various IRS species, and have taken the Ser-p to result in accelerated degradation of IRS1 by a factor of 10, which is consistent with data for long-term insulin stimulation in rat hepatoma (H4IIE) cells [43].

IRS is also serine phosphorylated by the SAPKs, JNK and IKK in CHO and HEK 293 cells [63], 3T3-L1 cells [71] and Fao rat hepatoma cells [16]; reviewed in [66]. We have selected the rates of phosphorylation by these kinases so that IRS can be degraded on a timescale of about 4–6 hours; the outcome is shown in Additional file 2: Figure S1B.

#### ROS interactions

ROS production, detoxification and signalling in the cell is an extremely complex process and the model used here (Figure 1D and E) is much simplified. Only a “generic” ROS species representing superoxide or hydrogen peroxide is represented. Correspondingly, a single detoxifying enzyme, called SOD2, is included, which removes this in one step.

The direct interactions of oxidative stress with IS occur via the phosphatases PTEN and PTP1B. The insulin signal is transduced by tyrosine phosphorylation (in the Insulin receptor and IRS) and lipid phosphorylation (of PIP2 to PIP3), processes which are reversed by phosphatases (PTP1B and PTEN) that contain vital cysteine residues. These catalytic cysteines can be reversibly oxidised by ROS, inactivating the enzyme. Indeed, they are more susceptible to oxidation than the majority of protein cysteines precisely because they are in a protein environment that lowers their pKa in order to give them their catalytic property. Accordingly, ROS is produced by NOX enzymes in response to Insulin, facilitating the activation of IS (Figure 1D). Detailed studies in 3T3-L1 adipocytes [18,19] have revealed the extent of ROS production by NOX, and also that it deactivates after a few minutes despite continuous Insulin stimulation. The resultant ROS-mediated phosphatase inactivation is shown in Figure 2D; it is apparent that more than half the PTP is deactivated. PTEN behaves similarly but more weakly; it is not appreciably deactivated by insulin-stimulated ROS. Kinetics of NOX are shown in Additional file 2: Figure S1A. The phosphatases are reactivated on a timescale of 2–5 minutes in A431 cells [58] (the rate depends on glutathione (GSH), which can itself be oxidised by high ROS, although insulin signalling alone does not appreciably reduce the availability of glutathione). The effect of the PTP inhibition is to increase the sensitivity of insulin signalling, moving the GLUT4 dose–response curve to lower Insulin concentrations. This can be seen by setting NOX = 0 (equivalent to inhibiting it, e.g. with DPI, or introducing a large excess of antioxidants), which produces a decrease in sensitivity (Figure 2E).

ROS in the model is removed by cytoplasmic SOD2, the activity of which ensures ROS decays on a timescale of about 0.01 second. The ROS species can be exchanged across the plasma membrane at a rate 7.4 × 10^8^ m^-2^ s^-1^, (derived from measurements on Jurkat T cells [35]), giving a rate of 4.8/min with the chosen membrane area. The existence of internal detoxification enzymes means that ROS is removed as it diffuses into the cell. The background level of cytoplasmic ROS is taken to be 1 nM, and with the above diffusion rate and SOD concentration, an external concentration of 1000 times this can be tolerated before the internal concentration begins to increase appreciably (Figure 2F). The figure also shows that this threshold depends on the SOD2 concentration.

The activation of the SAPK enzymes (JNK, IKK) in the model depends directly on the concentration of cytoplasmic ROS, and by ROS deactivating the DUSP that reverses the SAPK activation, and deactivating the GSH that reactivates the DUSP (Figure 1E & D). The result is a fairly abrupt activation of JNK and IKK, shown as a function of external ROS concentration in Figure 2G. The activation of IKK is chosen to begin at a higher level of oxidative stress than JNK based on requirements of FOXO response, as described below.

JNK activation modulates the activity of the FOXO transcription factor, sending it to the nucleus and initiating transcription (for FOXO4 this occurs by direct phosphorylation [44]; similar JNK-dependent effects seem to occur through other mechanisms for the other FOXO factors, even though the phosphorylation sites are not conserved [61,72]). Conversely, Akt, activated by Insulin signalling, phosphorylates FOXO on different sites and sends it to the cytoplasm [3,46], terminating transcription. The cytoplasmic FOXO is also vulnerable to degradation. Moreover, IKK can phosphorylate FOXO, also deactivating it and accelerating its degradation [47]. In previous work [34] we produced a model of FOXO including these (and other) effects, which is here linked to the signalling pathways described above. The result is an interplay between Insulin-driven deactivation and oxidative stress driven activation, as shown in Figure 2H. The fraction of DNA bound (transcriptionally active) FOXO decreases at higher insulin, but it also increases once external ROS passes the threshold at which internal ROS begins to rise and JNK becomes active, only to fall again once IKK is also activated, as IKK-phosphorylated FOXO is expelled from the nucleus and degraded.

#### Pathway interactions and long-term processes

The above examples are all reasonably rapid processes. We now turn to those on a rather longer timescale, allowing for the interactions between the subsystems and for processes of protein synthesis/degradation to become more important. FOXO controls many genes, including InR and SOD2. The activation of stress response kinases regulating FOXO allows the system to respond to events that alter ROS through levels of SOD2. Transcriptionally regulated species in the model showed stable cycles during the physiological diurnal variation of human insulin [73], as shown in Additional file 3: Figure S2.

In Figure 3A we show the effect of long-term starvation (Insulin withdrawal) on the InR level: over the course of 48 hours it is upregulated roughly twofold. The result of this is to increase the sensitivity to insulin; as shown in the right-hand panel, the insulin dose–response curve then moves appreciably to lower concentrations. The upregulation of the insulin receptor has been shown qualitatively in C2C12 cells [74] and the twofold upregulation is in agreement with experiments in which FOXO is overexpressed in rat cardiomyocytes [53]. For SOD2, regulated similarly, there is qualitative data from MEFs and DL23 cells [60].

Figure 3B shows that the model is able to qualitatively reproduce the experimentally-observed interaction between Insulin signalling and Oxidative stress in rat skeletal muscle [17,75,76]. Relative to basal activity, hydrogen peroxide alone produces a weak activation of Glucose uptake, Insulin produces a strong uptake, but the two together, surprisingly, lead to an intermediate uptake. Examination of the intermediate components of the model show a possible explanation for this: with hydrogen peroxide alone, signalling remains inactive in the model as far as PI3K, but there is an increase in PIP3 above its basal level because of partial oxidation of PTEN (~20%) reducing its activity. This is sufficient to weakly activate Akt and its downstream targets, producing some glucose uptake. Conversely, with both Insulin and hydrogen peroxide present, activation of the SAPKs causes serine phosphorylation of IRS1, sequestering it from Tyrosine phosphorylation and hence reducing the amount available to form the IRS1-PI3K complex. The PTEN inactivation does not compensate for this, hence the effect of Insulin and hydrogen peroxide is less than insulin alone. After 240 min, there is appreciable IRS1 loss due to degradation of the serine-phosphorylated form, which affects both the Insulin-stimulated experiment and (to a greater extent) the experiments where hydrogen peroxide is present. The concentrations of hydrogen peroxide and Insulin used in the simulation were Ins = 10 nM and extracellular ROS = 5 μM. The concentrations used in the experiment are not known exactly, except that Insulin is saturating and the oxidative stress (generated by glucose oxidase) was high.

Figure 3C shows dose–response bars indicating that antioxidant defence (SOD2) can be upregulated by FOXO at a low degree of oxidative stress, but at a high level it is downregulated. There is reasonable agreement between simulation and experiment [44], though the SOD2 fold-change is rather too small and the upregulation of FOXO at low oxidative stress (caused by its nuclear location, hence escaping some degradation) is not seen in this experiment, though it was (under different experimental conditions) in the data used to parameterize the earlier FOXO model [45]. The simulation time at which these results were observed was 960 min (16 h), and the Insulin concentration was taken to be 2 nM.

**Figure 4:**
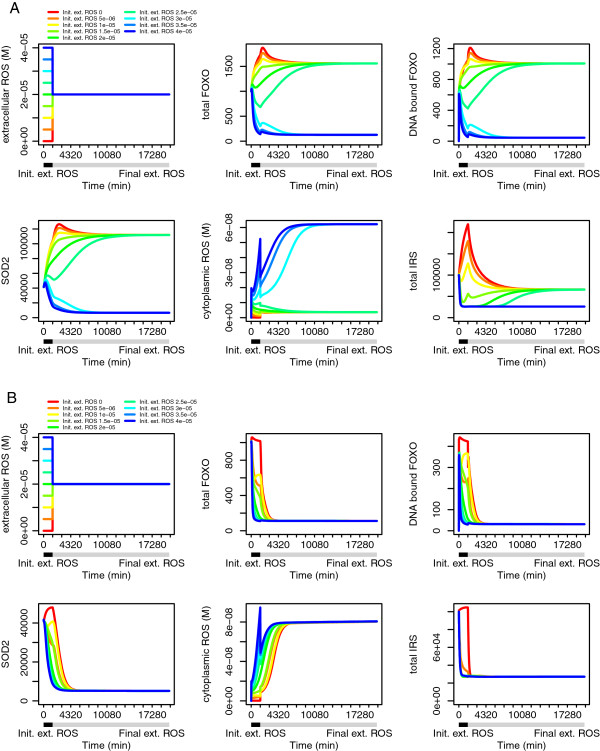
**Effects of external oxidative stress “preconditioning” on later effect of higher oxidative stress.** (**A**): Insulin = 0; effect of various initial external ROS for 1440 min, followed by moderate external ROS of 20 μM for 20000 min, on Total FOXO, DNA bound FOXO, SOD2, cytoplasmic ROS and total IRS; (**B**): as before, but with Insulin = 0.2 nM (# = 1000).

We next investigated the longer term effects of oxidative stress (Figure 4). Various initial oxidative stresses were applied for one day (1440 minutes) followed by a longer, higher basal oxidative stress lasting until 20 k minutes (approximately 14 days). With no insulin present (Figure 4A), preconditioning with various oxidative stress (including zero, corresponding to the fasting situation) upregulates antioxidants, leading to a bifurcation in the response: those that had low oxidative stress can support a higher subsequent oxidative stress than those that had a high initial stress, which downregulated antioxidants. With higher 0.2 nM insulin (Figure 4B), this bifurcation disappears; even though SOD2 may be initially downregulated to various extents, the subsequent oxidative stress leads all the systems to converge to a low SOD2 state. This contrasts somewhat with the result on FOXO localisation in Figure 2H, which was measured at t = 60 min after application of the oxidative stress. The high DNA-bound FOXO fraction at that time slightly disguises a loss of FOXO due to IKK mediated degradation.

Sensitivity analysis of the model has been carried out; the results can be found as Additional files 4, 5 and 6.

## Discussion

By integrating and extending previous work, we have produced an integrated model of insulin signalling and oxidative stress and shown that it can reproduce multiple experimental observations of the response to these stimuli in isolation and together.

With regard to the modelling approach adopted here, the treatment of the multiple PTMs in the system, especially of FOXO and IRS1, would be better treated in the rule-based modelling/kappa calculus framework currently being intensively developed [77-79] than by SBML. This would also enable treatment of the many FOXO-interacting partners. Nevertheless, we reproduced some of the flexibility of rule-based modelling by using a scripting approach to generate reactions in SBML (see Methods). In addition, FOXO mediated transcription would be better modelled within a stochastic framework, which would allow the appreciable degree of variability observed in the system on a cell-to-cell basis to become apparent. However, our emphasis was on using an integrative framework for all processes and therefore the established ODE approach was the best option.

Only a few key aspects of ROS production and detoxification have been treated in the current work. There are several chemical species of importance, particularly superoxide, produced by complexes I and III of the electron transport chain; it is a highly reactive species that is converted by superoxide dismutase (SOD) in mitochondria and cytoplasm to hydrogen peroxide, which is itself converted to water by catalases in mitochondria, cytoplasm and peroxisomes. In addition, superoxide and hydrogen peroxide can be detoxified by reacting with SH groups in GSH and Prx. Hydroxyl radicals can be produced by reaction of superoxide with Iron (II); they are extremely reactive and will react with a nearby molecule before they can be detoxified. Accordingly they are handled by keeping Iron II and superoxide levels under control and keeping iron sequestered in complexes [80]. A detailed model of the antioxidant system featuring more of these components awaits future work. In our current model, only a “generic” ROS species representing superoxide or hydrogen peroxide is represented. Correspondingly, a single detoxifying enzyme, SOD2, is included, which removes this in one step, rather than the pair of an SOD and catalase (not to mention the numerous enzymes that use dithiol-disulphide conversions to remove ROS). Recent modelling work has described interesting effects of over/underexpression of particular enzymes in this detoxification pathway [81,82]. However the approach adopted here can be justified at least in part by the fact that both SOD2 and catalase seem to be regulated in parallel by FOXO [54,83].

ROS can react with proteins, lipids and nucleic acids to form a variety of adducts which are deleterious to a greater or lesser extent. The process may or may not be reversible. This general molecular damage is not explicitly represented in this work: more detailed models of the effects of molecular damage and its resolution by protein turnover [84-86] or the DNA damage response [87,88] have been developed; in future work it would be desirable to combine these and explicitly represent the different damage inducing species superoxide, peroxide and hydroxyl radical.

Although the production of ROS may appear to be a process that is simply deleterious for an organism, the situation is complicated by the intricacy of the antioxidant system and the co-opting of ROS into signalling pathways such as IS. This underlies the complicated countervailing effects of ROS in the regulation of nutrient uptake and related pathologies, such as diabetes [22,55,89]; the present model is a first step to provide a computational framework to address this. Moreover, ROS signalling is important in the regulation of cell growth and division in general: it is becoming clear that changes in intracellular redox state coupled to ROS production are important in the regulation of almost all phases of the cell cycle, including maintenance of a quiescent state and the G1/S transition [90]. FOXO, JNK and PTP1B/PTEN all have important roles here, along with many other proteins not treated in the current work such as NRF2, p53, the CKIs, PP2A. SHP-2, LAR and Cdc25. In yeast at least, the redox potential normally cycles roughly in synchrony with the cell cycle [91]. Moreover, cellular senescence, apparently an anticancer adaptation, proceeds through a feedback loop of ROS generation [87]. Other essential processes in which ROS have a functional role include inflammation and the killing of pathogens by the macrophage oxidative burst.

The activation of SAPKs occurs by similar mechanisms to those outlined above for phosphatases: reversible cysteine oxidation leads to release of upstream activating enzymes from sequestering complexes and activates the kinases after a phosphorylation cascade; cysteine oxidation also leads to the blocking of their inactivating phosphatases (DUSPs) [59]. A more detailed model of the JNK activation cascade was made by Ferrell and co-workers [92], and it was found that in most cell types activation led to an ultrasensitive activation kinetics. Activation of IKK through TNFα was included in several models of NFκB activation [93].

Although lack of correlation of antioxidant levels in different organisms with lifespan, and the unexpected effects of knockouts and dietary antioxidant supplementations have led some authors to suggest that the free radical theory needs to be revised or rejected [14,15,94], it remains plausible that the complexity and multifaceted nature of the system could account for the observed behaviour [95]. Exploration of this will require much further development, but it has been begun in the recent work on catalase/SOD [81], dithiol antioxidants and transport of ROS [35] and antioxidant response through KEAP/NRF2 [96,97], as well as that reported here, with the introduction of the phosphatase inhibition, the interaction with SAPKs and the feedback regulation of FOXO.

The dose–response curve of FOXO-mediated antioxidant regulation is at first puzzling. Upregulation of antioxidant defences in the face of weak external oxidative stress (through a JNK-mediated mechanism) is as expected; however, high oxidative stress then leads to a downregulation again (through an IKK mediated mechanism). Here we have concentrated only on describing this effect; however evolutionarily it is hard to see what its cause could be. It may simply be that the hydrogen peroxide concentrations required to observe it (an extracellular concentration of about 20 μM) are higher than would ever be experienced by a cell in vivo, or it may be that the mechanism is cell type specific. The IKK-mediated mechanism could be activated in vivo by other, more specific, activators of NFκB signalling such as certain cytokines.

The work carried out here has been largely literature based. Better constraints could be put on the model with more detailed and more fully quantified timecourses carried out in a single (or a few) cell types. Rather than adhering to the minimal modelling approach [33,98], we have not considered multiple model architectures, and the architecture used is fairly complex, so we accept that some parameters will not be uniquely determined. Nevertheless, we have tried to avoid overfitting by extensive human intervention in the fitting, to prevent sparse data driving parameters to particularly large or small values. The sensitivity analysis indicates that all parameters have an effect on at least some species of the model and so are likely to be at least in principle identifiable, although due to the model architecture there may be some unidentifiable parameter combinations; for example the rate of FOXO synthesis is chosen to be, not a zero-order process, but the product of an E2F1 transcription factor species, whose number is assigned and does not otherwise vary, and a per-molecule rate.

We have also found it necessary to use data sources from multiple organisms and multiple tissues. Most of them are adipocyte or adipocytes like (eg 3T3-L1 cells) or skeletal muscle, the two most strongly insulin-responsive tissues, from rodent (rat/mouse) or human sources. Particularly in the FOXO part of the model, however, other cell types have been used, such as human colon and liver carcinoma (see text and Additional file 7). Many mechanisms, particularly of core insulin signalling, are strongly conserved among mammals and indeed between mammals other vertebrates and even invertebrates, but this feature of the model certainly emphasises the need for independent experimental validation.

In this model we have begun to address transcriptional feedbacks, but the simple nature of the treatment here belies the extreme difficulty of handling this: antioxidants are under the control of multiple transcription factors, but the measurements of total protein or transcript level can give no indication of which TF produced it. Hence we expect our long-time (FOXO-transcription-mediated) results will be qualitative only. The situation is further complicated by the regulation of protein synthesis at the translational level, the physical interaction of TFs with cofactors or each other, and the transcription of one TF by another [99]. For example FOXO exerts positive feedback on its own synthesis [100], and there are additional mutual regulations of FOXO, Akt and PP2A [101,102]. Achieving biological realism here is a formidable task, but could lead to a better understanding of the homeostatic and adaptive behaviours of the pathway.

What, aside from chemical detail of the ROS, is missing in the model? Clearly the system as it stands is much simplified. Other signalling pathways (p38 MAPK etc.) and TFs (NFkB [103], NRF2 [96]) may also be activated by ROS, and there are other effects, for example secretion of inflammatory cytokines. This may lead to reinforcement of inflammation by positive feedback generation of ROS, a process which the current model could be extended to describe. Moreover, a long exposure to anything more than mild oxidative stress may induce apoptosis [104]. With regard to the nutrient sensing and response pathways, a full description would require the inclusion of the mTOR (amino acid sensing) pathway along with AMPK (total energy) and possibly sensing of other nutrients, such as fatty acids, which give rise to inflammatory responses and activate PKC. Modelling of fatty acid metabolism was included in a model of glycolysis by Dash et al. [105]. Other work has already been done, which could be combined with that reported here, including for example insulin signalling combined with mTOR [29,106], AMPK [107], and the ERK pathway [31]. Significantly, other recent models of insulin signalling [33], as well as including mTOR signalling, have combined the cell signalling model with a whole body model of insulin/glucose dynamics [108].

Uptake of glucose and its transfer to mitochondria will lead to an increase in the energy production of the cell, but also the ROS output of the mitochondria. This has been neglected in the current model for which mitochondrially generated ROS is a constant. This approximation is likely to be acceptable for a cell type such as adipocytes, where the majority of ROS is signalling-associated, but would certainly need to be revised in order to extend the model to other cell types such as muscle for which mitochondrially produced ROS constitutes a larger fraction of the total. The energy demand and the ratio of glucose to other fuel sources, (particularly fatty acids, which may compete with carbohydrates [109]), will also have an influence on mitochondrially-generated ROS; see Fridlyand and Philipson for a review in the context of diabetes [110]. This may be expected to occur on a rather slower timescale than the signalling-related ROS production included in the current model, but, (even in adipocytes), will complicate the long-time effects which lead to the activation of SAPKs and the induction of insulin resistance. Hence, detailed modelling of mitochondria may be required to fully understand the role of chronic oxidative stress in the system. Mechanistic models of glycolysis [111], oxidative phosphorylation [112] and ROS production [113] in mitochondria have been produced and will be combined with the current model in future extensions.

## Conclusion

We have adapted an existing model of insulin signalling, integrated it with a model of FOXO regulation, and expanded it by introducing regulations through oxidative stress, to produce a model able to reproduce several quite complex observed effects: oxidative stress is able to activate insulin signalling, endogenous ROS increase the sensitivity of insulin signalling, and weak oxidative stress or fasting upregulates antioxidants through a homeostatic feedback mechanism, that increases the level of oxidative shock that can be withstood for a short time. Nevertheless long term high oxidative stress leads to a collapse of the oxidative stress resistance mechanism.

As outlined in the discussion, the current work addresses only a part of this very complex system and much more development, and extra experimental data, will be required to make a truly biologically realistic model of IS, FOXO and oxidative stress. Nevertheless the current work makes it apparent how valuable such an integrated model will be. A model including signalling pathways from all three food groups, as outlined above, combined with a whole body model of nutrient flux, and the interactions with oxidative stress outlined here, would be a powerful tool to address the progress of insulin resistance to metabolic syndrome and T2D [114], and how fuel use depends on overall nutrient availability and energy expenditure [115]. FOXO is known to contribute to the regulation of this [5], for example by controlling the switching between gluconeogenesis and glycolysis through transcription of PDK4, G6Pase and PEPCK; the model could be extended to include these genes downstream. It is apparent that the modelling of nutrient signalling and oxidative stress, to which the current work contributes, is coming to a level of detail that enables it to address interesting questions of life history, metabolic regulation and disease.

## Methods

Simulations were carried out with Copasi version 4.6 [116,117], using the particle number representation in deterministic mode, and analysis was done with R version 2.9.2 which was also used to produce the figures. Particle numbers were converted to concentrations for presentation. Fitting was done using Copasi’s Parameter Estimation task, and parameter sensitivities calculated with the Sensitivities tasks, using time series not steady states. The model was developed using SBML shorthand [118] in separate files corresponding to the modules of Figure 1, which were converted into SBML prior to importing into Copasi. Python scripts were used to combine the individual module files and, in the case of the FOXO module, to generate all species and reactions corresponding to the different possible combinations of PTMs and localization, as previously [34]. In essence, this results in a simulation and analysis pipeline Python - > SBML-shorthand - > SBML - > Copasi - > R. These scripts are available in supplementary material as Additional files 8 and 9, and on GitHub (https://github.com/graham1034/Smith2012_insulin_signalling), together with the model files (SBML) of each module (Additional file 10), and the complete model in two forms: initially (with particle numbers as in Table 1), which was used to produce the majority of the results (Additional file 11); and after equilibration (taken from the end of the simulation in Additional file 2: Figure S2, then further equilibrated at constant low insulin of 0.2 nM for two more days), which was used to produce the results in Figure 4 (Additional file 12). The first of these models. with particle numbers at their initial levels, has been uploaded to BIOMODELS (https://www.ebi.ac.uk/biomodels-main; identifier MODEL1212210000). The differential equations of the model are also available as Additional file 1.

## Abbreviations

FOXO: Forkhead box, type O; IRS1: Insulin Receptor Substrate 1; IS: Insulin signalling; T2D: Type 2 Diabetes; ROS: Reactive oxygen species; SAPK: Stress-activated protein kinase; JNK: c-Jun N-terminal Kinase; IKK: I-kappa-B kinase; InR: Insulin receptor; PTM: Post translational modification; PI3K: Phosphoinositide 3-Kinase; PIP2: Phosphatidylinositol 3,5-bisphosphate; PIP3: Phosphatidylinositol 3,4,5-trisphosphate; Akt (PKB): RAC-alpha serine/threonine kinase; Akt Kinase; Protein Kinase B; PKC: Protein Kinase C; GSH: Glutathione; PTEN: Phosphatase and Tensin homolog; PTP1B: Protein Tyrosine Phosphatase 1B (PTPN1); AS160: Akt substrate of 160 kDa; TBC1: domain family member 4; GLUT4: Solute carrier family 2, facilitated Glucose transporter member 4 (SLC2A4).

## Competing interests

The authors declare that they have no competing interests.

## Authors’ contributions

GS carried out the simulations, analysed the results and drafted the manuscript. DS conceived the study and helped to draft the manuscript. Both authors participated in the design of the study. Both authors read and approved the final manuscript.

## Supplementary Material

Additional file 1 Differential equations of the model, exported from Copasi and formatted with LaTeX. Click here for file

Additional file 2 **Description of Data: (A) Model kinetics of NOX activation in response to 15 min insulin, and resulting ROS production; (B) Degradation of IRS1 by prolonged high insulin signalling (black) or high extracellular ROS (red); (C) Effect of variation of basal intracellular ROS production (particle # Mt) on the dependence of intracellular ROS on extracellular ROS; (D) Effect of variation of intracellular antioxidants (particle # of SOD2) on the dependence of intracellular ROS on extracellular ROS; (E) Effect of variation of membrane permeability on the dependence of intracellular ROS on extracellular ROS.** Click here for file

Additional file 3 **Description of Data: Behaviour of key model species through 5 days (7200 minutes) of human physiological insulin variation.** Insulin data taken from Frayn et al. [73]. Click here for file

Additional file 4 Description of Data: Sensitivity of model species to parameter variation at 0.2 nM Insulin and zero external ROS. Click here for file

Additional file 5 Description of Data: Sensitivity of model species to parameter variation at 0.2 nM Insulin and 1 micromolar external ROS. Click here for file

Additional file 6 Sensitivity Analysis. Click here for file

Additional file 7: Table S5 Summary of experimental data sets used in fitting the model; see text for additional references. Click here for file

Additional file 8 **Catshorthand python script.** Description of Data: Python script to concatenate sbml-shorthand model files, putting together each section and removing species declarations that are repeated in multiple models (the one in the first file on the command line will be kept). Click here for file

Additional file 9 **Make-foxo-shorthand python script.** Description of Data: Python script to make a SBML-shorthand (.mod) file for the FOXO module by a rule-based approach, generating the species names and equations interconverting them for all combinations of the PTMs of FOXO. Click here for file

Additional file 10 **Zip archive of model sbml-shorthand files.** Description of Data: zip archive containing SBML-shorthand files (.mod) for receptor binding, akt, phosphatase, IRS, JNK and FOXO modules and for events to set five days of physiological insulin variation as in Frayn et al. m8b2_rapijf.mod is the result of assembling the files with $ catshorthand.py m8b2_recep.6.mod m8b2_akt.6.mod m8b2_phosph.6.mod m8b2_irs.6.mod m8b2_jnk.6.mod m8b2_foxo.6.mod. Click here for file

Additional file 11 **SBML file of the complete model (initial state).** SBML file of the complete model with particle numbers as in Table 1. Click here for file

Additional file 12 **SBML file of the complete model (equilibrated).** SBML file of the complete model with equilibrated particle numbers after physiol insulin cycles for 5 days then 2 more days equilibration at constant Ins=1000 (0.2 nM). Click here for file
