# Supplementary material for: Computational modelling of the regulation of Insulin signalling by oxidative stress
Source: BMC Syst Biol. 2013 May 24;7:41. doi: 10.1186/1752-0509-7-41 (PMC3668293; doi:10.1186/1752-0509-7-41)
Supplement: Additional file 1 — Differential equations of the model, exported from Copasi and formatted with LaTeX. [file 1752-0509-7-41-S1.pdf]

$$
\begin{aligned}
\frac{\mathrm{d}\left([\text{InR}]\cdot V\text{\_cellsurface}\right)}{\mathrm{d}t} &= -\left(\text{k1}\cdot[\text{Ins}]\cdot V\text{\_extracellular}\cdot[\text{InR}]\cdot V\text{\_cellsurface}\right)\\
&\quad+\left(\text{kminus1}\cdot[\text{Ins\_InR}]\cdot V\text{\_cellsurface}\right)\\
&\quad+V\text{\_cellsurface}\cdot\left(\text{kminus3}\cdot[\text{PTP1B}]\cdot V\text{\_cytoplasm}\cdot[\text{Ins\_InR\_P}]\right)\\
&\quad-\left(\text{k4}\cdot[\text{InR}]\cdot V\text{\_cellsurface}\right)\\
&\quad+\left(\text{kminus4}\cdot[\text{cytoplasm\_InR}]\cdot V\text{\_cytoplasm}\right)
\end{aligned}
$$

$$
\begin{aligned}
\frac{\mathrm{d}\left([\text{Ins\_InR}]\cdot V\text{\_cellsurface}\right)}{\mathrm{d}t} &= +\left(\text{k1}\cdot[\text{Ins}]\cdot V\text{\_extracellular}\cdot[\text{InR}]\cdot V\text{\_cellsurface}\right)\\
&\quad-\left(\text{kminus1}\cdot[\text{Ins\_InR}]\cdot V\text{\_cellsurface}\right)\\
&\quad-V\text{\_cellsurface}\cdot\left(\text{k3}\cdot[\text{Ins\_InR}]\right)
\end{aligned}
$$

$$
\begin{aligned}
\frac{\mathrm{d}\left([\text{Ins\_InR\_P}]\cdot V\text{\_cellsurface}\right)}{\mathrm{d}t} &= -\left(\text{k4prime}\cdot[\text{Ins\_InR\_P}]\cdot V\text{\_cellsurface}\right)\\
&\quad+\left(\text{kminus4prime}\cdot[\text{cytoplasm\_Ins\_InR\_P}]\cdot V\text{\_cytoplasm}\right)\\
&\quad+V\text{\_cellsurface}\cdot\left(\text{k3}\cdot[\text{Ins\_InR}]\right)\\
&\quad-\left(\text{k2}\cdot[\text{Ins}]\cdot V\text{\_extracellular}\cdot[\text{Ins\_InR\_P}]\cdot V\text{\_cellsurface}\right)\\
&\quad+\left(\text{kminus2}\cdot[\text{Ins\_2\_InR\_P}]\cdot V\text{\_cellsurface}\right)\\
&\quad-V\text{\_cellsurface}\cdot\left(\text{kminus3}\cdot[\text{PTP1B}]\cdot V\text{\_cytoplasm}\cdot[\text{Ins\_InR\_P}]\right)
\end{aligned}
$$

$$
\begin{aligned}
\frac{\mathrm{d}\left([\text{Ins\_2\_InR\_P}]\cdot V\text{\_cellsurface}\right)}{\mathrm{d}t} &= +\left(\text{k2}\cdot[\text{Ins}]\cdot V\text{\_extracellular}\cdot[\text{Ins\_InR\_P}]\cdot V\text{\_cellsurface}\right)\\
&\quad-\left(\text{kminus2}\cdot[\text{Ins\_2\_InR\_P}]\cdot V\text{\_cellsurface}\right)\\
&\quad-\left(\text{k4prime}\cdot[\text{Ins\_2\_InR\_P}]\cdot V\text{\_cellsurface}\right)\\
&\quad+\left(\text{kminus4prime}\cdot[\text{cytoplasm\_Ins\_2\_InR\_P}]\cdot V\text{\_cytoplasm}\right)
\end{aligned}
$$

$$
\begin{aligned}
\frac{\mathrm{d}\left([\text{cytoplasm\_InR}]\cdot V\text{\_cytoplasm}\right)}{\mathrm{d}t} &= +V\text{\_cytoplasm}\cdot\left(\text{k6}\cdot[\text{PTP1B}]\cdot[\text{cytoplasm\_Ins\_2\_InR\_P}]\cdot V\text{\_cytoplasm}\right)\\
&\quad+V\text{\_cytoplasm}\cdot\left(\text{k6}\cdot[\text{PTP1B}]\cdot[\text{cytoplasm\_Ins\_InR\_P}]\cdot V\text{\_cytoplasm}\right)\\
&\quad+V\text{\_cytoplasm}\cdot\left([\text{cytoplasm\_RNA\_InR}]\cdot 2.46\right)\\
&\quad-V\text{\_cytoplasm}\cdot\left(0.0044\cdot[\text{cytoplasm\_InR}]\right)\\
&\quad+\left(\text{k4}\cdot[\text{InR}]\cdot V\text{\_cellsurface}\right)\\
&\quad-\left(\text{kminus4}\cdot[\text{cytoplasm\_InR}]\cdot V\text{\_cytoplasm}\right)
\end{aligned}
$$

$$
\begin{aligned}
\frac{\mathrm{d}\left([\text{cytoplasm\_Ins\_2\_InR\_P}]\cdot V\text{\_cytoplasm}\right)}{\mathrm{d}t} &= -V\text{\_cytoplasm}\cdot\left(\text{k6}\cdot[\text{PTP1B}]\cdot[\text{cytoplasm\_Ins\_2\_InR\_P}]\cdot V\text{\_cytoplasm}\right)\\
&\quad+\left(\text{k4prime}\cdot[\text{Ins\_2\_InR\_P}]\cdot V\text{\_cellsurface}\right)\\
&\quad-\left(\text{kminus4prime}\cdot[\text{cytoplasm\_Ins\_2\_InR\_P}]\cdot V\text{\_cytoplasm}\right)
\end{aligned}
$$

$$
\begin{aligned}
\frac{\mathrm{d}\left([\text{cytoplasm\_Ins\_InR\_P}]\cdot V\text{\_cytoplasm}\right)}{\mathrm{d}t} &= +\left(\text{k4prime}\cdot[\text{Ins\_InR\_P}]\cdot V\text{\_cellsurface}\right)\\
&\quad-\left(\text{kminus4prime}\cdot[\text{cytoplasm\_Ins\_InR\_P}]\cdot V\text{\_cytoplasm}\right)\\
&\quad-V\text{\_cytoplasm}\cdot\left(\text{k6}\cdot[\text{PTP1B}]\cdot[\text{cytoplasm\_Ins\_InR\_P}]\cdot V\text{\_cytoplasm}\right)
\end{aligned}
$$

$$
\begin{aligned}
[\text{InR\_tot}] &= +\frac{\text{InR.ParticleNumber}+\text{Ins\_InR.ParticleNumber}+\text{Ins\_InR\_P.ParticleNumber}+\text{Ins\_2\_InR\_P.ParticleNumber}}{V\text{\_cytoplasm}}\\
&\quad+\frac{\text{cytoplasm\_InR.ParticleNumber}+\text{cytoplasm\_Ins\_2\_InR\_P.ParticleNumber}+\text{cytoplasm\_Ins\_InR\_P.ParticleNumber}}{V\text{\_cytoplasm}}
\end{aligned}
$$

$$
[\text{InR\_bound}] = \frac{\text{Ins\_2\_InR\_P.ParticleNumber}+\text{Ins\_InR\_P.ParticleNumber}+\text{Ins\_InR.ParticleNumber}}{V\text{\_cytoplasm}}
$$

$$
[\text{InR\_active}] = \frac{\text{Ins\_2\_InR\_P.ParticleNumber}+\text{Ins\_InR\_P.ParticleNumber}}{V\text{\_cytoplasm}}
$$

$$
\begin{aligned}
\frac{\mathrm{d}\left([\text{IRS1}]\cdot V\text{\_cytoplasm}\right)}{\mathrm{d}t} &= -V\text{\_cytoplasm}\cdot\left(\text{cyto\_vol}\cdot\frac{\text{k7}\cdot[\text{IRS1}]\cdot\left([\text{Ins\_2\_InR\_P}]\cdot V\text{\_cellsurface}+[\text{Ins\_InR\_P}]\cdot V\text{\_cellsurface}\right)}{\text{IRp}}\right)\\
&\quad+V\text{\_cytoplasm}\cdot\left(\text{cyto\_vol}\cdot\text{kminus7a}\cdot[\text{PTP1B}]\cdot[\text{IRS1\_TyrP}]\cdot V\text{\_cytoplasm}\right)\\
&\quad-V\text{\_cytoplasm}\cdot\left(\text{cyto\_vol}\cdot\text{k2psp}\cdot[\text{IRS1}]\cdot[\text{PKC\_P}]\cdot V\text{\_cytoplasm}\right)\\
&\quad+V\text{\_cytoplasm}\cdot\left(\text{cyto\_vol}\cdot\text{kminus7b}\cdot[\text{PP2A}]\cdot[\text{IRS1\_PolySerP}]\cdot V\text{\_cytoplasm}\right)\\
&\quad+V\text{\_cytoplasm}\cdot\left(\frac{\text{k\_irs1\_basal\_syn}}{V\text{\_cytoplasm}}\right)\\
&\quad-V\text{\_cytoplasm}\cdot\left(\text{k\_irs1\_basal\_degr}\cdot[\text{IRS1}]\right)\\
&\quad-V\text{\_cytoplasm}\cdot\left(\frac{\text{kcat51}\cdot[\text{IRS1}]\cdot[\text{IKK\_P}]\cdot V\text{\_cytoplasm}}{\text{Km51}+[\text{IRS1}]\cdot V\text{\_cytoplasm}}\right)\\
&\quad-V\text{\_cytoplasm}\cdot\left(\frac{\text{kcat52}\cdot[\text{IRS1}]\cdot[\text{JNK\_P}]\cdot V\text{\_cytoplasm}}{\text{Km52}+[\text{IRS1}]\cdot V\text{\_cytoplasm}}\right)
\end{aligned}
$$

$$
\begin{aligned}
\frac{\mathrm{d}\left([\text{IRS1\_TyrP}]\cdot V\text{\_cytoplasm}\right)}{\mathrm{d}t} &= +V\text{\_cytoplasm}\cdot\left(\text{cyto\_vol}\cdot\frac{\text{k7}\cdot[\text{IRS1}]\cdot\left([\text{Ins\_2\_InR\_P}]\cdot V\text{\_cellsurface}+[\text{Ins\_InR\_P}]\cdot V\text{\_cellsurface}\right)}{\text{IRp}}\right)\\
&\quad-V\text{\_cytoplasm}\cdot\left(\text{cyto\_vol}\cdot\text{kminus7a}\cdot[\text{PTP1B}]\cdot[\text{IRS1\_TyrP}]\cdot V\text{\_cytoplasm}\right)\\
&\quad-V\text{\_cytoplasm}\cdot\left(\text{cyto\_vol}\cdot\text{k2psp}\cdot[\text{IRS1\_TyrP}]\cdot[\text{PKC\_P}]\cdot V\text{\_cytoplasm}\right)\\
&\quad+V\text{\_cytoplasm}\cdot\left(\text{cyto\_vol}\cdot\text{kminus7b}\cdot[\text{PP2A}]\cdot[\text{IRS1\_TyrP\_PolySerP}]\cdot V\text{\_cytoplasm}\right)\\
&\quad-V\text{\_cytoplasm}\cdot\left(\text{cyto\_vol}\cdot\text{k8}\cdot[\text{IRS1\_TyrP}]\cdot[\text{PI3K}]\cdot V\text{\_cytoplasm}\right)\\
&\quad+V\text{\_cytoplasm}\cdot\left(\text{cyto\_vol}\cdot\text{kminus8}\cdot[\text{IRS1\_TyrP\_PI3K}]\right)\\
&\quad-V\text{\_cytoplasm}\cdot\left(\text{k\_irs1\_basal\_degr}\cdot[\text{IRS1\_TyrP}]\right)
\end{aligned}
$$

$$
\begin{aligned}
\frac{\mathrm{d}\left([\text{IRS1\_PolySerP}]\cdot V\text{\_cytoplasm}\right)}{\mathrm{d}t} &= +V\text{\_cytoplasm}\cdot\left(\text{cyto\_vol}\cdot\text{k2psp}\cdot[\text{IRS1}]\cdot[\text{PKC\_P}]\cdot V\text{\_cytoplasm}\right)\\
&\quad-V\text{\_cytoplasm}\cdot\left(\text{cyto\_vol}\cdot\text{kminus7b}\cdot[\text{PP2A}]\cdot[\text{IRS1\_PolySerP}]\cdot V\text{\_cytoplasm}\right)\\
&\quad-V\text{\_cytoplasm}\cdot\left(\text{k\_irs1\_polyserp\_degr}\cdot[\text{IRS1\_PolySerP}]\right)\\
&\quad+V\text{\_cytoplasm}\cdot\left(\frac{\text{kcat51}\cdot[\text{IRS1}]\cdot[\text{IKK\_P}]\cdot V\text{\_cytoplasm}}{\text{Km51}+[\text{IRS1}]\cdot V\text{\_cytoplasm}}\right)\\
&\quad+V\text{\_cytoplasm}\cdot\left(\frac{\text{kcat52}\cdot[\text{IRS1}]\cdot[\text{JNK\_P}]\cdot V\text{\_cytoplasm}}{\text{Km52}+[\text{IRS1}]\cdot V\text{\_cytoplasm}}\right)
\end{aligned}
$$

$$
\begin{aligned}
\frac{\mathrm{d}\left([\text{IRS1\_TyrP\_PolySerP}]\cdot V\text{\_cytoplasm}\right)}{\mathrm{d}t} &= +V\text{\_cytoplasm}\cdot\left(\text{cyto\_vol}\cdot\text{k2psp}\cdot[\text{IRS1\_TyrP}]\cdot[\text{PKC\_P}]\cdot V\text{\_cytoplasm}\right)\\
&\quad-V\text{\_cytoplasm}\cdot\left(\text{cyto\_vol}\cdot\text{kminus7b}\cdot[\text{PP2A}]\cdot[\text{IRS1\_TyrP\_PolySerP}]\cdot V\text{\_cytoplasm}\right)\\
&\quad-V\text{\_cytoplasm}\cdot\left(\text{k\_irs1\_polyserp\_degr}\cdot[\text{IRS1\_TyrP\_PolySerP}]\right)
\end{aligned}
$$

$$
[\text{IRS\_total}] = \frac{\text{IRS1.ParticleNumber}+\text{IRS1\_TyrP.ParticleNumber}+\text{IRS1\_PolySerP.ParticleNumber}+\text{IRS1\_TyrP\_PolySerP.ParticleNumber}}{V\text{\_cytoplasm}}
$$

$$
\begin{aligned}
\frac{\mathrm{d}\left([\text{PI3K}]\cdot V\text{\_cytoplasm}\right)}{\mathrm{d}t} &= -V\text{\_cytoplasm}\cdot\left(\text{cyto\_vol}\cdot\text{k8}\cdot[\text{IRS1\_TyrP}]\cdot[\text{PI3K}]\cdot V\text{\_cytoplasm}\right)\\
&\quad+V\text{\_cytoplasm}\cdot\left(\text{cyto\_vol}\cdot\text{kminus8}\cdot[\text{IRS1\_TyrP\_PI3K}]\right)
\end{aligned}
$$

$$
\begin{aligned}
\frac{\mathrm{d}\left([\text{IRS1\_TyrP\_PI3K}]\cdot V\text{\_cytoplasm}\right)}{\mathrm{d}t} &= +V\text{\_cytoplasm}\cdot\left(\text{cyto\_vol}\cdot\text{k8}\cdot[\text{IRS1\_TyrP}]\cdot[\text{PI3K}]\cdot V\text{\_cytoplasm}\right)\\
&\quad-V\text{\_cytoplasm}\cdot\left(\text{cyto\_vol}\cdot\text{kminus8}\cdot[\text{IRS1\_TyrP\_PI3K}]\right)
\end{aligned}
$$

$$
\begin{aligned}
\frac{\mathrm{d}\left([\text{PI345P3}]\cdot V\text{\_cytoplasm}\right)}{\mathrm{d}t} &= +V\text{\_cytoplasm}\cdot\left(\left(\text{k9\_basal}+\text{k9}\cdot[\text{IRS1\_TyrP\_PI3K}]\cdot V\text{\_cytoplasm}\right)\cdot[\text{PIP2}]\right)\\
&\quad-V\text{\_cytoplasm}\cdot\left(\left(\text{kminus9\_basal}+\text{kminus9}\cdot[\text{PTEN}]\cdot V\text{\_cytoplasm}\right)\cdot[\text{PI345P3}]\right)
\end{aligned}
$$

$$\frac{\mathrm{d}\left([\mathrm{PIP2}] \cdot V\_\mathrm{cytoplasm}\right)}{\mathrm{d}t} = -V\_\mathrm{cytoplasm} \cdot \left((\mathrm{k9\_basal} + \mathrm{k9} \cdot [\mathrm{IRS1\_TyrP\_PI3K}] \cdot V\_\mathrm{cytoplasm}) \cdot [\mathrm{PIP2}]\right) + V\_\mathrm{cytoplasm} \cdot \left((\mathrm{kminus9\_basal} + \mathrm{kminus9} \cdot [\mathrm{PTEN}] \cdot V\_\mathrm{cytoplasm}) \cdot [\mathrm{PI345P3}]\right)$$

$$[\mathrm{PI345P3\_mol}] = \frac{\mathrm{sc\_pip} \cdot \mathrm{PI345P3.ParticleNumber}}{V\_\mathrm{cytoplasm}}$$

$$[\mathrm{PIP2\_mol}] = \frac{\mathrm{sc\_pip} \cdot \mathrm{PIP2.ParticleNumber}}{V\_\mathrm{cytoplasm}}$$

$$\frac{\mathrm{d}\left([\mathrm{Akt}] \cdot V\_\mathrm{cytoplasm}\right)}{\mathrm{d}t} = -V\_\mathrm{cytoplasm} \cdot \left(\mathrm{k11} \cdot [\mathrm{Akt}] \cdot \begin{cases} [\mathrm{PI345P3}] \cdot V\_\mathrm{cytoplasm} - \mathrm{pip3\_basal} & \text{if } [\mathrm{PI345P3}] \cdot V\_\mathrm{cytoplasm} > \mathrm{pip3\_basal} \\ 0 & \text{otherwise} \end{cases}\right) + V\_\mathrm{cytoplasm} \cdot (\mathrm{kminus11} \cdot [\mathrm{PP2A}] \cdot [\mathrm{Akt\_P2}] \cdot V\_\mathrm{cytoplasm})$$

$$\frac{\mathrm{d}\left([\mathrm{Akt\_P2}] \cdot V\_\mathrm{cytoplasm}\right)}{\mathrm{d}t} = +V\_\mathrm{cytoplasm} \cdot \left(\mathrm{k11} \cdot [\mathrm{Akt}] \cdot \begin{cases} [\mathrm{PI345P3}] \cdot V\_\mathrm{cytoplasm} - \mathrm{pip3\_basal} & \text{if } [\mathrm{PI345P3}] \cdot V\_\mathrm{cytoplasm} > \mathrm{pip3\_basal} \\ 0 & \text{otherwise} \end{cases}\right) - V\_\mathrm{cytoplasm} \cdot (\mathrm{kminus11} \cdot [\mathrm{PP2A}] \cdot [\mathrm{Akt\_P2}] \cdot V\_\mathrm{cytoplasm})$$

$$\frac{\mathrm{d}\left([\mathrm{PKC}] \cdot V\_\mathrm{cytoplasm}\right)}{\mathrm{d}t} = -V\_\mathrm{cytoplasm} \cdot \left(\mathrm{k12} \cdot [\mathrm{PKC}] \cdot \begin{cases} [\mathrm{PI345P3}] \cdot V\_\mathrm{cytoplasm} - \mathrm{pip3\_basal} & \text{if } [\mathrm{PI345P3}] \cdot V\_\mathrm{cytoplasm} > \mathrm{pip3\_basal} \\ 0 & \text{otherwise} \end{cases}\right) + V\_\mathrm{cytoplasm} \cdot (\mathrm{kminus12} \cdot [\mathrm{PP2A}] \cdot [\mathrm{PKC\_P}] \cdot V\_\mathrm{cytoplasm})$$

$$\frac{\mathrm{d}\left([\mathrm{PKC\_P}] \cdot V\_\mathrm{cytoplasm}\right)}{\mathrm{d}t} = +V\_\mathrm{cytoplasm} \cdot \left(\mathrm{k12} \cdot [\mathrm{PKC}] \cdot \begin{cases} [\mathrm{PI345P3}] \cdot V\_\mathrm{cytoplasm} - \mathrm{pip3\_basal} & \text{if } [\mathrm{PI345P3}] \cdot V\_\mathrm{cytoplasm} > \mathrm{pip3\_basal} \\ 0 & \text{otherwise} \end{cases}\right) - V\_\mathrm{cytoplasm} \cdot (\mathrm{kminus12} \cdot [\mathrm{PP2A}] \cdot [\mathrm{PKC\_P}] \cdot V\_\mathrm{cytoplasm})$$

$$\frac{\mathrm{d}\left([\mathrm{AS160}] \cdot V\_\mathrm{cytoplasm}\right)}{\mathrm{d}t} = -V\_\mathrm{cytoplasm} \cdot (\mathrm{kr16a} \cdot [\mathrm{Akt\_P2}] \cdot [\mathrm{AS160}] \cdot V\_\mathrm{cytoplasm}) + V\_\mathrm{cytoplasm} \cdot (\mathrm{kminusr16a} \cdot [\mathrm{PP2A}] \cdot [\mathrm{AS160\_P}] \cdot V\_\mathrm{cytoplasm})$$

$$\frac{\mathrm{d}\left([\mathrm{AS160\_P}] \cdot V\_\mathrm{cytoplasm}\right)}{\mathrm{d}t} = +V\_\mathrm{cytoplasm} \cdot (\mathrm{kr16a} \cdot [\mathrm{Akt\_P2}] \cdot [\mathrm{AS160}] \cdot V\_\mathrm{cytoplasm}) - V\_\mathrm{cytoplasm} \cdot (\mathrm{kminusr16a} \cdot [\mathrm{PP2A}] \cdot [\mathrm{AS160\_P}] \cdot V\_\mathrm{cytoplasm})$$

$$\frac{\mathrm{d}\left([\mathrm{cellsurface\_GLUT4}] \cdot V\_\mathrm{cellsurface}\right)}{\mathrm{d}t} = +\left((\mathrm{k13\_basal} + \mathrm{k13} \cdot [\mathrm{AS160\_P}] \cdot V\_\mathrm{cytoplasm}) \cdot [\mathrm{cytoplasm\_GLUT4}] \cdot V\_\mathrm{cytoplasm}\right) - (\mathrm{kminus13} \cdot [\mathrm{cellsurface\_GLUT4}] \cdot V\_\mathrm{cellsurface})$$

$$\frac{\mathrm{d}\left([\mathrm{cytoplasm\_GLUT4}] \cdot V\_\mathrm{cytoplasm}\right)}{\mathrm{d}t} = -\left((\mathrm{k13\_basal} + \mathrm{k13} \cdot [\mathrm{AS160\_P}] \cdot V\_\mathrm{cytoplasm}) \cdot [\mathrm{cytoplasm\_GLUT4}] \cdot V\_\mathrm{cytoplasm}\right) + (\mathrm{kminus13} \cdot [\mathrm{cellsurface\_GLUT4}] \cdot V\_\mathrm{cellsurface})$$

$$\frac{\mathrm{d}\left([\mathrm{PTEN}] \cdot V\_\mathrm{cytoplasm}\right)}{\mathrm{d}t} = -V\_\mathrm{cytoplasm} \cdot (\mathrm{k31f} \cdot [\mathrm{PTEN}] \cdot [\mathrm{ROS}] \cdot V\_\mathrm{cytoplasm}) + V\_\mathrm{cytoplasm} \cdot (\mathrm{k31r} \cdot [\mathrm{PTEN\_ox}] \cdot [\mathrm{GSH}] \cdot V\_\mathrm{cytoplasm})$$

$$\frac{\mathrm{d}\left([\mathrm{PTP1B\_ox}] \cdot V\_\mathrm{cytoplasm}\right)}{\mathrm{d}t} = +V\_\mathrm{cytoplasm} \cdot (\mathrm{k30f} \cdot [\mathrm{PTP1B}] \cdot [\mathrm{ROS}] \cdot V\_\mathrm{cytoplasm}) - V\_\mathrm{cytoplasm} \cdot (\mathrm{k30r} \cdot [\mathrm{PTP1B\_ox}] \cdot [\mathrm{GSH}] \cdot V\_\mathrm{cytoplasm})$$

$$[\mathrm{PTP1B\_plus\_PTP1B\_ox}] = \frac{\mathrm{PTP1B.ParticleNumber} + \mathrm{PTP1B\_ox.ParticleNumber}}{V\_\mathrm{cytoplasm}}$$

$$\frac{\mathrm{d}\left([\mathrm{PTEN\_ox}] \cdot V\_\mathrm{cytoplasm}\right)}{\mathrm{d}t} = +V\_\mathrm{cytoplasm} \cdot (\mathrm{k31f} \cdot [\mathrm{PTEN}] \cdot [\mathrm{ROS}] \cdot V\_\mathrm{cytoplasm}) - V\_\mathrm{cytoplasm} \cdot (\mathrm{k31r} \cdot [\mathrm{PTEN\_ox}] \cdot [\mathrm{GSH}] \cdot V\_\mathrm{cytoplasm})$$

$$\frac{\mathrm{d}\left([\mathrm{PTP1B}] \cdot V\_\mathrm{cytoplasm}\right)}{\mathrm{d}t} = -V\_\mathrm{cytoplasm} \cdot (\mathrm{k30f} \cdot [\mathrm{PTP1B}] \cdot [\mathrm{ROS}] \cdot V\_\mathrm{cytoplasm}) + V\_\mathrm{cytoplasm} \cdot (\mathrm{k30r} \cdot [\mathrm{PTP1B\_ox}] \cdot [\mathrm{GSH}] \cdot V\_\mathrm{cytoplasm})$$

$$[\mathrm{PTEN\_plus\_PTEN\_ox}] = \frac{\mathrm{PTEN.ParticleNumber} + \mathrm{PTEN\_ox.ParticleNumber}}{V\_\mathrm{cytoplasm}}$$

$$\frac{\mathrm{d}\left([\mathrm{ROS}] \cdot V\_\mathrm{cytoplasm}\right)}{\mathrm{d}t} = +V\_\mathrm{cytoplasm} \cdot (\mathrm{k35f} \cdot [\mathrm{NOX}]) - V\_\mathrm{cytoplasm} \cdot (\mathrm{k35r} \cdot [\mathrm{ROS}] \cdot [\mathrm{cytoplasm\_SOD2}] \cdot V\_\mathrm{cytoplasm}) + V\_\mathrm{cytoplasm} \cdot (\mathrm{k36f} \cdot [\mathrm{Mt}]) + (\mathrm{k\_ros\_perm} \cdot [\mathrm{extracellular\_ROS}] \cdot V\_\mathrm{extracellular}) - \left(\mathrm{k\_ros\_perm} \cdot \frac{V\_\mathrm{extracellular}}{V\_\mathrm{cytoplasm}} \cdot [\mathrm{ROS}] \cdot V\_\mathrm{cytoplasm}\right)$$

$$\frac{\mathrm{d}\left([\mathrm{GSH}] \cdot V\_\mathrm{cytoplasm}\right)}{\mathrm{d}t} = -V\_\mathrm{cytoplasm} \cdot (\mathrm{k38f} \cdot [\mathrm{GSH}] \cdot [\mathrm{ROS}] \cdot V\_\mathrm{cytoplasm}) + V\_\mathrm{cytoplasm} \cdot (\mathrm{k38r} \cdot [\mathrm{GSSG}])$$

$$\frac{\mathrm{d}\left([\mathrm{GSSG}] \cdot V\_\mathrm{cytoplasm}\right)}{\mathrm{d}t} = +V\_\mathrm{cytoplasm} \cdot (\mathrm{k38f} \cdot [\mathrm{GSH}] \cdot [\mathrm{ROS}] \cdot V\_\mathrm{cytoplasm}) - V\_\mathrm{cytoplasm} \cdot (\mathrm{k38r} \cdot [\mathrm{GSSG}])$$

$$\frac{\mathrm{d}\left([\mathrm{cytoplasm\_SOD2}] \cdot V\_\mathrm{cytoplasm}\right)}{\mathrm{d}t} = +V\_\mathrm{cytoplasm} \cdot ([\mathrm{cytoplasm\_RNA\_SOD2}] \cdot 1.23) - V\_\mathrm{cytoplasm} \cdot (0.0019 \cdot [\mathrm{cytoplasm\_SOD2}])$$

$$\frac{\mathrm{d}\left([\mathrm{NOX\_inact}] \cdot V\_\mathrm{cytoplasm}\right)}{\mathrm{d}t} = -(\mathrm{k34f} \cdot [\mathrm{NOX\_inact}] \cdot V\_\mathrm{cytoplasm} \cdot [\mathrm{Ins}] \cdot V\_\mathrm{extracellular}) + V\_\mathrm{cytoplasm} \cdot (\mathrm{k34r2} \cdot [\mathrm{NOX}]) + V\_\mathrm{cytoplasm} \cdot (\mathrm{k34r3} \cdot [\mathrm{NOX\_deact}] \cdot [\mathrm{NOX\_deact}] \cdot V\_\mathrm{cytoplasm})$$

$$\frac{\mathrm{d}\left([\mathrm{NOX}] \cdot V\_\mathrm{cytoplasm}\right)}{\mathrm{d}t} = +(\mathrm{k34f} \cdot [\mathrm{NOX\_inact}] \cdot V\_\mathrm{cytoplasm} \cdot [\mathrm{Ins}] \cdot V\_\mathrm{extracellular}) - V\_\mathrm{cytoplasm} \cdot (\mathrm{k34r1} \cdot [\mathrm{NOX}] \cdot [\mathrm{NOX}] \cdot V\_\mathrm{cytoplasm}) - V\_\mathrm{cytoplasm} \cdot (\mathrm{k34r2} \cdot [\mathrm{NOX}])$$

$$\frac{\mathrm{d}\left([\mathrm{NOX\_deact}] \cdot V\_\mathrm{cytoplasm}\right)}{\mathrm{d}t} = +V\_\mathrm{cytoplasm} \cdot (\mathrm{k34r1} \cdot [\mathrm{NOX}] \cdot [\mathrm{NOX}] \cdot V\_\mathrm{cytoplasm}) - V\_\mathrm{cytoplasm} \cdot (\mathrm{k34r3} \cdot [\mathrm{NOX\_deact}] \cdot [\mathrm{NOX\_deact}] \cdot V\_\mathrm{cytoplasm})$$

$$[\mathrm{NOX\_total}] = \frac{\mathrm{NOX\_inact.ParticleNumber} + \mathrm{NOX.ParticleNumber} + \mathrm{NOX\_deact.ParticleNumber}}{V\_\mathrm{cytoplasm}}$$

$$\frac{\mathrm{d}\left([\mathrm{JNK\_P}] \cdot V\_\mathrm{cytoplasm}\right)}{\mathrm{d}t} = +V\_\mathrm{cytoplasm} \cdot (\mathrm{k42f} \cdot \mathrm{alpha\_ox} \cdot [\mathrm{JNK}] \cdot [\mathrm{ROS}] \cdot V\_\mathrm{cytoplasm}) - V\_\mathrm{cytoplasm} \cdot (\mathrm{k42r} \cdot [\mathrm{JNK\_P}] \cdot [\mathrm{DUSP}] \cdot V\_\mathrm{cytoplasm})$$

$$\frac{\mathrm{d}\left([\mathrm{IKK\_P}] \cdot V\_\mathrm{cytoplasm}\right)}{\mathrm{d}t} = +V\_\mathrm{cytoplasm} \cdot (\mathrm{k43f} \cdot [\mathrm{IKK}] \cdot [\mathrm{ROS}] \cdot V\_\mathrm{cytoplasm}) - V\_\mathrm{cytoplasm} \cdot (\mathrm{k43r} \cdot [\mathrm{IKK\_P}] \cdot [\mathrm{DUSP}] \cdot V\_\mathrm{cytoplasm})$$

$$\frac{\mathrm{d}\left([\mathrm{JNK}] \cdot V\_\mathrm{cytoplasm}\right)}{\mathrm{d}t} = -V\_\mathrm{cytoplasm} \cdot (\mathrm{k42f} \cdot \mathrm{alpha\_ox} \cdot [\mathrm{JNK}] \cdot [\mathrm{ROS}] \cdot V\_\mathrm{cytoplasm}) + V\_\mathrm{cytoplasm} \cdot (\mathrm{k42r} \cdot [\mathrm{JNK\_P}] \cdot [\mathrm{DUSP}] \cdot V\_\mathrm{cytoplasm})$$

$$[\mathrm{JNK\_plus\_JNK\_P}] = \frac{\mathrm{JNK.ParticleNumber} + \mathrm{JNK\_P.ParticleNumber}}{V\_\mathrm{cytoplasm}}$$

$$\frac{\mathrm{d}\left([\mathrm{IKK}] \cdot V\_\mathrm{cytoplasm}\right)}{\mathrm{d}t} = -V\_\mathrm{cytoplasm} \cdot (\mathrm{k43f} \cdot [\mathrm{IKK}] \cdot [\mathrm{ROS}] \cdot V\_\mathrm{cytoplasm}) + V\_\mathrm{cytoplasm} \cdot (\mathrm{k43r} \cdot [\mathrm{IKK\_P}] \cdot [\mathrm{DUSP}] \cdot V\_\mathrm{cytoplasm})$$

$$[\mathrm{IKK\_plus\_IKK\_P}] = \frac{\mathrm{IKK.ParticleNumber} + \mathrm{IKK\_P.ParticleNumber}}{V\_\mathrm{cytoplasm}}$$

$$\frac{\mathrm{d}\left([\mathrm{DUSP}] \cdot V\_\mathrm{cytoplasm}\right)}{\mathrm{d}t} = -V\_\mathrm{cytoplasm} \cdot (\mathrm{k32f} \cdot [\mathrm{DUSP}] \cdot [\mathrm{ROS}] \cdot V\_\mathrm{cytoplasm}) + V\_\mathrm{cytoplasm} \cdot (\mathrm{k32r} \cdot [\mathrm{DUSP\_ox}] \cdot [\mathrm{GSH}] \cdot V\_\mathrm{cytoplasm})$$

$$\frac{\mathrm{d}\left([\mathrm{DUSP\_ox}] \cdot V\_\mathrm{cytoplasm}\right)}{\mathrm{d}t} = + V\_\mathrm{cytoplasm} \cdot (\mathrm{k32f} \cdot [\mathrm{DUSP}] \cdot [\mathrm{ROS}] \cdot V\_\mathrm{cytoplasm}) - V\_\mathrm{cytoplasm} \cdot (\mathrm{k32r} \cdot [\mathrm{DUSP\_ox}] \cdot [\mathrm{GSH}] \cdot V\_\mathrm{cytoplasm})$$

$$[\mathrm{DUSP\_plus\_DUSP\_ox}] = \frac{\mathrm{DUSP.ParticleNumber} + \mathrm{DUSP\_ox.ParticleNumber}}{V\_\mathrm{cytoplasm}}$$

$$\begin{aligned}
\frac{\mathrm{d}\left([\mathrm{degr_Foxo1}] \cdot V_\mathrm{cytoplasm}\right)}{\mathrm{d}t} = & + V_\mathrm{cytoplasm} \cdot ([\mathrm{cytoplasm_Foxo1_Pa0_Pd0_Pe0_pUb1}] \cdot [\mathrm{Proteasome}] \cdot V_\mathrm{cytoplasm} \cdot 0.0001) \\
& + V_\mathrm{cytoplasm} \cdot ([\mathrm{cytoplasm_Foxo1_Pa0_Pd0_Pe1_pUb1}] \cdot [\mathrm{Proteasome}] \cdot V_\mathrm{cytoplasm} \cdot 0.0001) \\
& + V_\mathrm{cytoplasm} \cdot ([\mathrm{cytoplasm_Foxo1_Pa0_Pd1_Pe0_pUb1}] \cdot [\mathrm{Proteasome}] \cdot V_\mathrm{cytoplasm} \cdot 0.0001) \\
& + V_\mathrm{cytoplasm} \cdot ([\mathrm{cytoplasm_Foxo1_Pa0_Pd1_Pe1_pUb1}] \cdot [\mathrm{Proteasome}] \cdot V_\mathrm{cytoplasm} \cdot 0.0001) \\
& + V_\mathrm{cytoplasm} \cdot ([\mathrm{cytoplasm_Foxo1_Pa1_Pd0_Pe0_pUb1}] \cdot [\mathrm{Proteasome}] \cdot V_\mathrm{cytoplasm} \cdot 0.0001) \\
& + V_\mathrm{cytoplasm} \cdot ([\mathrm{cytoplasm_Foxo1_Pa1_Pd0_Pe1_pUb1}] \cdot [\mathrm{Proteasome}] \cdot V_\mathrm{cytoplasm} \cdot 0.0001) \\
& + V_\mathrm{cytoplasm} \cdot ([\mathrm{cytoplasm_Foxo1_Pa1_Pd1_Pe0_pUb1}] \cdot [\mathrm{Proteasome}] \cdot V_\mathrm{cytoplasm} \cdot 0.0001) \\
& + V_\mathrm{cytoplasm} \cdot ([\mathrm{cytoplasm_Foxo1_Pa1_Pd1_Pe1_pUb1}] \cdot [\mathrm{Proteasome}] \cdot V_\mathrm{cytoplasm} \cdot 0.0001)
\end{aligned}$$

$$\begin{aligned}
\frac{\mathrm{d}\left([\mathrm{cytoplasm_Foxo1_Pa0_Pd0_Pe0_pUb0}] \cdot V_\mathrm{cytoplasm}\right)}{\mathrm{d}t} = & - V_\mathrm{cytoplasm} \cdot ([\mathrm{cytoplasm_Foxo1_Pa0_Pd0_Pe0_pUb0}] \cdot [\mathrm{Akt_P2}] \cdot V_\mathrm{cytoplasm} \cdot 0.0003) \\
& - V_\mathrm{cytoplasm} \cdot ([\mathrm{cytoplasm_Foxo1_Pa0_Pd0_Pe0_pUb0}] \cdot [\mathrm{SGK}] \cdot V_\mathrm{cytoplasm} \cdot 0.0003) \\
& + V_\mathrm{cytoplasm} \cdot ([\mathrm{cytoplasm_Foxo1_Pa1_Pd0_Pe0_pUb0}] \cdot [\mathrm{PP2A}] \cdot V_\mathrm{cytoplasm} \cdot 1e-06) \\
& - V_\mathrm{cytoplasm} \cdot ([\mathrm{cytoplasm_Foxo1_Pa0_Pd0_Pe0_pUb0}] \cdot [\mathrm{IKK_P}] \cdot V_\mathrm{cytoplasm} \cdot \mathrm{by_ikk_phos_factor} \cdot 5e-05) \\
& + V_\mathrm{cytoplasm} \cdot ([\mathrm{cytoplasm_Foxo1_Pa0_Pd1_Pe0_pUb0}] \cdot [\mathrm{PP2A}] \cdot V_\mathrm{cytoplasm} \cdot 1e-06) \\
& - V_\mathrm{cytoplasm} \cdot ([\mathrm{cytoplasm_Foxo1_Pa0_Pd0_Pe0_pUb0}] \cdot [\mathrm{JNK_P}] \cdot V_\mathrm{cytoplasm} \cdot \mathrm{by_jnk_phos_factor} \cdot 5e-05) \\
& + V_\mathrm{cytoplasm} \cdot ([\mathrm{cytoplasm_Foxo1_Pa0_Pd0_Pe1_pUb0}] \cdot [\mathrm{PP2A}] \cdot V_\mathrm{cytoplasm} \cdot 1e-06) \\
& - V_\mathrm{cytoplasm} \cdot ([\mathrm{cytoplasm_Foxo1_Pa0_Pd0_Pe0_pUb0}] \cdot [\mathrm{SCF}] \cdot V_\mathrm{cytoplasm} \cdot 1e-06) \\
& + V_\mathrm{cytoplasm} \cdot ([\mathrm{E2F1}] \cdot 0.0055) \\
& - ([\mathrm{cytoplasm_Foxo1_Pa0_Pd0_Pe0_pUb0}] \cdot V_\mathrm{cytoplasm} \cdot 0.181818) \\
& + ([\mathrm{nucleus_Foxo1_Pa0_Pd0_Pe0_pUb0}] \cdot V_\mathrm{nucleus} \cdot 0.055)
\end{aligned}$$

$$\begin{aligned}
\frac{\mathrm{d}\left([\mathrm{cytoplasm_Foxo1_Pa0_Pd0_Pe0_pUb1}] \cdot V_\mathrm{cytoplasm}\right)}{\mathrm{d}t} = & - V_\mathrm{cytoplasm} \cdot ([\mathrm{cytoplasm_Foxo1_Pa0_Pd0_Pe0_pUb1}] \cdot [\mathrm{Akt_P2}] \cdot V_\mathrm{cytoplasm} \cdot 0.0003) \\
& - V_\mathrm{cytoplasm} \cdot ([\mathrm{cytoplasm_Foxo1_Pa0_Pd0_Pe0_pUb1}] \cdot [\mathrm{SGK}] \cdot V_\mathrm{cytoplasm} \cdot 0.0003) \\
& + V_\mathrm{cytoplasm} \cdot ([\mathrm{cytoplasm_Foxo1_Pa1_Pd0_Pe0_pUb1}] \cdot [\mathrm{PP2A}] \cdot V_\mathrm{cytoplasm} \cdot 1e-06) \\
& - V_\mathrm{cytoplasm} \cdot ([\mathrm{cytoplasm_Foxo1_Pa0_Pd0_Pe0_pUb1}] \cdot [\mathrm{IKK_P}] \cdot V_\mathrm{cytoplasm} \cdot \mathrm{by_ikk_phos_factor} \cdot 5e-05) \\
& + V_\mathrm{cytoplasm} \cdot ([\mathrm{cytoplasm_Foxo1_Pa0_Pd1_Pe0_pUb1}] \cdot [\mathrm{PP2A}] \cdot V_\mathrm{cytoplasm} \cdot 1e-06) \\
& - V_\mathrm{cytoplasm} \cdot ([\mathrm{cytoplasm_Foxo1_Pa0_Pd0_Pe0_pUb1}] \cdot [\mathrm{JNK_P}] \cdot V_\mathrm{cytoplasm} \cdot \mathrm{by_jnk_phos_factor} \cdot 5e-05) \\
& + V_\mathrm{cytoplasm} \cdot ([\mathrm{cytoplasm_Foxo1_Pa0_Pd0_Pe1_pUb1}] \cdot [\mathrm{PP2A}] \cdot V_\mathrm{cytoplasm} \cdot 1e-06) \\
& + V_\mathrm{cytoplasm} \cdot ([\mathrm{cytoplasm_Foxo1_Pa0_Pd0_Pe0_pUb0}] \cdot [\mathrm{SCF}] \cdot V_\mathrm{cytoplasm} \cdot 1e-06) \\
& - V_\mathrm{cytoplasm} \cdot ([\mathrm{cytoplasm_Foxo1_Pa0_Pd0_Pe0_pUb1}] \cdot [\mathrm{Proteasome}] \cdot V_\mathrm{cytoplasm} \cdot 0.0001) \\
& - ([\mathrm{cytoplasm_Foxo1_Pa0_Pd0_Pe0_pUb1}] \cdot V_\mathrm{cytoplasm} \cdot 0.181818) \\
& + ([\mathrm{nucleus_Foxo1_Pa0_Pd0_Pe0_pUb1}] \cdot V_\mathrm{nucleus} \cdot 0.055)
\end{aligned}$$

$$\begin{aligned}
\frac{\mathrm{d}\left([\mathrm{cytoplasm_Foxo1_Pa0_Pd0_Pe1_pUb0}] \cdot V_\mathrm{cytoplasm}\right)}{\mathrm{d}t} = & - V_\mathrm{cytoplasm} \cdot ([\mathrm{cytoplasm_Foxo1_Pa0_Pd0_Pe1_pUb0}] \cdot [\mathrm{Akt_P2}] \cdot V_\mathrm{cytoplasm} \cdot 0.0003) \\
& - V_\mathrm{cytoplasm} \cdot ([\mathrm{cytoplasm_Foxo1_Pa0_Pd0_Pe1_pUb0}] \cdot [\mathrm{SGK}] \cdot V_\mathrm{cytoplasm} \cdot 0.0003) \\
& + V_\mathrm{cytoplasm} \cdot ([\mathrm{cytoplasm_Foxo1_Pa1_Pd0_Pe1_pUb0}] \cdot [\mathrm{PP2A}] \cdot V_\mathrm{cytoplasm} \cdot 1e-06) \\
& - V_\mathrm{cytoplasm} \cdot ([\mathrm{cytoplasm_Foxo1_Pa0_Pd0_Pe1_pUb0}] \cdot [\mathrm{IKK_P}] \cdot V_\mathrm{cytoplasm} \cdot \mathrm{by_ikk_phos_factor} \cdot 5e-05) \\
& + V_\mathrm{cytoplasm} \cdot ([\mathrm{cytoplasm_Foxo1_Pa0_Pd1_Pe1_pUb0}] \cdot [\mathrm{PP2A}] \cdot V_\mathrm{cytoplasm} \cdot 1e-06) \\
& + V_\mathrm{cytoplasm} \cdot ([\mathrm{cytoplasm_Foxo1_Pa0_Pd0_Pe0_pUb0}] \cdot [\mathrm{JNK_P}] \cdot V_\mathrm{cytoplasm} \cdot \mathrm{by_jnk_phos_factor} \cdot 5e-05) \\
& - V_\mathrm{cytoplasm} \cdot ([\mathrm{cytoplasm_Foxo1_Pa0_Pd0_Pe1_pUb0}] \cdot [\mathrm{PP2A}] \cdot V_\mathrm{cytoplasm} \cdot 1e-06) \\
& - V_\mathrm{cytoplasm} \cdot ([\mathrm{cytoplasm_Foxo1_Pa0_Pd0_Pe1_pUb0}] \cdot [\mathrm{SCF}] \cdot V_\mathrm{cytoplasm} \cdot 1e-06) \\
& - ([\mathrm{cytoplasm_Foxo1_Pa0_Pd0_Pe1_pUb0}] \cdot V_\mathrm{cytoplasm} \cdot 1.81818) \\
& + ([\mathrm{nucleus_Foxo1_Pa0_Pd0_Pe1_pUb0}] \cdot V_\mathrm{nucleus} \cdot 0.0055)
\end{aligned}$$

$$\begin{aligned}
\frac{\mathrm{d}\left([\mathrm{cytoplasm_Foxo1_Pa0_Pd0_Pe1_pUb1}] \cdot V_\mathrm{cytoplasm}\right)}{\mathrm{d}t} = & - V_\mathrm{cytoplasm} \cdot ([\mathrm{cytoplasm_Foxo1_Pa0_Pd0_Pe1_pUb1}] \cdot [\mathrm{Akt_P2}] \cdot V_\mathrm{cytoplasm} \cdot 0.0003) \\
& - V_\mathrm{cytoplasm} \cdot ([\mathrm{cytoplasm_Foxo1_Pa0_Pd0_Pe1_pUb1}] \cdot [\mathrm{SGK}] \cdot V_\mathrm{cytoplasm} \cdot 0.0003) \\
& + V_\mathrm{cytoplasm} \cdot ([\mathrm{cytoplasm_Foxo1_Pa1_Pd0_Pe1_pUb1}] \cdot [\mathrm{PP2A}] \cdot V_\mathrm{cytoplasm} \cdot 1e-06) \\
& - V_\mathrm{cytoplasm} \cdot ([\mathrm{cytoplasm_Foxo1_Pa0_Pd0_Pe1_pUb1}] \cdot [\mathrm{IKK_P}] \cdot V_\mathrm{cytoplasm} \cdot \mathrm{by_ikk_phos_factor} \cdot 5e-05) \\
& + V_\mathrm{cytoplasm} \cdot ([\mathrm{cytoplasm_Foxo1_Pa0_Pd1_Pe1_pUb1}] \cdot [\mathrm{PP2A}] \cdot V_\mathrm{cytoplasm} \cdot 1e-06) \\
& + V_\mathrm{cytoplasm} \cdot ([\mathrm{cytoplasm_Foxo1_Pa0_Pd0_Pe0_pUb1}] \cdot [\mathrm{JNK_P}] \cdot V_\mathrm{cytoplasm} \cdot \mathrm{by_jnk_phos_factor} \cdot 5e-05) \\
& - V_\mathrm{cytoplasm} \cdot ([\mathrm{cytoplasm_Foxo1_Pa0_Pd0_Pe1_pUb1}] \cdot [\mathrm{PP2A}] \cdot V_\mathrm{cytoplasm} \cdot 1e-06) \\
& + V_\mathrm{cytoplasm} \cdot ([\mathrm{cytoplasm_Foxo1_Pa0_Pd0_Pe1_pUb0}] \cdot [\mathrm{SCF}] \cdot V_\mathrm{cytoplasm} \cdot 1e-06) \\
& - V_\mathrm{cytoplasm} \cdot ([\mathrm{cytoplasm_Foxo1_Pa0_Pd0_Pe1_pUb1}] \cdot [\mathrm{Proteasome}] \cdot V_\mathrm{cytoplasm} \cdot 0.0001) \\
& - ([\mathrm{cytoplasm_Foxo1_Pa0_Pd0_Pe1_pUb1}] \cdot V_\mathrm{cytoplasm} \cdot 1.81818) \\
& + ([\mathrm{nucleus_Foxo1_Pa0_Pd0_Pe1_pUb1}] \cdot V_\mathrm{nucleus} \cdot 0.0055)
\end{aligned}$$

$$\begin{aligned}
\frac{\mathrm{d}\left([\mathrm{cytoplasm_Foxo1_Pa0_Pd1_Pe0_pUb0}] \cdot V_\mathrm{cytoplasm}\right)}{\mathrm{d}t} = & - V_\mathrm{cytoplasm} \cdot ([\mathrm{cytoplasm_Foxo1_Pa0_Pd1_Pe0_pUb0}] \cdot [\mathrm{Akt_P2}] \cdot V_\mathrm{cytoplasm} \cdot 0.0003) \\
& - V_\mathrm{cytoplasm} \cdot ([\mathrm{cytoplasm_Foxo1_Pa0_Pd1_Pe0_pUb0}] \cdot [\mathrm{SGK}] \cdot V_\mathrm{cytoplasm} \cdot 0.0003) \\
& + V_\mathrm{cytoplasm} \cdot ([\mathrm{cytoplasm_Foxo1_Pa1_Pd1_Pe0_pUb0}] \cdot [\mathrm{PP2A}] \cdot V_\mathrm{cytoplasm} \cdot 1e-06) \\
& + V_\mathrm{cytoplasm} \cdot ([\mathrm{cytoplasm_Foxo1_Pa0_Pd0_Pe0_pUb0}] \cdot [\mathrm{IKK_P}] \cdot V_\mathrm{cytoplasm} \cdot \mathrm{by_ikk_phos_factor} \cdot 5e-05) \\
& - V_\mathrm{cytoplasm} \cdot ([\mathrm{cytoplasm_Foxo1_Pa0_Pd1_Pe0_pUb0}] \cdot [\mathrm{PP2A}] \cdot V_\mathrm{cytoplasm} \cdot 1e-06) \\
& - V_\mathrm{cytoplasm} \cdot ([\mathrm{cytoplasm_Foxo1_Pa0_Pd1_Pe0_pUb0}] \cdot [\mathrm{JNK_P}] \cdot V_\mathrm{cytoplasm} \cdot \mathrm{by_jnk_phos_factor} \cdot 5e-05) \\
& + V_\mathrm{cytoplasm} \cdot ([\mathrm{cytoplasm_Foxo1_Pa0_Pd1_Pe1_pUb0}] \cdot [\mathrm{PP2A}] \cdot V_\mathrm{cytoplasm} \cdot 1e-06) \\
& - V_\mathrm{cytoplasm} \cdot ([\mathrm{cytoplasm_Foxo1_Pa0_Pd1_Pe0_pUb0}] \cdot [\mathrm{SCF}] \cdot V_\mathrm{cytoplasm} \cdot 2.2e-05) \\
& - ([\mathrm{cytoplasm_Foxo1_Pa0_Pd1_Pe0_pUb0}] \cdot V_\mathrm{cytoplasm} \cdot 0.0909091) \\
& + ([\mathrm{nucleus_Foxo1_Pa0_Pd1_Pe0_pUb0}] \cdot V_\mathrm{nucleus} \cdot 0.55)
\end{aligned}$$

$$\begin{aligned}
\frac{\mathrm{d}\left([\mathrm{cytoplasm_Foxo1_Pa0_Pd1_Pe0_pUb1}] \cdot V_\mathrm{cytoplasm}\right)}{\mathrm{d}t} = & - V_\mathrm{cytoplasm} \cdot ([\mathrm{cytoplasm_Foxo1_Pa0_Pd1_Pe0_pUb1}] \cdot [\mathrm{Akt_P2}] \cdot V_\mathrm{cytoplasm} \cdot 0.0003) \\
& - V_\mathrm{cytoplasm} \cdot ([\mathrm{cytoplasm_Foxo1_Pa0_Pd1_Pe0_pUb1}] \cdot [\mathrm{SGK}] \cdot V_\mathrm{cytoplasm} \cdot 0.0003) \\
& + V_\mathrm{cytoplasm} \cdot ([\mathrm{cytoplasm_Foxo1_Pa1_Pd1_Pe0_pUb1}] \cdot [\mathrm{PP2A}] \cdot V_\mathrm{cytoplasm} \cdot 1e-06) \\
& + V_\mathrm{cytoplasm} \cdot ([\mathrm{cytoplasm_Foxo1_Pa0_Pd0_Pe0_pUb1}] \cdot [\mathrm{IKK_P}] \cdot V_\mathrm{cytoplasm} \cdot \mathrm{by_ikk_phos_factor} \cdot 5e-05) \\
& - V_\mathrm{cytoplasm} \cdot ([\mathrm{cytoplasm_Foxo1_Pa0_Pd1_Pe0_pUb1}] \cdot [\mathrm{PP2A}] \cdot V_\mathrm{cytoplasm} \cdot 1e-06) \\
& - V_\mathrm{cytoplasm} \cdot ([\mathrm{cytoplasm_Foxo1_Pa0_Pd1_Pe0_pUb1}] \cdot [\mathrm{JNK_P}] \cdot V_\mathrm{cytoplasm} \cdot \mathrm{by_jnk_phos_factor} \cdot 5e-05) \\
& + V_\mathrm{cytoplasm} \cdot ([\mathrm{cytoplasm_Foxo1_Pa0_Pd1_Pe1_pUb1}] \cdot [\mathrm{PP2A}] \cdot V_\mathrm{cytoplasm} \cdot 1e-06) \\
& + V_\mathrm{cytoplasm} \cdot ([\mathrm{cytoplasm_Foxo1_Pa0_Pd1_Pe0_pUb0}] \cdot [\mathrm{SCF}] \cdot V_\mathrm{cytoplasm} \cdot 2.2e-05) \\
& - V_\mathrm{cytoplasm} \cdot ([\mathrm{cytoplasm_Foxo1_Pa0_Pd1_Pe0_pUb1}] \cdot [\mathrm{Proteasome}] \cdot V_\mathrm{cytoplasm} \cdot 0.0001) \\
& - ([\mathrm{cytoplasm_Foxo1_Pa0_Pd1_Pe0_pUb1}] \cdot V_\mathrm{cytoplasm} \cdot 0.0909091) \\
& + ([\mathrm{nucleus_Foxo1_Pa0_Pd1_Pe0_pUb1}] \cdot V_\mathrm{nucleus} \cdot 0.55)
\end{aligned}$$

$$\begin{aligned}\frac{\mathrm{d}\left([\mathrm{cytoplasm_Foxo1_Pa0_Pd1_Pe1_pUb0}] \cdot V_\mathrm{cytoplasm}\right)}{\mathrm{d}t} = & - V_\mathrm{cytoplasm} \cdot ([\mathrm{cytoplasm_Foxo1_Pa0_Pd1_Pe1_pUb0}] \cdot [\mathrm{Akt_P2}] \cdot V_\mathrm{cytoplasm} \cdot 0.0003) \\
& - V_\mathrm{cytoplasm} \cdot ([\mathrm{cytoplasm_Foxo1_Pa0_Pd1_Pe1_pUb0}] \cdot [\mathrm{SGK}] \cdot V_\mathrm{cytoplasm} \cdot 0.0003) \\
& + V_\mathrm{cytoplasm} \cdot ([\mathrm{cytoplasm_Foxo1_Pa1_Pd1_Pe1_pUb0}] \cdot [\mathrm{PP2A}] \cdot V_\mathrm{cytoplasm} \cdot 1e-06) \\
& + V_\mathrm{cytoplasm} \cdot ([\mathrm{cytoplasm_Foxo1_Pa0_Pd0_Pe1_pUb0}] \cdot [\mathrm{IKK_P}] \cdot V_\mathrm{cytoplasm} \cdot \mathrm{by_ikk_phos_factor} \cdot 5e-05) \\
& - V_\mathrm{cytoplasm} \cdot ([\mathrm{cytoplasm_Foxo1_Pa0_Pd1_Pe1_pUb0}] \cdot [\mathrm{PP2A}] \cdot V_\mathrm{cytoplasm} \cdot 1e-06) \\
& + V_\mathrm{cytoplasm} \cdot ([\mathrm{cytoplasm_Foxo1_Pa0_Pd1_Pe0_pUb0}] \cdot [\mathrm{JNK_P}] \cdot V_\mathrm{cytoplasm} \cdot \mathrm{by_jnk_phos_factor} \cdot 5e-05) \\
& - V_\mathrm{cytoplasm} \cdot ([\mathrm{cytoplasm_Foxo1_Pa0_Pd1_Pe1_pUb0}] \cdot [\mathrm{PP2A}] \cdot V_\mathrm{cytoplasm} \cdot 1e-06) \\
& - V_\mathrm{cytoplasm} \cdot ([\mathrm{cytoplasm_Foxo1_Pa0_Pd1_Pe1_pUb0}] \cdot [\mathrm{SCF}] \cdot V_\mathrm{cytoplasm} \cdot 2.2e-05) \\
& - ([\mathrm{cytoplasm_Foxo1_Pa0_Pd1_Pe1_pUb0}] \cdot V_\mathrm{cytoplasm} \cdot 0.909091) \\
& + ([\mathrm{nucleus_Foxo1_Pa0_Pd1_Pe1_pUb0}] \cdot V_\mathrm{nucleus} \cdot 0.055)\end{aligned}$$

$$\begin{aligned}\frac{\mathrm{d}\left([\mathrm{cytoplasm_Foxo1_Pa0_Pd1_Pe1_pUb1}] \cdot V_\mathrm{cytoplasm}\right)}{\mathrm{d}t} = & - V_\mathrm{cytoplasm} \cdot ([\mathrm{cytoplasm_Foxo1_Pa0_Pd1_Pe1_pUb1}] \cdot [\mathrm{Akt_P2}] \cdot V_\mathrm{cytoplasm} \cdot 0.0003) \\
& - V_\mathrm{cytoplasm} \cdot ([\mathrm{cytoplasm_Foxo1_Pa0_Pd1_Pe1_pUb1}] \cdot [\mathrm{SGK}] \cdot V_\mathrm{cytoplasm} \cdot 0.0003) \\
& + V_\mathrm{cytoplasm} \cdot ([\mathrm{cytoplasm_Foxo1_Pa1_Pd1_Pe1_pUb1}] \cdot [\mathrm{PP2A}] \cdot V_\mathrm{cytoplasm} \cdot 1e-06) \\
& + V_\mathrm{cytoplasm} \cdot ([\mathrm{cytoplasm_Foxo1_Pa0_Pd0_Pe1_pUb1}] \cdot [\mathrm{IKK_P}] \cdot V_\mathrm{cytoplasm} \cdot \mathrm{by_ikk_phos_factor} \cdot 5e-05) \\
& - V_\mathrm{cytoplasm} \cdot ([\mathrm{cytoplasm_Foxo1_Pa0_Pd1_Pe1_pUb1}] \cdot [\mathrm{PP2A}] \cdot V_\mathrm{cytoplasm} \cdot 1e-06) \\
& + V_\mathrm{cytoplasm} \cdot ([\mathrm{cytoplasm_Foxo1_Pa0_Pd1_Pe0_pUb1}] \cdot [\mathrm{JNK_P}] \cdot V_\mathrm{cytoplasm} \cdot \mathrm{by_jnk_phos_factor} \cdot 5e-05) \\
& - V_\mathrm{cytoplasm} \cdot ([\mathrm{cytoplasm_Foxo1_Pa0_Pd1_Pe1_pUb1}] \cdot [\mathrm{PP2A}] \cdot V_\mathrm{cytoplasm} \cdot 1e-06) \\
& + V_\mathrm{cytoplasm} \cdot ([\mathrm{cytoplasm_Foxo1_Pa0_Pd1_Pe1_pUb0}] \cdot [\mathrm{SCF}] \cdot V_\mathrm{cytoplasm} \cdot 2.2e-05) \\
& - V_\mathrm{cytoplasm} \cdot ([\mathrm{cytoplasm_Foxo1_Pa0_Pd1_Pe1_pUb1}] \cdot [\mathrm{Proteasome}] \cdot V_\mathrm{cytoplasm} \cdot 0.0001) \\
& - ([\mathrm{cytoplasm_Foxo1_Pa0_Pd1_Pe1_pUb1}] \cdot V_\mathrm{cytoplasm} \cdot 0.909091) \\
& + ([\mathrm{nucleus_Foxo1_Pa0_Pd1_Pe1_pUb1}] \cdot V_\mathrm{nucleus} \cdot 0.055)\end{aligned}$$

$$\begin{aligned}\frac{\mathrm{d}\left([\mathrm{cytoplasm_Foxo1_Pa1_Pd0_Pe0_pUb0}] \cdot V_\mathrm{cytoplasm}\right)}{\mathrm{d}t} = & + V_\mathrm{cytoplasm} \cdot ([\mathrm{cytoplasm_Foxo1_Pa0_Pd0_Pe0_pUb0}] \cdot [\mathrm{Akt_P2}] \cdot V_\mathrm{cytoplasm} \cdot 0.0003) \\
& + V_\mathrm{cytoplasm} \cdot ([\mathrm{cytoplasm_Foxo1_Pa0_Pd0_Pe0_pUb0}] \cdot [\mathrm{SGK}] \cdot V_\mathrm{cytoplasm} \cdot 0.0003) \\
& - V_\mathrm{cytoplasm} \cdot ([\mathrm{cytoplasm_Foxo1_Pa1_Pd0_Pe0_pUb0}] \cdot [\mathrm{PP2A}] \cdot V_\mathrm{cytoplasm} \cdot 1e-06) \\
& - V_\mathrm{cytoplasm} \cdot ([\mathrm{cytoplasm_Foxo1_Pa1_Pd0_Pe0_pUb0}] \cdot [\mathrm{IKK_P}] \cdot V_\mathrm{cytoplasm} \cdot \mathrm{by_ikk_phos_factor} \cdot 5e-05) \\
& + V_\mathrm{cytoplasm} \cdot ([\mathrm{cytoplasm_Foxo1_Pa1_Pd1_Pe0_pUb0}] \cdot [\mathrm{PP2A}] \cdot V_\mathrm{cytoplasm} \cdot 1e-06) \\
& - V_\mathrm{cytoplasm} \cdot ([\mathrm{cytoplasm_Foxo1_Pa1_Pd0_Pe0_pUb0}] \cdot [\mathrm{JNK_P}] \cdot V_\mathrm{cytoplasm} \cdot \mathrm{by_jnk_phos_factor} \cdot 5e-05) \\
& + V_\mathrm{cytoplasm} \cdot ([\mathrm{cytoplasm_Foxo1_Pa1_Pd0_Pe1_pUb0}] \cdot [\mathrm{PP2A}] \cdot V_\mathrm{cytoplasm} \cdot 1e-06) \\
& - V_\mathrm{cytoplasm} \cdot ([\mathrm{cytoplasm_Foxo1_Pa1_Pd0_Pe0_pUb0}] \cdot [\mathrm{SCF}] \cdot V_\mathrm{cytoplasm} \cdot 3e-06) \\
& - ([\mathrm{cytoplasm_Foxo1_Pa1_Pd0_Pe0_pUb0}] \cdot V_\mathrm{cytoplasm} \cdot 0.0181818) \\
& + ([\mathrm{nucleus_Foxo1_Pa1_Pd0_Pe0_pUb0}] \cdot V_\mathrm{nucleus} \cdot 0.55)\end{aligned}$$

$$\begin{aligned}\frac{\mathrm{d}\left([\mathrm{cytoplasm_Foxo1_Pa1_Pd0_Pe0_pUb1}] \cdot V_\mathrm{cytoplasm}\right)}{\mathrm{d}t} = & + V_\mathrm{cytoplasm} \cdot ([\mathrm{cytoplasm_Foxo1_Pa0_Pd0_Pe0_pUb1}] \cdot [\mathrm{Akt_P2}] \cdot V_\mathrm{cytoplasm} \cdot 0.0003) \\
& + V_\mathrm{cytoplasm} \cdot ([\mathrm{cytoplasm_Foxo1_Pa0_Pd0_Pe0_pUb1}] \cdot [\mathrm{SGK}] \cdot V_\mathrm{cytoplasm} \cdot 0.0003) \\
& - V_\mathrm{cytoplasm} \cdot ([\mathrm{cytoplasm_Foxo1_Pa1_Pd0_Pe0_pUb1}] \cdot [\mathrm{PP2A}] \cdot V_\mathrm{cytoplasm} \cdot 1e-06) \\
& - V_\mathrm{cytoplasm} \cdot ([\mathrm{cytoplasm_Foxo1_Pa1_Pd0_Pe0_pUb1}] \cdot [\mathrm{IKK_P}] \cdot V_\mathrm{cytoplasm} \cdot \mathrm{by_ikk_phos_factor} \cdot 5e-05) \\
& + V_\mathrm{cytoplasm} \cdot ([\mathrm{cytoplasm_Foxo1_Pa1_Pd1_Pe0_pUb1}] \cdot [\mathrm{PP2A}] \cdot V_\mathrm{cytoplasm} \cdot 1e-06) \\
& - V_\mathrm{cytoplasm} \cdot ([\mathrm{cytoplasm_Foxo1_Pa1_Pd0_Pe0_pUb1}] \cdot [\mathrm{JNK_P}] \cdot V_\mathrm{cytoplasm} \cdot \mathrm{by_jnk_phos_factor} \cdot 5e-05) \\
& + V_\mathrm{cytoplasm} \cdot ([\mathrm{cytoplasm_Foxo1_Pa1_Pd0_Pe1_pUb1}] \cdot [\mathrm{PP2A}] \cdot V_\mathrm{cytoplasm} \cdot 1e-06) \\
& + V_\mathrm{cytoplasm} \cdot ([\mathrm{cytoplasm_Foxo1_Pa1_Pd0_Pe0_pUb0}] \cdot [\mathrm{SCF}] \cdot V_\mathrm{cytoplasm} \cdot 3e-06) \\
& - V_\mathrm{cytoplasm} \cdot ([\mathrm{cytoplasm_Foxo1_Pa1_Pd0_Pe0_pUb1}] \cdot [\mathrm{Proteasome}] \cdot V_\mathrm{cytoplasm} \cdot 0.0001) \\
& - ([\mathrm{cytoplasm_Foxo1_Pa1_Pd0_Pe0_pUb1}] \cdot V_\mathrm{cytoplasm} \cdot 0.0181818) \\
& + ([\mathrm{nucleus_Foxo1_Pa1_Pd0_Pe0_pUb1}] \cdot V_\mathrm{nucleus} \cdot 0.55)\end{aligned}$$

$$\begin{aligned}\frac{\mathrm{d}\left([\mathrm{cytoplasm_Foxo1_Pa1_Pd0_Pe1_pUb0}] \cdot V_\mathrm{cytoplasm}\right)}{\mathrm{d}t} = & - ([\mathrm{cytoplasm_Foxo1_Pa1_Pd0_Pe1_pUb0}] \cdot V_\mathrm{cytoplasm} \cdot 0.181818) \\
& + ([\mathrm{nucleus_Foxo1_Pa1_Pd0_Pe1_pUb0}] \cdot V_\mathrm{nucleus} \cdot 0.055) \\
& + V_\mathrm{cytoplasm} \cdot ([\mathrm{cytoplasm_Foxo1_Pa0_Pd0_Pe1_pUb0}] \cdot [\mathrm{Akt_P2}] \cdot V_\mathrm{cytoplasm} \cdot 0.0003) \\
& + V_\mathrm{cytoplasm} \cdot ([\mathrm{cytoplasm_Foxo1_Pa0_Pd0_Pe1_pUb0}] \cdot [\mathrm{SGK}] \cdot V_\mathrm{cytoplasm} \cdot 0.0003) \\
& - V_\mathrm{cytoplasm} \cdot ([\mathrm{cytoplasm_Foxo1_Pa1_Pd0_Pe1_pUb0}] \cdot [\mathrm{PP2A}] \cdot V_\mathrm{cytoplasm} \cdot 1e-06) \\
& - V_\mathrm{cytoplasm} \cdot ([\mathrm{cytoplasm_Foxo1_Pa1_Pd0_Pe1_pUb0}] \cdot [\mathrm{IKK_P}] \cdot V_\mathrm{cytoplasm} \cdot \mathrm{by_ikk_phos_factor} \cdot 5e-05) \\
& + V_\mathrm{cytoplasm} \cdot ([\mathrm{cytoplasm_Foxo1_Pa1_Pd1_Pe1_pUb0}] \cdot [\mathrm{PP2A}] \cdot V_\mathrm{cytoplasm} \cdot 1e-06) \\
& + V_\mathrm{cytoplasm} \cdot ([\mathrm{cytoplasm_Foxo1_Pa1_Pd0_Pe0_pUb0}] \cdot [\mathrm{JNK_P}] \cdot V_\mathrm{cytoplasm} \cdot \mathrm{by_jnk_phos_factor} \cdot 5e-05) \\
& - V_\mathrm{cytoplasm} \cdot ([\mathrm{cytoplasm_Foxo1_Pa1_Pd0_Pe1_pUb0}] \cdot [\mathrm{PP2A}] \cdot V_\mathrm{cytoplasm} \cdot 1e-06) \\
& - V_\mathrm{cytoplasm} \cdot ([\mathrm{cytoplasm_Foxo1_Pa1_Pd0_Pe1_pUb0}] \cdot [\mathrm{SCF}] \cdot V_\mathrm{cytoplasm} \cdot 3e-06)\end{aligned}$$

$$\begin{aligned}\frac{\mathrm{d}\left([\mathrm{cytoplasm_Foxo1_Pa1_Pd0_Pe1_pUb1}] \cdot V_\mathrm{cytoplasm}\right)}{\mathrm{d}t} = & - ([\mathrm{cytoplasm_Foxo1_Pa1_Pd0_Pe1_pUb1}] \cdot V_\mathrm{cytoplasm} \cdot 0.181818) \\
& + ([\mathrm{nucleus_Foxo1_Pa1_Pd0_Pe1_pUb1}] \cdot V_\mathrm{nucleus} \cdot 0.055) \\
& + V_\mathrm{cytoplasm} \cdot ([\mathrm{cytoplasm_Foxo1_Pa0_Pd0_Pe1_pUb1}] \cdot [\mathrm{Akt_P2}] \cdot V_\mathrm{cytoplasm} \cdot 0.0003) \\
& + V_\mathrm{cytoplasm} \cdot ([\mathrm{cytoplasm_Foxo1_Pa0_Pd0_Pe1_pUb1}] \cdot [\mathrm{SGK}] \cdot V_\mathrm{cytoplasm} \cdot 0.0003) \\
& - V_\mathrm{cytoplasm} \cdot ([\mathrm{cytoplasm_Foxo1_Pa1_Pd0_Pe1_pUb1}] \cdot [\mathrm{PP2A}] \cdot V_\mathrm{cytoplasm} \cdot 1e-06) \\
& - V_\mathrm{cytoplasm} \cdot ([\mathrm{cytoplasm_Foxo1_Pa1_Pd0_Pe1_pUb1}] \cdot [\mathrm{IKK_P}] \cdot V_\mathrm{cytoplasm} \cdot \mathrm{by_ikk_phos_factor} \cdot 5e-05) \\
& + V_\mathrm{cytoplasm} \cdot ([\mathrm{cytoplasm_Foxo1_Pa1_Pd1_Pe1_pUb1}] \cdot [\mathrm{PP2A}] \cdot V_\mathrm{cytoplasm} \cdot 1e-06) \\
& + V_\mathrm{cytoplasm} \cdot ([\mathrm{cytoplasm_Foxo1_Pa1_Pd0_Pe0_pUb1}] \cdot [\mathrm{JNK_P}] \cdot V_\mathrm{cytoplasm} \cdot \mathrm{by_jnk_phos_factor} \cdot 5e-05) \\
& - V_\mathrm{cytoplasm} \cdot ([\mathrm{cytoplasm_Foxo1_Pa1_Pd0_Pe1_pUb1}] \cdot [\mathrm{PP2A}] \cdot V_\mathrm{cytoplasm} \cdot 1e-06) \\
& + V_\mathrm{cytoplasm} \cdot ([\mathrm{cytoplasm_Foxo1_Pa1_Pd0_Pe1_pUb0}] \cdot [\mathrm{SCF}] \cdot V_\mathrm{cytoplasm} \cdot 3e-06) \\
& - V_\mathrm{cytoplasm} \cdot ([\mathrm{cytoplasm_Foxo1_Pa1_Pd0_Pe1_pUb1}] \cdot [\mathrm{Proteasome}] \cdot V_\mathrm{cytoplasm} \cdot 0.0001)\end{aligned}$$

$$\begin{aligned}\frac{\mathrm{d}\left([\mathrm{cytoplasm_Foxo1_Pa1_Pd1_Pe0_pUb0}] \cdot V_\mathrm{cytoplasm}\right)}{\mathrm{d}t} = & - ([\mathrm{cytoplasm_Foxo1_Pa1_Pd1_Pe0_pUb0}] \cdot V_\mathrm{cytoplasm} \cdot 0.00909091) \\
& + ([\mathrm{nucleus_Foxo1_Pa1_Pd1_Pe0_pUb0}] \cdot V_\mathrm{nucleus} \cdot 5.5) \\
& + V_\mathrm{cytoplasm} \cdot ([\mathrm{cytoplasm_Foxo1_Pa0_Pd1_Pe0_pUb0}] \cdot [\mathrm{Akt_P2}] \cdot V_\mathrm{cytoplasm} \cdot 0.0003) \\
& + V_\mathrm{cytoplasm} \cdot ([\mathrm{cytoplasm_Foxo1_Pa0_Pd1_Pe0_pUb0}] \cdot [\mathrm{SGK}] \cdot V_\mathrm{cytoplasm} \cdot 0.0003) \\
& - V_\mathrm{cytoplasm} \cdot ([\mathrm{cytoplasm_Foxo1_Pa1_Pd1_Pe0_pUb0}] \cdot [\mathrm{PP2A}] \cdot V_\mathrm{cytoplasm} \cdot 1e-06) \\
& + V_\mathrm{cytoplasm} \cdot ([\mathrm{cytoplasm_Foxo1_Pa1_Pd0_Pe0_pUb0}] \cdot [\mathrm{IKK_P}] \cdot V_\mathrm{cytoplasm} \cdot \mathrm{by_ikk_phos_factor} \cdot 5e-05) \\
& - V_\mathrm{cytoplasm} \cdot ([\mathrm{cytoplasm_Foxo1_Pa1_Pd1_Pe0_pUb0}] \cdot [\mathrm{PP2A}] \cdot V_\mathrm{cytoplasm} \cdot 1e-06) \\
& - V_\mathrm{cytoplasm} \cdot ([\mathrm{cytoplasm_Foxo1_Pa1_Pd1_Pe0_pUb0}] \cdot [\mathrm{JNK_P}] \cdot V_\mathrm{cytoplasm} \cdot \mathrm{by_jnk_phos_factor} \cdot 5e-05) \\
& + V_\mathrm{cytoplasm} \cdot ([\mathrm{cytoplasm_Foxo1_Pa1_Pd1_Pe1_pUb0}] \cdot [\mathrm{PP2A}] \cdot V_\mathrm{cytoplasm} \cdot 1e-06) \\
& - V_\mathrm{cytoplasm} \cdot ([\mathrm{cytoplasm_Foxo1_Pa1_Pd1_Pe0_pUb0}] \cdot [\mathrm{SCF}] \cdot V_\mathrm{cytoplasm} \cdot 6.6e-05)\end{aligned}$$

$$\begin{aligned}\frac{\mathrm{d}\left([\mathrm{cytoplasm_Foxo1_Pa1_Pd1_Pe0_pUb1}] \cdot V_\mathrm{cytoplasm}\right)}{\mathrm{d}t} = & - ([\mathrm{cytoplasm_Foxo1_Pa1_Pd1_Pe0_pUb1}] \cdot V_\mathrm{cytoplasm} \cdot 0.00909091) \\
& + ([\mathrm{nucleus_Foxo1_Pa1_Pd1_Pe0_pUb1}] \cdot V_\mathrm{nucleus} \cdot 5.5) \\
& + V_\mathrm{cytoplasm} \cdot ([\mathrm{cytoplasm_Foxo1_Pa0_Pd1_Pe0_pUb1}] \cdot [\mathrm{Akt_P2}] \cdot V_\mathrm{cytoplasm} \cdot 0.0003) \\
& + V_\mathrm{cytoplasm} \cdot ([\mathrm{cytoplasm_Foxo1_Pa0_Pd1_Pe0_pUb1}] \cdot [\mathrm{SGK}] \cdot V_\mathrm{cytoplasm} \cdot 0.0003)\end{aligned}$$

$$- V\text{\_cytoplasm} \cdot ([\text{cytoplasm\_Foxo1\_Pa1\_Pd1\_Pe0\_pUb1}] \cdot [\text{PP2A}] \cdot V\text{\_cytoplasm} \cdot 1e-06)$$
$$+ V\text{\_cytoplasm} \cdot ([\text{cytoplasm\_Foxo1\_Pa1\_Pd0\_Pe0\_pUb1}] \cdot [\text{IKK\_P}] \cdot V\text{\_cytoplasm} \cdot \text{by\_ikk\_phos\_factor} \cdot 5e-05)$$
$$- V\text{\_cytoplasm} \cdot ([\text{cytoplasm\_Foxo1\_Pa1\_Pd1\_Pe0\_pUb1}] \cdot [\text{PP2A}] \cdot V\text{\_cytoplasm} \cdot 1e-06)$$
$$- V\text{\_cytoplasm} \cdot ([\text{cytoplasm\_Foxo1\_Pa1\_Pd1\_Pe0\_pUb1}] \cdot [\text{JNK\_P}] \cdot V\text{\_cytoplasm} \cdot \text{by\_jnk\_phos\_factor} \cdot 5e-05)$$
$$+ V\text{\_cytoplasm} \cdot ([\text{cytoplasm\_Foxo1\_Pa1\_Pd1\_Pe1\_pUb1}] \cdot [\text{PP2A}] \cdot V\text{\_cytoplasm} \cdot 1e-06)$$
$$+ V\text{\_cytoplasm} \cdot ([\text{cytoplasm\_Foxo1\_Pa1\_Pd1\_Pe0\_pUb0}] \cdot [\text{SCF}] \cdot V\text{\_cytoplasm} \cdot 6.6e-05)$$
$$- V\text{\_cytoplasm} \cdot ([\text{cytoplasm\_Foxo1\_Pa1\_Pd1\_Pe0\_pUb1}] \cdot [\text{Proteasome}] \cdot V\text{\_cytoplasm} \cdot 0.0001)$$

$$\frac{\mathrm{d}\,([\text{cytoplasm\_Foxo1\_Pa1\_Pd1\_Pe1\_pUb0}] \cdot V\text{\_cytoplasm})}{\mathrm{d}t} = - ([\text{cytoplasm\_Foxo1\_Pa1\_Pd1\_Pe1\_pUb0}] \cdot V\text{\_cytoplasm} \cdot 0.0909091)$$
$$+ ([\text{nucleus\_Foxo1\_Pa1\_Pd1\_Pe1\_pUb0}] \cdot V\text{\_nucleus} \cdot 0.55)$$
$$+ V\text{\_cytoplasm} \cdot ([\text{cytoplasm\_Foxo1\_Pa0\_Pd1\_Pe1\_pUb0}] \cdot [\text{Akt\_P2}] \cdot V\text{\_cytoplasm} \cdot 0.0003)$$
$$+ V\text{\_cytoplasm} \cdot ([\text{cytoplasm\_Foxo1\_Pa0\_Pd1\_Pe1\_pUb0}] \cdot [\text{SGK}] \cdot V\text{\_cytoplasm} \cdot 0.0003)$$
$$- V\text{\_cytoplasm} \cdot ([\text{cytoplasm\_Foxo1\_Pa1\_Pd1\_Pe1\_pUb0}] \cdot [\text{PP2A}] \cdot V\text{\_cytoplasm} \cdot 1e-06)$$
$$+ V\text{\_cytoplasm} \cdot ([\text{cytoplasm\_Foxo1\_Pa1\_Pd0\_Pe1\_pUb0}] \cdot [\text{IKK\_P}] \cdot V\text{\_cytoplasm} \cdot \text{by\_ikk\_phos\_factor} \cdot 5e-05)$$
$$- V\text{\_cytoplasm} \cdot ([\text{cytoplasm\_Foxo1\_Pa1\_Pd1\_Pe1\_pUb0}] \cdot [\text{PP2A}] \cdot V\text{\_cytoplasm} \cdot 1e-06)$$
$$+ V\text{\_cytoplasm} \cdot ([\text{cytoplasm\_Foxo1\_Pa1\_Pd1\_Pe0\_pUb0}] \cdot [\text{JNK\_P}] \cdot V\text{\_cytoplasm} \cdot \text{by\_jnk\_phos\_factor} \cdot 5e-05)$$
$$- V\text{\_cytoplasm} \cdot ([\text{cytoplasm\_Foxo1\_Pa1\_Pd1\_Pe1\_pUb0}] \cdot [\text{PP2A}] \cdot V\text{\_cytoplasm} \cdot 1e-06)$$
$$- V\text{\_cytoplasm} \cdot ([\text{cytoplasm\_Foxo1\_Pa1\_Pd1\_Pe1\_pUb0}] \cdot [\text{SCF}] \cdot V\text{\_cytoplasm} \cdot 6.6e-05)$$

$$\frac{\mathrm{d}\,([\text{cytoplasm\_Foxo1\_Pa1\_Pd1\_Pe1\_pUb1}] \cdot V\text{\_cytoplasm})}{\mathrm{d}t} = - ([\text{cytoplasm\_Foxo1\_Pa1\_Pd1\_Pe1\_pUb1}] \cdot V\text{\_cytoplasm} \cdot 0.0909091)$$
$$+ ([\text{nucleus\_Foxo1\_Pa1\_Pd1\_Pe1\_pUb1}] \cdot V\text{\_nucleus} \cdot 0.55)$$
$$+ V\text{\_cytoplasm} \cdot ([\text{cytoplasm\_Foxo1\_Pa0\_Pd1\_Pe1\_pUb1}] \cdot [\text{Akt\_P2}] \cdot V\text{\_cytoplasm} \cdot 0.0003)$$
$$+ V\text{\_cytoplasm} \cdot ([\text{cytoplasm\_Foxo1\_Pa0\_Pd1\_Pe1\_pUb1}] \cdot [\text{SGK}] \cdot V\text{\_cytoplasm} \cdot 0.0003)$$
$$- V\text{\_cytoplasm} \cdot ([\text{cytoplasm\_Foxo1\_Pa1\_Pd1\_Pe1\_pUb1}] \cdot [\text{PP2A}] \cdot V\text{\_cytoplasm} \cdot 1e-06)$$
$$+ V\text{\_cytoplasm} \cdot ([\text{cytoplasm\_Foxo1\_Pa1\_Pd0\_Pe1\_pUb1}] \cdot [\text{IKK\_P}] \cdot V\text{\_cytoplasm} \cdot \text{by\_ikk\_phos\_factor} \cdot 5e-05)$$
$$- V\text{\_cytoplasm} \cdot ([\text{cytoplasm\_Foxo1\_Pa1\_Pd1\_Pe1\_pUb1}] \cdot [\text{PP2A}] \cdot V\text{\_cytoplasm} \cdot 1e-06)$$
$$+ V\text{\_cytoplasm} \cdot ([\text{cytoplasm\_Foxo1\_Pa1\_Pd1\_Pe0\_pUb1}] \cdot [\text{JNK\_P}] \cdot V\text{\_cytoplasm} \cdot \text{by\_jnk\_phos\_factor} \cdot 5e-05)$$
$$- V\text{\_cytoplasm} \cdot ([\text{cytoplasm\_Foxo1\_Pa1\_Pd1\_Pe1\_pUb1}] \cdot [\text{PP2A}] \cdot V\text{\_cytoplasm} \cdot 1e-06)$$
$$+ V\text{\_cytoplasm} \cdot ([\text{cytoplasm\_Foxo1\_Pa1\_Pd1\_Pe1\_pUb0}] \cdot [\text{SCF}] \cdot V\text{\_cytoplasm} \cdot 6.6e-05)$$
$$- V\text{\_cytoplasm} \cdot ([\text{cytoplasm\_Foxo1\_Pa1\_Pd1\_Pe1\_pUb1}] \cdot [\text{Proteasome}] \cdot V\text{\_cytoplasm} \cdot 0.0001)$$

$$\frac{\mathrm{d}\,([\text{cytoplasm\_RNA\_InR}] \cdot V\text{\_cytoplasm})}{\mathrm{d}t} = + ([\text{nucleus\_RNA\_InR}] \cdot V\text{\_nucleus} \cdot 0.22)$$
$$- V\text{\_cytoplasm} \cdot (5.622 \cdot [\text{cytoplasm\_RNA\_InR}])$$

$$\frac{\mathrm{d}\,([\text{cytoplasm\_RNA\_SOD2}] \cdot V\text{\_cytoplasm})}{\mathrm{d}t} = + ([\text{nucleus\_RNA\_SOD2}] \cdot V\text{\_nucleus} \cdot 0.22)$$
$$- V\text{\_cytoplasm} \cdot (5.622 \cdot [\text{cytoplasm\_RNA\_SOD2}])$$

$$\frac{\mathrm{d}\,([\text{nucleus\_Foxo1\_Pa0\_Pd0\_Pe0\_pUb0}] \cdot V\text{\_nucleus})}{\mathrm{d}t} = - V\text{\_nucleus} \cdot ([\text{nucleus\_Foxo1\_Pa0\_Pd0\_Pe0\_pUb0}] \cdot [\text{Akt\_P2}] \cdot V\text{\_cytoplasm} \cdot 0.0003)$$
$$- V\text{\_nucleus} \cdot ([\text{nucleus\_Foxo1\_Pa0\_Pd0\_Pe0\_pUb0}] \cdot [\text{SGK}] \cdot V\text{\_cytoplasm} \cdot 0.0003)$$
$$+ V\text{\_nucleus} \cdot ([\text{nucleus\_Foxo1\_Pa1\_Pd0\_Pe0\_pUb0}] \cdot [\text{PP2A}] \cdot V\text{\_cytoplasm} \cdot 1e-06)$$
$$- V\text{\_nucleus} \cdot ([\text{nucleus\_Foxo1\_Pa0\_Pd0\_Pe0\_pUb0}] \cdot [\text{IKK\_P}] \cdot V\text{\_cytoplasm} \cdot \text{by\_ikk\_phos\_factor} \cdot 5e-05)$$
$$+ V\text{\_nucleus} \cdot ([\text{nucleus\_Foxo1\_Pa0\_Pd1\_Pe0\_pUb0}] \cdot [\text{PP2A}] \cdot V\text{\_cytoplasm} \cdot 1e-06)$$
$$- V\text{\_nucleus} \cdot ([\text{nucleus\_Foxo1\_Pa0\_Pd0\_Pe0\_pUb0}] \cdot [\text{JNK\_P}] \cdot V\text{\_cytoplasm} \cdot \text{by\_jnk\_phos\_factor} \cdot 5e-05)$$
$$+ V\text{\_nucleus} \cdot ([\text{nucleus\_Foxo1\_Pa0\_Pd0\_Pe1\_pUb0}] \cdot [\text{PP2A}] \cdot V\text{\_cytoplasm} \cdot 1e-06)$$
$$- V\text{\_nucleus} \cdot ([\text{nucleus\_Foxo1\_Pa0\_Pd0\_Pe0\_pUb0}] \cdot [\text{SCF}] \cdot V\text{\_cytoplasm} \cdot 1e-06)$$
$$+ ([\text{cytoplasm\_Foxo1\_Pa0\_Pd0\_Pe0\_pUb0}] \cdot V\text{\_cytoplasm} \cdot 0.181818)$$
$$- ([\text{nucleus\_Foxo1\_Pa0\_Pd0\_Pe0\_pUb0}] \cdot V\text{\_nucleus} \cdot 0.055)$$
$$- ([\text{nucleus\_Foxo1\_Pa0\_Pd0\_Pe0\_pUb0}] \cdot V\text{\_nucleus} \cdot 0.25)$$
$$+ ([\text{dnabound\_Foxo1\_Pa0\_Pd0\_Pe0\_pUb0}] \cdot V\text{\_dnabound} \cdot 0.125)$$

$$\frac{\mathrm{d}\,([\text{nucleus\_Foxo1\_Pa0\_Pd0\_Pe0\_pUb1}] \cdot V\text{\_nucleus})}{\mathrm{d}t} = - V\text{\_nucleus} \cdot ([\text{nucleus\_Foxo1\_Pa0\_Pd0\_Pe0\_pUb1}] \cdot [\text{Akt\_P2}] \cdot V\text{\_cytoplasm} \cdot 0.0003)$$
$$- V\text{\_nucleus} \cdot ([\text{nucleus\_Foxo1\_Pa0\_Pd0\_Pe0\_pUb1}] \cdot [\text{SGK}] \cdot V\text{\_cytoplasm} \cdot 0.0003)$$
$$+ V\text{\_nucleus} \cdot ([\text{nucleus\_Foxo1\_Pa1\_Pd0\_Pe0\_pUb1}] \cdot [\text{PP2A}] \cdot V\text{\_cytoplasm} \cdot 1e-06)$$
$$- V\text{\_nucleus} \cdot ([\text{nucleus\_Foxo1\_Pa0\_Pd0\_Pe0\_pUb1}] \cdot [\text{IKK\_P}] \cdot V\text{\_cytoplasm} \cdot \text{by\_ikk\_phos\_factor} \cdot 5e-05)$$
$$+ V\text{\_nucleus} \cdot ([\text{nucleus\_Foxo1\_Pa0\_Pd1\_Pe0\_pUb1}] \cdot [\text{PP2A}] \cdot V\text{\_cytoplasm} \cdot 1e-06)$$
$$- V\text{\_nucleus} \cdot ([\text{nucleus\_Foxo1\_Pa0\_Pd0\_Pe0\_pUb1}] \cdot [\text{JNK\_P}] \cdot V\text{\_cytoplasm} \cdot \text{by\_jnk\_phos\_factor} \cdot 5e-05)$$
$$+ V\text{\_nucleus} \cdot ([\text{nucleus\_Foxo1\_Pa0\_Pd0\_Pe1\_pUb1}] \cdot [\text{PP2A}] \cdot V\text{\_cytoplasm} \cdot 1e-06)$$
$$+ V\text{\_nucleus} \cdot ([\text{nucleus\_Foxo1\_Pa0\_Pd0\_Pe0\_pUb0}] \cdot [\text{SCF}] \cdot V\text{\_cytoplasm} \cdot 1e-06)$$
$$+ ([\text{cytoplasm\_Foxo1\_Pa0\_Pd0\_Pe0\_pUb1}] \cdot V\text{\_cytoplasm} \cdot 0.181818)$$
$$- ([\text{nucleus\_Foxo1\_Pa0\_Pd0\_Pe0\_pUb1}] \cdot V\text{\_nucleus} \cdot 0.055)$$
$$- ([\text{nucleus\_Foxo1\_Pa0\_Pd0\_Pe0\_pUb1}] \cdot V\text{\_nucleus} \cdot 0.25)$$
$$+ ([\text{dnabound\_Foxo1\_Pa0\_Pd0\_Pe0\_pUb1}] \cdot V\text{\_dnabound} \cdot 0.125)$$

$$\frac{\mathrm{d}\,([\text{nucleus\_Foxo1\_Pa0\_Pd0\_Pe1\_pUb0}] \cdot V\text{\_nucleus})}{\mathrm{d}t} = - V\text{\_nucleus} \cdot ([\text{nucleus\_Foxo1\_Pa0\_Pd0\_Pe1\_pUb0}] \cdot [\text{Akt\_P2}] \cdot V\text{\_cytoplasm} \cdot 0.0003)$$
$$- V\text{\_nucleus} \cdot ([\text{nucleus\_Foxo1\_Pa0\_Pd0\_Pe1\_pUb0}] \cdot [\text{SGK}] \cdot V\text{\_cytoplasm} \cdot 0.0003)$$
$$+ V\text{\_nucleus} \cdot ([\text{nucleus\_Foxo1\_Pa1\_Pd0\_Pe1\_pUb0}] \cdot [\text{PP2A}] \cdot V\text{\_cytoplasm} \cdot 1e-06)$$
$$- V\text{\_nucleus} \cdot ([\text{nucleus\_Foxo1\_Pa0\_Pd0\_Pe1\_pUb0}] \cdot [\text{IKK\_P}] \cdot V\text{\_cytoplasm} \cdot \text{by\_ikk\_phos\_factor} \cdot 5e-05)$$
$$+ V\text{\_nucleus} \cdot ([\text{nucleus\_Foxo1\_Pa0\_Pd1\_Pe1\_pUb0}] \cdot [\text{PP2A}] \cdot V\text{\_cytoplasm} \cdot 1e-06)$$
$$+ V\text{\_nucleus} \cdot ([\text{nucleus\_Foxo1\_Pa0\_Pd0\_Pe0\_pUb0}] \cdot [\text{JNK\_P}] \cdot V\text{\_cytoplasm} \cdot \text{by\_jnk\_phos\_factor} \cdot 5e-05)$$
$$- V\text{\_nucleus} \cdot ([\text{nucleus\_Foxo1\_Pa0\_Pd0\_Pe1\_pUb0}] \cdot [\text{PP2A}] \cdot V\text{\_cytoplasm} \cdot 1e-06)$$
$$- V\text{\_nucleus} \cdot ([\text{nucleus\_Foxo1\_Pa0\_Pd0\_Pe1\_pUb0}] \cdot [\text{SCF}] \cdot V\text{\_cytoplasm} \cdot 1e-06)$$
$$+ ([\text{cytoplasm\_Foxo1\_Pa0\_Pd0\_Pe1\_pUb0}] \cdot V\text{\_cytoplasm} \cdot 1.81818)$$
$$- ([\text{nucleus\_Foxo1\_Pa0\_Pd0\_Pe1\_pUb0}] \cdot V\text{\_nucleus} \cdot 0.0055)$$
$$- ([\text{nucleus\_Foxo1\_Pa0\_Pd0\_Pe1\_pUb0}] \cdot V\text{\_nucleus} \cdot 0.25)$$
$$+ ([\text{dnabound\_Foxo1\_Pa0\_Pd0\_Pe1\_pUb0}] \cdot V\text{\_dnabound} \cdot 0.125)$$

$$\frac{\mathrm{d}\,([\text{nucleus\_Foxo1\_Pa0\_Pd0\_Pe1\_pUb1}] \cdot V\text{\_nucleus})}{\mathrm{d}t} = - V\text{\_nucleus} \cdot ([\text{nucleus\_Foxo1\_Pa0\_Pd0\_Pe1\_pUb1}] \cdot [\text{Akt\_P2}] \cdot V\text{\_cytoplasm} \cdot 0.0003)$$
$$- V\text{\_nucleus} \cdot ([\text{nucleus\_Foxo1\_Pa0\_Pd0\_Pe1\_pUb1}] \cdot [\text{SGK}] \cdot V\text{\_cytoplasm} \cdot 0.0003)$$
$$+ V\text{\_nucleus} \cdot ([\text{nucleus\_Foxo1\_Pa1\_Pd0\_Pe1\_pUb1}] \cdot [\text{PP2A}] \cdot V\text{\_cytoplasm} \cdot 1e-06)$$
$$- V\text{\_nucleus} \cdot ([\text{nucleus\_Foxo1\_Pa0\_Pd0\_Pe1\_pUb1}] \cdot [\text{IKK\_P}] \cdot V\text{\_cytoplasm} \cdot \text{by\_ikk\_phos\_factor} \cdot 5e-05)$$
$$+ V\text{\_nucleus} \cdot ([\text{nucleus\_Foxo1\_Pa0\_Pd1\_Pe1\_pUb1}] \cdot [\text{PP2A}] \cdot V\text{\_cytoplasm} \cdot 1e-06)$$
$$+ V\text{\_nucleus} \cdot ([\text{nucleus\_Foxo1\_Pa0\_Pd0\_Pe0\_pUb1}] \cdot [\text{JNK\_P}] \cdot V\text{\_cytoplasm} \cdot \text{by\_jnk\_phos\_factor} \cdot 5e-05)$$
$$- V\text{\_nucleus} \cdot ([\text{nucleus\_Foxo1\_Pa0\_Pd0\_Pe1\_pUb1}] \cdot [\text{PP2A}] \cdot V\text{\_cytoplasm} \cdot 1e-06)$$
$$+ V\text{\_nucleus} \cdot ([\text{nucleus\_Foxo1\_Pa0\_Pd0\_Pe1\_pUb0}] \cdot [\text{SCF}] \cdot V\text{\_cytoplasm} \cdot 1e-06)$$
$$+ ([\text{cytoplasm\_Foxo1\_Pa0\_Pd0\_Pe1\_pUb1}] \cdot V\text{\_cytoplasm} \cdot 1.81818)$$

$$- ([\text{nucleus\_Foxo1\_Pa0\_Pd0\_Pe1\_pUb1}] \cdot V\text{\_nucleus} \cdot 0.0055)$$
$$- ([\text{nucleus\_Foxo1\_Pa0\_Pd0\_Pe1\_pUb1}] \cdot V\text{\_nucleus} \cdot 0.25)$$
$$+ ([\text{dnabound\_Foxo1\_Pa0\_Pd0\_Pe1\_pUb1}] \cdot V\text{\_dnabound} \cdot 0.125)$$

$$\frac{\mathrm{d}\left([\text{nucleus\_Foxo1\_Pa0\_Pd1\_Pe0\_pUb0}] \cdot V\text{\_nucleus}\right)}{\mathrm{d}t} = - V\text{\_nucleus} \cdot ([\text{nucleus\_Foxo1\_Pa0\_Pd1\_Pe0\_pUb0}] \cdot [\text{Akt\_P2}] \cdot V\text{\_cytoplasm} \cdot 0.0003)$$
$$- V\text{\_nucleus} \cdot ([\text{nucleus\_Foxo1\_Pa0\_Pd1\_Pe0\_pUb0}] \cdot [\text{SGK}] \cdot V\text{\_cytoplasm} \cdot 0.0003)$$
$$+ V\text{\_nucleus} \cdot ([\text{nucleus\_Foxo1\_Pa1\_Pd1\_Pe0\_pUb0}] \cdot [\text{PP2A}] \cdot V\text{\_cytoplasm} \cdot 1e - 06)$$
$$+ V\text{\_nucleus} \cdot ([\text{nucleus\_Foxo1\_Pa0\_Pd0\_Pe0\_pUb0}] \cdot [\text{IKK\_P}] \cdot V\text{\_cytoplasm} \cdot \text{by\_ikk\_phos\_factor} \cdot 5e - 05)$$
$$- V\text{\_nucleus} \cdot ([\text{nucleus\_Foxo1\_Pa0\_Pd1\_Pe0\_pUb0}] \cdot [\text{PP2A}] \cdot V\text{\_cytoplasm} \cdot 1e - 06)$$
$$- V\text{\_nucleus} \cdot ([\text{nucleus\_Foxo1\_Pa0\_Pd1\_Pe0\_pUb0}] \cdot [\text{JNK\_P}] \cdot V\text{\_cytoplasm} \cdot \text{by\_jnk\_phos\_factor} \cdot 5e - 05)$$
$$+ V\text{\_nucleus} \cdot ([\text{nucleus\_Foxo1\_Pa0\_Pd1\_Pe1\_pUb0}] \cdot [\text{PP2A}] \cdot V\text{\_cytoplasm} \cdot 1e - 06)$$
$$- V\text{\_nucleus} \cdot ([\text{nucleus\_Foxo1\_Pa0\_Pd1\_Pe0\_pUb0}] \cdot [\text{SCF}] \cdot V\text{\_cytoplasm} \cdot 2.2e - 05)$$
$$+ ([\text{cytoplasm\_Foxo1\_Pa0\_Pd1\_Pe0\_pUb0}] \cdot V\text{\_cytoplasm} \cdot 0.0909091)$$
$$- ([\text{nucleus\_Foxo1\_Pa0\_Pd1\_Pe0\_pUb0}] \cdot V\text{\_nucleus} \cdot 0.55)$$
$$- ([\text{nucleus\_Foxo1\_Pa0\_Pd1\_Pe0\_pUb0}] \cdot V\text{\_nucleus} \cdot 0.25)$$
$$+ ([\text{dnabound\_Foxo1\_Pa0\_Pd1\_Pe0\_pUb0}] \cdot V\text{\_dnabound} \cdot 0.125)$$

$$\frac{\mathrm{d}\left([\text{nucleus\_Foxo1\_Pa0\_Pd1\_Pe0\_pUb1}] \cdot V\text{\_nucleus}\right)}{\mathrm{d}t} = - V\text{\_nucleus} \cdot ([\text{nucleus\_Foxo1\_Pa0\_Pd1\_Pe0\_pUb1}] \cdot [\text{Akt\_P2}] \cdot V\text{\_cytoplasm} \cdot 0.0003)$$
$$- V\text{\_nucleus} \cdot ([\text{nucleus\_Foxo1\_Pa0\_Pd1\_Pe0\_pUb1}] \cdot [\text{SGK}] \cdot V\text{\_cytoplasm} \cdot 0.0003)$$
$$+ V\text{\_nucleus} \cdot ([\text{nucleus\_Foxo1\_Pa1\_Pd1\_Pe0\_pUb1}] \cdot [\text{PP2A}] \cdot V\text{\_cytoplasm} \cdot 1e - 06)$$
$$+ V\text{\_nucleus} \cdot ([\text{nucleus\_Foxo1\_Pa0\_Pd0\_Pe0\_pUb1}] \cdot [\text{IKK\_P}] \cdot V\text{\_cytoplasm} \cdot \text{by\_ikk\_phos\_factor} \cdot 5e - 05)$$
$$- V\text{\_nucleus} \cdot ([\text{nucleus\_Foxo1\_Pa0\_Pd1\_Pe0\_pUb1}] \cdot [\text{PP2A}] \cdot V\text{\_cytoplasm} \cdot 1e - 06)$$
$$- V\text{\_nucleus} \cdot ([\text{nucleus\_Foxo1\_Pa0\_Pd1\_Pe0\_pUb1}] \cdot [\text{JNK\_P}] \cdot V\text{\_cytoplasm} \cdot \text{by\_jnk\_phos\_factor} \cdot 5e - 05)$$
$$+ V\text{\_nucleus} \cdot ([\text{nucleus\_Foxo1\_Pa0\_Pd1\_Pe1\_pUb1}] \cdot [\text{PP2A}] \cdot V\text{\_cytoplasm} \cdot 1e - 06)$$
$$+ V\text{\_nucleus} \cdot ([\text{nucleus\_Foxo1\_Pa0\_Pd1\_Pe0\_pUb0}] \cdot [\text{SCF}] \cdot V\text{\_cytoplasm} \cdot 2.2e - 05)$$
$$+ ([\text{cytoplasm\_Foxo1\_Pa0\_Pd1\_Pe0\_pUb1}] \cdot V\text{\_cytoplasm} \cdot 0.0909091)$$
$$- ([\text{nucleus\_Foxo1\_Pa0\_Pd1\_Pe0\_pUb1}] \cdot V\text{\_nucleus} \cdot 0.55)$$
$$- ([\text{nucleus\_Foxo1\_Pa0\_Pd1\_Pe0\_pUb1}] \cdot V\text{\_nucleus} \cdot 0.25)$$
$$+ ([\text{dnabound\_Foxo1\_Pa0\_Pd1\_Pe0\_pUb1}] \cdot V\text{\_dnabound} \cdot 0.125)$$

$$\frac{\mathrm{d}\left([\text{nucleus\_Foxo1\_Pa0\_Pd1\_Pe1\_pUb0}] \cdot V\text{\_nucleus}\right)}{\mathrm{d}t} = - V\text{\_nucleus} \cdot ([\text{nucleus\_Foxo1\_Pa0\_Pd1\_Pe1\_pUb0}] \cdot [\text{Akt\_P2}] \cdot V\text{\_cytoplasm} \cdot 0.0003)$$
$$- V\text{\_nucleus} \cdot ([\text{nucleus\_Foxo1\_Pa0\_Pd1\_Pe1\_pUb0}] \cdot [\text{SGK}] \cdot V\text{\_cytoplasm} \cdot 0.0003)$$
$$+ V\text{\_nucleus} \cdot ([\text{nucleus\_Foxo1\_Pa1\_Pd1\_Pe1\_pUb0}] \cdot [\text{PP2A}] \cdot V\text{\_cytoplasm} \cdot 1e - 06)$$
$$+ V\text{\_nucleus} \cdot ([\text{nucleus\_Foxo1\_Pa0\_Pd0\_Pe1\_pUb0}] \cdot [\text{IKK\_P}] \cdot V\text{\_cytoplasm} \cdot \text{by\_ikk\_phos\_factor} \cdot 5e - 05)$$
$$- V\text{\_nucleus} \cdot ([\text{nucleus\_Foxo1\_Pa0\_Pd1\_Pe1\_pUb0}] \cdot [\text{PP2A}] \cdot V\text{\_cytoplasm} \cdot 1e - 06)$$
$$+ V\text{\_nucleus} \cdot ([\text{nucleus\_Foxo1\_Pa0\_Pd1\_Pe0\_pUb0}] \cdot [\text{JNK\_P}] \cdot V\text{\_cytoplasm} \cdot \text{by\_jnk\_phos\_factor} \cdot 5e - 05)$$
$$- V\text{\_nucleus} \cdot ([\text{nucleus\_Foxo1\_Pa0\_Pd1\_Pe1\_pUb0}] \cdot [\text{PP2A}] \cdot V\text{\_cytoplasm} \cdot 1e - 06)$$
$$- V\text{\_nucleus} \cdot ([\text{nucleus\_Foxo1\_Pa0\_Pd1\_Pe1\_pUb0}] \cdot [\text{SCF}] \cdot V\text{\_cytoplasm} \cdot 2.2e - 05)$$
$$+ ([\text{cytoplasm\_Foxo1\_Pa0\_Pd1\_Pe1\_pUb0}] \cdot V\text{\_cytoplasm} \cdot 0.909091)$$
$$- ([\text{nucleus\_Foxo1\_Pa0\_Pd1\_Pe1\_pUb0}] \cdot V\text{\_nucleus} \cdot 0.055)$$
$$- ([\text{nucleus\_Foxo1\_Pa0\_Pd1\_Pe1\_pUb0}] \cdot V\text{\_nucleus} \cdot 0.25)$$
$$+ ([\text{dnabound\_Foxo1\_Pa0\_Pd1\_Pe1\_pUb0}] \cdot V\text{\_dnabound} \cdot 0.125)$$

$$\frac{\mathrm{d}\left([\text{nucleus\_Foxo1\_Pa0\_Pd1\_Pe1\_pUb1}] \cdot V\text{\_nucleus}\right)}{\mathrm{d}t} = - V\text{\_nucleus} \cdot ([\text{nucleus\_Foxo1\_Pa0\_Pd1\_Pe1\_pUb1}] \cdot [\text{Akt\_P2}] \cdot V\text{\_cytoplasm} \cdot 0.0003)$$
$$- V\text{\_nucleus} \cdot ([\text{nucleus\_Foxo1\_Pa0\_Pd1\_Pe1\_pUb1}] \cdot [\text{SGK}] \cdot V\text{\_cytoplasm} \cdot 0.0003)$$
$$+ V\text{\_nucleus} \cdot ([\text{nucleus\_Foxo1\_Pa1\_Pd1\_Pe1\_pUb1}] \cdot [\text{PP2A}] \cdot V\text{\_cytoplasm} \cdot 1e - 06)$$
$$+ V\text{\_nucleus} \cdot ([\text{nucleus\_Foxo1\_Pa0\_Pd0\_Pe1\_pUb1}] \cdot [\text{IKK\_P}] \cdot V\text{\_cytoplasm} \cdot \text{by\_ikk\_phos\_factor} \cdot 5e - 05)$$
$$- V\text{\_nucleus} \cdot ([\text{nucleus\_Foxo1\_Pa0\_Pd1\_Pe1\_pUb1}] \cdot [\text{PP2A}] \cdot V\text{\_cytoplasm} \cdot 1e - 06)$$
$$+ V\text{\_nucleus} \cdot ([\text{nucleus\_Foxo1\_Pa0\_Pd1\_Pe0\_pUb1}] \cdot [\text{JNK\_P}] \cdot V\text{\_cytoplasm} \cdot \text{by\_jnk\_phos\_factor} \cdot 5e - 05)$$
$$- V\text{\_nucleus} \cdot ([\text{nucleus\_Foxo1\_Pa0\_Pd1\_Pe1\_pUb1}] \cdot [\text{PP2A}] \cdot V\text{\_cytoplasm} \cdot 1e - 06)$$
$$+ V\text{\_nucleus} \cdot ([\text{nucleus\_Foxo1\_Pa0\_Pd1\_Pe1\_pUb0}] \cdot [\text{SCF}] \cdot V\text{\_cytoplasm} \cdot 2.2e - 05)$$
$$+ ([\text{cytoplasm\_Foxo1\_Pa0\_Pd1\_Pe1\_pUb1}] \cdot V\text{\_cytoplasm} \cdot 0.909091)$$
$$- ([\text{nucleus\_Foxo1\_Pa0\_Pd1\_Pe1\_pUb1}] \cdot V\text{\_nucleus} \cdot 0.055)$$
$$- ([\text{nucleus\_Foxo1\_Pa0\_Pd1\_Pe1\_pUb1}] \cdot V\text{\_nucleus} \cdot 0.25)$$
$$+ ([\text{dnabound\_Foxo1\_Pa0\_Pd1\_Pe1\_pUb1}] \cdot V\text{\_dnabound} \cdot 0.125)$$

$$\frac{\mathrm{d}\left([\text{nucleus\_Foxo1\_Pa1\_Pd0\_Pe0\_pUb0}] \cdot V\text{\_nucleus}\right)}{\mathrm{d}t} = + V\text{\_nucleus} \cdot ([\text{nucleus\_Foxo1\_Pa0\_Pd0\_Pe0\_pUb0}] \cdot [\text{Akt\_P2}] \cdot V\text{\_cytoplasm} \cdot 0.0003)$$
$$+ V\text{\_nucleus} \cdot ([\text{nucleus\_Foxo1\_Pa0\_Pd0\_Pe0\_pUb0}] \cdot [\text{SGK}] \cdot V\text{\_cytoplasm} \cdot 0.0003)$$
$$- V\text{\_nucleus} \cdot ([\text{nucleus\_Foxo1\_Pa1\_Pd0\_Pe0\_pUb0}] \cdot [\text{PP2A}] \cdot V\text{\_cytoplasm} \cdot 1e - 06)$$
$$- V\text{\_nucleus} \cdot ([\text{nucleus\_Foxo1\_Pa1\_Pd0\_Pe0\_pUb0}] \cdot [\text{IKK\_P}] \cdot V\text{\_cytoplasm} \cdot \text{by\_ikk\_phos\_factor} \cdot 5e - 05)$$
$$+ V\text{\_nucleus} \cdot ([\text{nucleus\_Foxo1\_Pa1\_Pd1\_Pe0\_pUb0}] \cdot [\text{PP2A}] \cdot V\text{\_cytoplasm} \cdot 1e - 06)$$
$$- V\text{\_nucleus} \cdot ([\text{nucleus\_Foxo1\_Pa1\_Pd0\_Pe0\_pUb0}] \cdot [\text{JNK\_P}] \cdot V\text{\_cytoplasm} \cdot \text{by\_jnk\_phos\_factor} \cdot 5e - 05)$$
$$+ V\text{\_nucleus} \cdot ([\text{nucleus\_Foxo1\_Pa1\_Pd0\_Pe1\_pUb0}] \cdot [\text{PP2A}] \cdot V\text{\_cytoplasm} \cdot 1e - 06)$$
$$- V\text{\_nucleus} \cdot ([\text{nucleus\_Foxo1\_Pa1\_Pd0\_Pe0\_pUb0}] \cdot [\text{SCF}] \cdot V\text{\_cytoplasm} \cdot 3e - 06)$$
$$+ ([\text{cytoplasm\_Foxo1\_Pa1\_Pd0\_Pe0\_pUb0}] \cdot V\text{\_cytoplasm} \cdot 0.0181818)$$
$$- ([\text{nucleus\_Foxo1\_Pa1\_Pd0\_Pe0\_pUb0}] \cdot V\text{\_nucleus} \cdot 0.55)$$
$$- ([\text{nucleus\_Foxo1\_Pa1\_Pd0\_Pe0\_pUb0}] \cdot V\text{\_nucleus} \cdot 0.125)$$
$$+ ([\text{dnabound\_Foxo1\_Pa1\_Pd0\_Pe0\_pUb0}] \cdot V\text{\_dnabound} \cdot 0.125)$$

$$\frac{\mathrm{d}\left([\text{nucleus\_Foxo1\_Pa1\_Pd0\_Pe0\_pUb1}] \cdot V\text{\_nucleus}\right)}{\mathrm{d}t} = + ([\text{dnabound\_Foxo1\_Pa1\_Pd0\_Pe0\_pUb1}] \cdot V\text{\_dnabound} \cdot 0.125)$$
$$+ V\text{\_nucleus} \cdot ([\text{nucleus\_Foxo1\_Pa0\_Pd0\_Pe0\_pUb1}] \cdot [\text{Akt\_P2}] \cdot V\text{\_cytoplasm} \cdot 0.0003)$$
$$+ V\text{\_nucleus} \cdot ([\text{nucleus\_Foxo1\_Pa0\_Pd0\_Pe0\_pUb1}] \cdot [\text{SGK}] \cdot V\text{\_cytoplasm} \cdot 0.0003)$$
$$- V\text{\_nucleus} \cdot ([\text{nucleus\_Foxo1\_Pa1\_Pd0\_Pe0\_pUb1}] \cdot [\text{PP2A}] \cdot V\text{\_cytoplasm} \cdot 1e - 06)$$
$$- V\text{\_nucleus} \cdot ([\text{nucleus\_Foxo1\_Pa1\_Pd0\_Pe0\_pUb1}] \cdot [\text{IKK\_P}] \cdot V\text{\_cytoplasm} \cdot \text{by\_ikk\_phos\_factor} \cdot 5e - 05)$$
$$+ V\text{\_nucleus} \cdot ([\text{nucleus\_Foxo1\_Pa1\_Pd1\_Pe0\_pUb1}] \cdot [\text{PP2A}] \cdot V\text{\_cytoplasm} \cdot 1e - 06)$$
$$- V\text{\_nucleus} \cdot ([\text{nucleus\_Foxo1\_Pa1\_Pd0\_Pe0\_pUb1}] \cdot [\text{JNK\_P}] \cdot V\text{\_cytoplasm} \cdot \text{by\_jnk\_phos\_factor} \cdot 5e - 05)$$
$$+ V\text{\_nucleus} \cdot ([\text{nucleus\_Foxo1\_Pa1\_Pd0\_Pe1\_pUb1}] \cdot [\text{PP2A}] \cdot V\text{\_cytoplasm} \cdot 1e - 06)$$
$$+ V\text{\_nucleus} \cdot ([\text{nucleus\_Foxo1\_Pa1\_Pd0\_Pe0\_pUb0}] \cdot [\text{SCF}] \cdot V\text{\_cytoplasm} \cdot 3e - 06)$$
$$+ ([\text{cytoplasm\_Foxo1\_Pa1\_Pd0\_Pe0\_pUb1}] \cdot V\text{\_cytoplasm} \cdot 0.0181818)$$
$$- ([\text{nucleus\_Foxo1\_Pa1\_Pd0\_Pe0\_pUb1}] \cdot V\text{\_nucleus} \cdot 0.55)$$
$$- ([\text{nucleus\_Foxo1\_Pa1\_Pd0\_Pe0\_pUb1}] \cdot V\text{\_nucleus} \cdot 0.125)$$

$$\frac{\mathrm{d}\left([\text{nucleus\_Foxo1\_Pa1\_Pd0\_Pe1\_pUb0}] \cdot V\text{\_nucleus}\right)}{\mathrm{d}t} = + ([\text{cytoplasm\_Foxo1\_Pa1\_Pd0\_Pe1\_pUb0}] \cdot V\text{\_cytoplasm} \cdot 0.181818)$$
$$- ([\text{nucleus\_Foxo1\_Pa1\_Pd0\_Pe1\_pUb0}] \cdot V\text{\_nucleus} \cdot 0.055)$$

$$
\begin{aligned}
&- ([\mathrm{nucleus\_Foxo1\_Pa1\_Pd0\_Pe1\_pUb0}] \cdot V\_\mathrm{nucleus} \cdot 0.125) \\
&+ ([\mathrm{dnabound\_Foxo1\_Pa1\_Pd0\_Pe1\_pUb0}] \cdot V\_\mathrm{dnabound} \cdot 0.125) \\
&+ V\_\mathrm{nucleus} \cdot ([\mathrm{nucleus\_Foxo1\_Pa0\_Pd0\_Pe1\_pUb0}] \cdot [\mathrm{Akt\_P2}] \cdot V\_\mathrm{cytoplasm} \cdot 0.0003) \\
&+ V\_\mathrm{nucleus} \cdot ([\mathrm{nucleus\_Foxo1\_Pa0\_Pd0\_Pe1\_pUb0}] \cdot [\mathrm{SGK}] \cdot V\_\mathrm{cytoplasm} \cdot 0.0003) \\
&- V\_\mathrm{nucleus} \cdot ([\mathrm{nucleus\_Foxo1\_Pa1\_Pd0\_Pe1\_pUb0}] \cdot [\mathrm{PP2A}] \cdot V\_\mathrm{cytoplasm} \cdot 1e-06) \\
&- V\_\mathrm{nucleus} \cdot ([\mathrm{nucleus\_Foxo1\_Pa1\_Pd0\_Pe1\_pUb0}] \cdot [\mathrm{IKK\_P}] \cdot V\_\mathrm{cytoplasm} \cdot \mathrm{by\_ikk\_phos\_factor} \cdot 5e-05) \\
&+ V\_\mathrm{nucleus} \cdot ([\mathrm{nucleus\_Foxo1\_Pa1\_Pd1\_Pe1\_pUb0}] \cdot [\mathrm{PP2A}] \cdot V\_\mathrm{cytoplasm} \cdot 1e-06) \\
&+ V\_\mathrm{nucleus} \cdot ([\mathrm{nucleus\_Foxo1\_Pa1\_Pd0\_Pe0\_pUb0}] \cdot [\mathrm{JNK\_P}] \cdot V\_\mathrm{cytoplasm} \cdot \mathrm{by\_jnk\_phos\_factor} \cdot 5e-05) \\
&- V\_\mathrm{nucleus} \cdot ([\mathrm{nucleus\_Foxo1\_Pa1\_Pd0\_Pe1\_pUb0}] \cdot [\mathrm{PP2A}] \cdot V\_\mathrm{cytoplasm} \cdot 1e-06) \\
&- V\_\mathrm{nucleus} \cdot ([\mathrm{nucleus\_Foxo1\_Pa1\_Pd0\_Pe1\_pUb0}] \cdot [\mathrm{SCF}] \cdot V\_\mathrm{cytoplasm} \cdot 3e-06)
\end{aligned}
$$

$$
\begin{aligned}
\frac{\mathrm{d}([\mathrm{nucleus\_Foxo1\_Pa1\_Pd0\_Pe1\_pUb1}] \cdot V\_\mathrm{nucleus})}{\mathrm{d}t} = &+ ([\mathrm{cytoplasm\_Foxo1\_Pa1\_Pd0\_Pe1\_pUb1}] \cdot V\_\mathrm{cytoplasm} \cdot 0.181818) \\
&- ([\mathrm{nucleus\_Foxo1\_Pa1\_Pd0\_Pe1\_pUb1}] \cdot V\_\mathrm{nucleus} \cdot 0.055) \\
&- ([\mathrm{nucleus\_Foxo1\_Pa1\_Pd0\_Pe1\_pUb1}] \cdot V\_\mathrm{nucleus} \cdot 0.125) \\
&+ ([\mathrm{dnabound\_Foxo1\_Pa1\_Pd0\_Pe1\_pUb1}] \cdot V\_\mathrm{dnabound} \cdot 0.125) \\
&+ V\_\mathrm{nucleus} \cdot ([\mathrm{nucleus\_Foxo1\_Pa0\_Pd0\_Pe1\_pUb1}] \cdot [\mathrm{Akt\_P2}] \cdot V\_\mathrm{cytoplasm} \cdot 0.0003) \\
&+ V\_\mathrm{nucleus} \cdot ([\mathrm{nucleus\_Foxo1\_Pa0\_Pd0\_Pe1\_pUb1}] \cdot [\mathrm{SGK}] \cdot V\_\mathrm{cytoplasm} \cdot 0.0003) \\
&- V\_\mathrm{nucleus} \cdot ([\mathrm{nucleus\_Foxo1\_Pa1\_Pd0\_Pe1\_pUb1}] \cdot [\mathrm{PP2A}] \cdot V\_\mathrm{cytoplasm} \cdot 1e-06) \\
&- V\_\mathrm{nucleus} \cdot ([\mathrm{nucleus\_Foxo1\_Pa1\_Pd0\_Pe1\_pUb1}] \cdot [\mathrm{IKK\_P}] \cdot V\_\mathrm{cytoplasm} \cdot \mathrm{by\_ikk\_phos\_factor} \cdot 5e-05) \\
&+ V\_\mathrm{nucleus} \cdot ([\mathrm{nucleus\_Foxo1\_Pa1\_Pd1\_Pe1\_pUb1}] \cdot [\mathrm{PP2A}] \cdot V\_\mathrm{cytoplasm} \cdot 1e-06) \\
&+ V\_\mathrm{nucleus} \cdot ([\mathrm{nucleus\_Foxo1\_Pa1\_Pd0\_Pe0\_pUb1}] \cdot [\mathrm{JNK\_P}] \cdot V\_\mathrm{cytoplasm} \cdot \mathrm{by\_jnk\_phos\_factor} \cdot 5e-05) \\
&- V\_\mathrm{nucleus} \cdot ([\mathrm{nucleus\_Foxo1\_Pa1\_Pd0\_Pe1\_pUb1}] \cdot [\mathrm{PP2A}] \cdot V\_\mathrm{cytoplasm} \cdot 1e-06) \\
&+ V\_\mathrm{nucleus} \cdot ([\mathrm{nucleus\_Foxo1\_Pa1\_Pd0\_Pe1\_pUb0}] \cdot [\mathrm{SCF}] \cdot V\_\mathrm{cytoplasm} \cdot 3e-06)
\end{aligned}
$$

$$
\begin{aligned}
\frac{\mathrm{d}([\mathrm{nucleus\_Foxo1\_Pa1\_Pd1\_Pe0\_pUb0}] \cdot V\_\mathrm{nucleus})}{\mathrm{d}t} = &+ ([\mathrm{cytoplasm\_Foxo1\_Pa1\_Pd1\_Pe0\_pUb0}] \cdot V\_\mathrm{cytoplasm} \cdot 0.00909091) \\
&- ([\mathrm{nucleus\_Foxo1\_Pa1\_Pd1\_Pe0\_pUb0}] \cdot V\_\mathrm{nucleus} \cdot 5.5) \\
&- ([\mathrm{nucleus\_Foxo1\_Pa1\_Pd1\_Pe0\_pUb0}] \cdot V\_\mathrm{nucleus} \cdot 0.125) \\
&+ ([\mathrm{dnabound\_Foxo1\_Pa1\_Pd1\_Pe0\_pUb0}] \cdot V\_\mathrm{dnabound} \cdot 0.125) \\
&+ V\_\mathrm{nucleus} \cdot ([\mathrm{nucleus\_Foxo1\_Pa0\_Pd1\_Pe0\_pUb0}] \cdot [\mathrm{Akt\_P2}] \cdot V\_\mathrm{cytoplasm} \cdot 0.0003) \\
&+ V\_\mathrm{nucleus} \cdot ([\mathrm{nucleus\_Foxo1\_Pa0\_Pd1\_Pe0\_pUb0}] \cdot [\mathrm{SGK}] \cdot V\_\mathrm{cytoplasm} \cdot 0.0003) \\
&- V\_\mathrm{nucleus} \cdot ([\mathrm{nucleus\_Foxo1\_Pa1\_Pd1\_Pe0\_pUb0}] \cdot [\mathrm{PP2A}] \cdot V\_\mathrm{cytoplasm} \cdot 1e-06) \\
&+ V\_\mathrm{nucleus} \cdot ([\mathrm{nucleus\_Foxo1\_Pa1\_Pd0\_Pe0\_pUb0}] \cdot [\mathrm{IKK\_P}] \cdot V\_\mathrm{cytoplasm} \cdot \mathrm{by\_ikk\_phos\_factor} \cdot 5e-05) \\
&- V\_\mathrm{nucleus} \cdot ([\mathrm{nucleus\_Foxo1\_Pa1\_Pd1\_Pe0\_pUb0}] \cdot [\mathrm{PP2A}] \cdot V\_\mathrm{cytoplasm} \cdot 1e-06) \\
&- V\_\mathrm{nucleus} \cdot ([\mathrm{nucleus\_Foxo1\_Pa1\_Pd1\_Pe0\_pUb0}] \cdot [\mathrm{JNK\_P}] \cdot V\_\mathrm{cytoplasm} \cdot \mathrm{by\_jnk\_phos\_factor} \cdot 5e-05) \\
&+ V\_\mathrm{nucleus} \cdot ([\mathrm{nucleus\_Foxo1\_Pa1\_Pd1\_Pe1\_pUb0}] \cdot [\mathrm{PP2A}] \cdot V\_\mathrm{cytoplasm} \cdot 1e-06) \\
&- V\_\mathrm{nucleus} \cdot ([\mathrm{nucleus\_Foxo1\_Pa1\_Pd1\_Pe0\_pUb0}] \cdot [\mathrm{SCF}] \cdot V\_\mathrm{cytoplasm} \cdot 6.6e-05)
\end{aligned}
$$

$$
\begin{aligned}
\frac{\mathrm{d}([\mathrm{nucleus\_Foxo1\_Pa1\_Pd1\_Pe0\_pUb1}] \cdot V\_\mathrm{nucleus})}{\mathrm{d}t} = &+ ([\mathrm{cytoplasm\_Foxo1\_Pa1\_Pd1\_Pe0\_pUb1}] \cdot V\_\mathrm{cytoplasm} \cdot 0.00909091) \\
&- ([\mathrm{nucleus\_Foxo1\_Pa1\_Pd1\_Pe0\_pUb1}] \cdot V\_\mathrm{nucleus} \cdot 5.5) \\
&- ([\mathrm{nucleus\_Foxo1\_Pa1\_Pd1\_Pe0\_pUb1}] \cdot V\_\mathrm{nucleus} \cdot 0.125) \\
&+ ([\mathrm{dnabound\_Foxo1\_Pa1\_Pd1\_Pe0\_pUb1}] \cdot V\_\mathrm{dnabound} \cdot 0.125) \\
&+ V\_\mathrm{nucleus} \cdot ([\mathrm{nucleus\_Foxo1\_Pa0\_Pd1\_Pe0\_pUb1}] \cdot [\mathrm{Akt\_P2}] \cdot V\_\mathrm{cytoplasm} \cdot 0.0003) \\
&+ V\_\mathrm{nucleus} \cdot ([\mathrm{nucleus\_Foxo1\_Pa0\_Pd1\_Pe0\_pUb1}] \cdot [\mathrm{SGK}] \cdot V\_\mathrm{cytoplasm} \cdot 0.0003) \\
&- V\_\mathrm{nucleus} \cdot ([\mathrm{nucleus\_Foxo1\_Pa1\_Pd1\_Pe0\_pUb1}] \cdot [\mathrm{PP2A}] \cdot V\_\mathrm{cytoplasm} \cdot 1e-06) \\
&+ V\_\mathrm{nucleus} \cdot ([\mathrm{nucleus\_Foxo1\_Pa1\_Pd0\_Pe0\_pUb1}] \cdot [\mathrm{IKK\_P}] \cdot V\_\mathrm{cytoplasm} \cdot \mathrm{by\_ikk\_phos\_factor} \cdot 5e-05) \\
&- V\_\mathrm{nucleus} \cdot ([\mathrm{nucleus\_Foxo1\_Pa1\_Pd1\_Pe0\_pUb1}] \cdot [\mathrm{PP2A}] \cdot V\_\mathrm{cytoplasm} \cdot 1e-06) \\
&- V\_\mathrm{nucleus} \cdot ([\mathrm{nucleus\_Foxo1\_Pa1\_Pd1\_Pe0\_pUb1}] \cdot [\mathrm{JNK\_P}] \cdot V\_\mathrm{cytoplasm} \cdot \mathrm{by\_jnk\_phos\_factor} \cdot 5e-05) \\
&+ V\_\mathrm{nucleus} \cdot ([\mathrm{nucleus\_Foxo1\_Pa1\_Pd1\_Pe1\_pUb1}] \cdot [\mathrm{PP2A}] \cdot V\_\mathrm{cytoplasm} \cdot 1e-06) \\
&+ V\_\mathrm{nucleus} \cdot ([\mathrm{nucleus\_Foxo1\_Pa1\_Pd1\_Pe0\_pUb0}] \cdot [\mathrm{SCF}] \cdot V\_\mathrm{cytoplasm} \cdot 6.6e-05)
\end{aligned}
$$

$$
\begin{aligned}
\frac{\mathrm{d}([\mathrm{nucleus\_Foxo1\_Pa1\_Pd1\_Pe1\_pUb0}] \cdot V\_\mathrm{nucleus})}{\mathrm{d}t} = &+ ([\mathrm{cytoplasm\_Foxo1\_Pa1\_Pd1\_Pe1\_pUb0}] \cdot V\_\mathrm{cytoplasm} \cdot 0.0909091) \\
&- ([\mathrm{nucleus\_Foxo1\_Pa1\_Pd1\_Pe1\_pUb0}] \cdot V\_\mathrm{nucleus} \cdot 0.55) \\
&- ([\mathrm{nucleus\_Foxo1\_Pa1\_Pd1\_Pe1\_pUb0}] \cdot V\_\mathrm{nucleus} \cdot 0.125) \\
&+ ([\mathrm{dnabound\_Foxo1\_Pa1\_Pd1\_Pe1\_pUb0}] \cdot V\_\mathrm{dnabound} \cdot 0.125) \\
&+ V\_\mathrm{nucleus} \cdot ([\mathrm{nucleus\_Foxo1\_Pa0\_Pd1\_Pe1\_pUb0}] \cdot [\mathrm{Akt\_P2}] \cdot V\_\mathrm{cytoplasm} \cdot 0.0003) \\
&+ V\_\mathrm{nucleus} \cdot ([\mathrm{nucleus\_Foxo1\_Pa0\_Pd1\_Pe1\_pUb0}] \cdot [\mathrm{SGK}] \cdot V\_\mathrm{cytoplasm} \cdot 0.0003) \\
&- V\_\mathrm{nucleus} \cdot ([\mathrm{nucleus\_Foxo1\_Pa1\_Pd1\_Pe1\_pUb0}] \cdot [\mathrm{PP2A}] \cdot V\_\mathrm{cytoplasm} \cdot 1e-06) \\
&+ V\_\mathrm{nucleus} \cdot ([\mathrm{nucleus\_Foxo1\_Pa1\_Pd0\_Pe1\_pUb0}] \cdot [\mathrm{IKK\_P}] \cdot V\_\mathrm{cytoplasm} \cdot \mathrm{by\_ikk\_phos\_factor} \cdot 5e-05) \\
&- V\_\mathrm{nucleus} \cdot ([\mathrm{nucleus\_Foxo1\_Pa1\_Pd1\_Pe1\_pUb0}] \cdot [\mathrm{PP2A}] \cdot V\_\mathrm{cytoplasm} \cdot 1e-06) \\
&+ V\_\mathrm{nucleus} \cdot ([\mathrm{nucleus\_Foxo1\_Pa1\_Pd1\_Pe0\_pUb0}] \cdot [\mathrm{JNK\_P}] \cdot V\_\mathrm{cytoplasm} \cdot \mathrm{by\_jnk\_phos\_factor} \cdot 5e-05) \\
&- V\_\mathrm{nucleus} \cdot ([\mathrm{nucleus\_Foxo1\_Pa1\_Pd1\_Pe1\_pUb0}] \cdot [\mathrm{PP2A}] \cdot V\_\mathrm{cytoplasm} \cdot 1e-06) \\
&- V\_\mathrm{nucleus} \cdot ([\mathrm{nucleus\_Foxo1\_Pa1\_Pd1\_Pe1\_pUb0}] \cdot [\mathrm{SCF}] \cdot V\_\mathrm{cytoplasm} \cdot 6.6e-05)
\end{aligned}
$$

$$
\begin{aligned}
\frac{\mathrm{d}([\mathrm{nucleus\_Foxo1\_Pa1\_Pd1\_Pe1\_pUb1}] \cdot V\_\mathrm{nucleus})}{\mathrm{d}t} = &+ ([\mathrm{cytoplasm\_Foxo1\_Pa1\_Pd1\_Pe1\_pUb1}] \cdot V\_\mathrm{cytoplasm} \cdot 0.0909091) \\
&- ([\mathrm{nucleus\_Foxo1\_Pa1\_Pd1\_Pe1\_pUb1}] \cdot V\_\mathrm{nucleus} \cdot 0.55) \\
&- ([\mathrm{nucleus\_Foxo1\_Pa1\_Pd1\_Pe1\_pUb1}] \cdot V\_\mathrm{nucleus} \cdot 0.125) \\
&+ ([\mathrm{dnabound\_Foxo1\_Pa1\_Pd1\_Pe1\_pUb1}] \cdot V\_\mathrm{dnabound} \cdot 0.125) \\
&+ V\_\mathrm{nucleus} \cdot ([\mathrm{nucleus\_Foxo1\_Pa0\_Pd1\_Pe1\_pUb1}] \cdot [\mathrm{Akt\_P2}] \cdot V\_\mathrm{cytoplasm} \cdot 0.0003) \\
&+ V\_\mathrm{nucleus} \cdot ([\mathrm{nucleus\_Foxo1\_Pa0\_Pd1\_Pe1\_pUb1}] \cdot [\mathrm{SGK}] \cdot V\_\mathrm{cytoplasm} \cdot 0.0003) \\
&- V\_\mathrm{nucleus} \cdot ([\mathrm{nucleus\_Foxo1\_Pa1\_Pd1\_Pe1\_pUb1}] \cdot [\mathrm{PP2A}] \cdot V\_\mathrm{cytoplasm} \cdot 1e-06) \\
&+ V\_\mathrm{nucleus} \cdot ([\mathrm{nucleus\_Foxo1\_Pa1\_Pd0\_Pe1\_pUb1}] \cdot [\mathrm{IKK\_P}] \cdot V\_\mathrm{cytoplasm} \cdot \mathrm{by\_ikk\_phos\_factor} \cdot 5e-05) \\
&- V\_\mathrm{nucleus} \cdot ([\mathrm{nucleus\_Foxo1\_Pa1\_Pd1\_Pe1\_pUb1}] \cdot [\mathrm{PP2A}] \cdot V\_\mathrm{cytoplasm} \cdot 1e-06) \\
&+ V\_\mathrm{nucleus} \cdot ([\mathrm{nucleus\_Foxo1\_Pa1\_Pd1\_Pe0\_pUb1}] \cdot [\mathrm{JNK\_P}] \cdot V\_\mathrm{cytoplasm} \cdot \mathrm{by\_jnk\_phos\_factor} \cdot 5e-05) \\
&- V\_\mathrm{nucleus} \cdot ([\mathrm{nucleus\_Foxo1\_Pa1\_Pd1\_Pe1\_pUb1}] \cdot [\mathrm{PP2A}] \cdot V\_\mathrm{cytoplasm} \cdot 1e-06) \\
&+ V\_\mathrm{nucleus} \cdot ([\mathrm{nucleus\_Foxo1\_Pa1\_Pd1\_Pe1\_pUb0}] \cdot [\mathrm{SCF}] \cdot V\_\mathrm{cytoplasm} \cdot 6.6e-05)
\end{aligned}
$$

$$
\begin{aligned}
\frac{\mathrm{d}([\mathrm{nucleus\_RNA\_InR}] \cdot V\_\mathrm{nucleus})}{\mathrm{d}t} = &+ ([\mathrm{dnabound\_Foxo1\_Pa0\_Pd0\_Pe0\_pUb0}] \cdot V\_\mathrm{dnabound} \cdot 0.24) \\
&+ ([\mathrm{dnabound\_Foxo1\_Pa0\_Pd0\_Pe0\_pUb1}] \cdot V\_\mathrm{dnabound} \cdot 0.24) \\
&+ ([\mathrm{dnabound\_Foxo1\_Pa0\_Pd0\_Pe1\_pUb0}] \cdot V\_\mathrm{dnabound} \cdot 0.24) \\
&+ ([\mathrm{dnabound\_Foxo1\_Pa0\_Pd0\_Pe1\_pUb1}] \cdot V\_\mathrm{dnabound} \cdot 0.24) \\
&+ ([\mathrm{dnabound\_Foxo1\_Pa0\_Pd1\_Pe0\_pUb0}] \cdot V\_\mathrm{dnabound} \cdot 0.24) \\
&+ ([\mathrm{dnabound\_Foxo1\_Pa0\_Pd1\_Pe0\_pUb1}] \cdot V\_\mathrm{dnabound} \cdot 0.24) \\
&+ ([\mathrm{dnabound\_Foxo1\_Pa0\_Pd1\_Pe1\_pUb0}] \cdot V\_\mathrm{dnabound} \cdot 0.24)
\end{aligned}
$$

$$
\begin{aligned}
&+ (\text{[dnabound\_Foxo1\_Pa0\_Pd1\_Pe1\_pUb1]} \cdot V\text{\_dnabound} \cdot 0.24)\\
&+ (\text{[dnabound\_Foxo1\_Pa1\_Pd0\_Pe0\_pUb0]} \cdot V\text{\_dnabound} \cdot 0.24)\\
&+ (\text{[dnabound\_Foxo1\_Pa1\_Pd0\_Pe0\_pUb1]} \cdot V\text{\_dnabound} \cdot 0.24)\\
&+ (\text{[dnabound\_Foxo1\_Pa1\_Pd0\_Pe1\_pUb0]} \cdot V\text{\_dnabound} \cdot 0.24)\\
&+ (\text{[dnabound\_Foxo1\_Pa1\_Pd0\_Pe1\_pUb1]} \cdot V\text{\_dnabound} \cdot 0.24)\\
&+ (\text{[dnabound\_Foxo1\_Pa1\_Pd1\_Pe0\_pUb0]} \cdot V\text{\_dnabound} \cdot 0.24)\\
&+ (\text{[dnabound\_Foxo1\_Pa1\_Pd1\_Pe0\_pUb1]} \cdot V\text{\_dnabound} \cdot 0.24)\\
&+ (\text{[dnabound\_Foxo1\_Pa1\_Pd1\_Pe1\_pUb0]} \cdot V\text{\_dnabound} \cdot 0.24)\\
&+ (\text{[dnabound\_Foxo1\_Pa1\_Pd1\_Pe1\_pUb1]} \cdot V\text{\_dnabound} \cdot 0.24)\\
&+ (5)\\
&- (\text{[nucleus\_RNA\_InR]} \cdot V\text{\_nucleus} \cdot 0.22)
\end{aligned}
$$

$$
\begin{aligned}
\frac{\mathrm{d}\left(\text{[nucleus\_RNA\_SOD2]} \cdot V\text{\_nucleus}\right)}{\mathrm{d}t} =\; &+ (\text{[dnabound\_Foxo1\_Pa0\_Pd0\_Pe0\_pUb0]} \cdot V\text{\_dnabound} \cdot 0.95)\\
&+ (\text{[dnabound\_Foxo1\_Pa0\_Pd0\_Pe0\_pUb1]} \cdot V\text{\_dnabound} \cdot 0.95)\\
&+ (\text{[dnabound\_Foxo1\_Pa0\_Pd0\_Pe1\_pUb0]} \cdot V\text{\_dnabound} \cdot 0.95)\\
&+ (\text{[dnabound\_Foxo1\_Pa0\_Pd0\_Pe1\_pUb1]} \cdot V\text{\_dnabound} \cdot 0.95)\\
&+ (\text{[dnabound\_Foxo1\_Pa0\_Pd1\_Pe0\_pUb0]} \cdot V\text{\_dnabound} \cdot 0.95)\\
&+ (\text{[dnabound\_Foxo1\_Pa0\_Pd1\_Pe0\_pUb1]} \cdot V\text{\_dnabound} \cdot 0.95)\\
&+ (\text{[dnabound\_Foxo1\_Pa0\_Pd1\_Pe1\_pUb0]} \cdot V\text{\_dnabound} \cdot 0.95)\\
&+ (\text{[dnabound\_Foxo1\_Pa0\_Pd1\_Pe1\_pUb1]} \cdot V\text{\_dnabound} \cdot 0.95)\\
&+ (\text{[dnabound\_Foxo1\_Pa1\_Pd0\_Pe0\_pUb0]} \cdot V\text{\_dnabound} \cdot 0.95)\\
&+ (\text{[dnabound\_Foxo1\_Pa1\_Pd0\_Pe0\_pUb1]} \cdot V\text{\_dnabound} \cdot 0.95)\\
&+ (\text{[dnabound\_Foxo1\_Pa1\_Pd0\_Pe1\_pUb0]} \cdot V\text{\_dnabound} \cdot 0.95)\\
&+ (\text{[dnabound\_Foxo1\_Pa1\_Pd0\_Pe1\_pUb1]} \cdot V\text{\_dnabound} \cdot 0.95)\\
&+ (\text{[dnabound\_Foxo1\_Pa1\_Pd1\_Pe0\_pUb0]} \cdot V\text{\_dnabound} \cdot 0.95)\\
&+ (\text{[dnabound\_Foxo1\_Pa1\_Pd1\_Pe0\_pUb1]} \cdot V\text{\_dnabound} \cdot 0.95)\\
&+ (\text{[dnabound\_Foxo1\_Pa1\_Pd1\_Pe1\_pUb0]} \cdot V\text{\_dnabound} \cdot 0.95)\\
&+ (\text{[dnabound\_Foxo1\_Pa1\_Pd1\_Pe1\_pUb1]} \cdot V\text{\_dnabound} \cdot 0.95)\\
&+ (15)\\
&- (\text{[nucleus\_RNA\_SOD2]} \cdot V\text{\_nucleus} \cdot 0.22)
\end{aligned}
$$

$$
\begin{aligned}
\frac{\mathrm{d}\left(\text{[dnabound\_Foxo1\_Pa0\_Pd0\_Pe0\_pUb0]} \cdot V\text{\_dnabound}\right)}{\mathrm{d}t} =\; &- V\text{\_dnabound} \cdot (\text{[dnabound\_Foxo1\_Pa0\_Pd0\_Pe0\_pUb0]} \cdot \text{[Akt\_P2]} \cdot V\text{\_cytoplasm} \cdot 0.0003)\\
&- V\text{\_dnabound} \cdot (\text{[dnabound\_Foxo1\_Pa0\_Pd0\_Pe0\_pUb0]} \cdot \text{[SGK]} \cdot V\text{\_cytoplasm} \cdot 0.0003)\\
&+ V\text{\_dnabound} \cdot (\text{[dnabound\_Foxo1\_Pa1\_Pd0\_Pe0\_pUb0]} \cdot \text{[PP2A]} \cdot V\text{\_cytoplasm} \cdot 1e-06)\\
&- V\text{\_dnabound} \cdot (\text{[dnabound\_Foxo1\_Pa0\_Pd0\_Pe0\_pUb0]} \cdot \text{[IKK\_P]} \cdot V\text{\_cytoplasm} \cdot \text{by\_ikk\_phos\_factor} \cdot 5e-05)\\
&+ V\text{\_dnabound} \cdot (\text{[dnabound\_Foxo1\_Pa0\_Pd1\_Pe0\_pUb0]} \cdot \text{[PP2A]} \cdot V\text{\_cytoplasm} \cdot 1e-06)\\
&- V\text{\_dnabound} \cdot (\text{[dnabound\_Foxo1\_Pa0\_Pd0\_Pe0\_pUb0]} \cdot \text{[JNK\_P]} \cdot V\text{\_cytoplasm} \cdot \text{by\_jnk\_phos\_factor} \cdot 5e-05)\\
&+ V\text{\_dnabound} \cdot (\text{[dnabound\_Foxo1\_Pa0\_Pd0\_Pe1\_pUb0]} \cdot \text{[PP2A]} \cdot V\text{\_cytoplasm} \cdot 1e-06)\\
&- V\text{\_dnabound} \cdot (\text{[dnabound\_Foxo1\_Pa0\_Pd0\_Pe0\_pUb0]} \cdot \text{[SCF]} \cdot V\text{\_cytoplasm} \cdot 1e-06)\\
&+ (\text{[nucleus\_Foxo1\_Pa0\_Pd0\_Pe0\_pUb0]} \cdot V\text{\_nucleus} \cdot 0.25)\\
&- (\text{[dnabound\_Foxo1\_Pa0\_Pd0\_Pe0\_pUb0]} \cdot V\text{\_dnabound} \cdot 0.125)
\end{aligned}
$$

$$
\begin{aligned}
\frac{\mathrm{d}\left(\text{[dnabound\_Foxo1\_Pa0\_Pd0\_Pe0\_pUb1]} \cdot V\text{\_dnabound}\right)}{\mathrm{d}t} =\; &- V\text{\_dnabound} \cdot (\text{[dnabound\_Foxo1\_Pa0\_Pd0\_Pe0\_pUb1]} \cdot \text{[Akt\_P2]} \cdot V\text{\_cytoplasm} \cdot 0.0003)\\
&- V\text{\_dnabound} \cdot (\text{[dnabound\_Foxo1\_Pa0\_Pd0\_Pe0\_pUb1]} \cdot \text{[SGK]} \cdot V\text{\_cytoplasm} \cdot 0.0003)\\
&+ V\text{\_dnabound} \cdot (\text{[dnabound\_Foxo1\_Pa1\_Pd0\_Pe0\_pUb1]} \cdot \text{[PP2A]} \cdot V\text{\_cytoplasm} \cdot 1e-06)\\
&- V\text{\_dnabound} \cdot (\text{[dnabound\_Foxo1\_Pa0\_Pd0\_Pe0\_pUb1]} \cdot \text{[IKK\_P]} \cdot V\text{\_cytoplasm} \cdot \text{by\_ikk\_phos\_factor} \cdot 5e-05)\\
&+ V\text{\_dnabound} \cdot (\text{[dnabound\_Foxo1\_Pa0\_Pd1\_Pe0\_pUb1]} \cdot \text{[PP2A]} \cdot V\text{\_cytoplasm} \cdot 1e-06)\\
&- V\text{\_dnabound} \cdot (\text{[dnabound\_Foxo1\_Pa0\_Pd0\_Pe0\_pUb1]} \cdot \text{[JNK\_P]} \cdot V\text{\_cytoplasm} \cdot \text{by\_jnk\_phos\_factor} \cdot 5e-05)\\
&+ V\text{\_dnabound} \cdot (\text{[dnabound\_Foxo1\_Pa0\_Pd0\_Pe1\_pUb1]} \cdot \text{[PP2A]} \cdot V\text{\_cytoplasm} \cdot 1e-06)\\
&+ V\text{\_dnabound} \cdot (\text{[dnabound\_Foxo1\_Pa0\_Pd0\_Pe0\_pUb0]} \cdot \text{[SCF]} \cdot V\text{\_cytoplasm} \cdot 1e-06)\\
&+ (\text{[nucleus\_Foxo1\_Pa0\_Pd0\_Pe0\_pUb1]} \cdot V\text{\_nucleus} \cdot 0.25)\\
&- (\text{[dnabound\_Foxo1\_Pa0\_Pd0\_Pe0\_pUb1]} \cdot V\text{\_dnabound} \cdot 0.125)
\end{aligned}
$$

$$
\begin{aligned}
\frac{\mathrm{d}\left(\text{[dnabound\_Foxo1\_Pa0\_Pd0\_Pe1\_pUb0]} \cdot V\text{\_dnabound}\right)}{\mathrm{d}t} =\; &- V\text{\_dnabound} \cdot (\text{[dnabound\_Foxo1\_Pa0\_Pd0\_Pe1\_pUb0]} \cdot \text{[Akt\_P2]} \cdot V\text{\_cytoplasm} \cdot 0.0003)\\
&- V\text{\_dnabound} \cdot (\text{[dnabound\_Foxo1\_Pa0\_Pd0\_Pe1\_pUb0]} \cdot \text{[SGK]} \cdot V\text{\_cytoplasm} \cdot 0.0003)\\
&+ V\text{\_dnabound} \cdot (\text{[dnabound\_Foxo1\_Pa1\_Pd0\_Pe1\_pUb0]} \cdot \text{[PP2A]} \cdot V\text{\_cytoplasm} \cdot 1e-06)\\
&- V\text{\_dnabound} \cdot (\text{[dnabound\_Foxo1\_Pa0\_Pd0\_Pe1\_pUb0]} \cdot \text{[IKK\_P]} \cdot V\text{\_cytoplasm} \cdot \text{by\_ikk\_phos\_factor} \cdot 5e-05)\\
&+ V\text{\_dnabound} \cdot (\text{[dnabound\_Foxo1\_Pa0\_Pd1\_Pe1\_pUb0]} \cdot \text{[PP2A]} \cdot V\text{\_cytoplasm} \cdot 1e-06)\\
&+ V\text{\_dnabound} \cdot (\text{[dnabound\_Foxo1\_Pa0\_Pd0\_Pe0\_pUb0]} \cdot \text{[JNK\_P]} \cdot V\text{\_cytoplasm} \cdot \text{by\_jnk\_phos\_factor} \cdot 5e-05)\\
&- V\text{\_dnabound} \cdot (\text{[dnabound\_Foxo1\_Pa0\_Pd0\_Pe1\_pUb0]} \cdot \text{[PP2A]} \cdot V\text{\_cytoplasm} \cdot 1e-06)\\
&- V\text{\_dnabound} \cdot (\text{[dnabound\_Foxo1\_Pa0\_Pd0\_Pe1\_pUb0]} \cdot \text{[SCF]} \cdot V\text{\_cytoplasm} \cdot 1e-06)\\
&+ (\text{[nucleus\_Foxo1\_Pa0\_Pd0\_Pe1\_pUb0]} \cdot V\text{\_nucleus} \cdot 0.25)\\
&- (\text{[dnabound\_Foxo1\_Pa0\_Pd0\_Pe1\_pUb0]} \cdot V\text{\_dnabound} \cdot 0.125)
\end{aligned}
$$

$$
\begin{aligned}
\frac{\mathrm{d}\left(\text{[dnabound\_Foxo1\_Pa0\_Pd0\_Pe1\_pUb1]} \cdot V\text{\_dnabound}\right)}{\mathrm{d}t} =\; &- V\text{\_dnabound} \cdot (\text{[dnabound\_Foxo1\_Pa0\_Pd0\_Pe1\_pUb1]} \cdot \text{[Akt\_P2]} \cdot V\text{\_cytoplasm} \cdot 0.0003)\\
&- V\text{\_dnabound} \cdot (\text{[dnabound\_Foxo1\_Pa0\_Pd0\_Pe1\_pUb1]} \cdot \text{[SGK]} \cdot V\text{\_cytoplasm} \cdot 0.0003)\\
&+ V\text{\_dnabound} \cdot (\text{[dnabound\_Foxo1\_Pa1\_Pd0\_Pe1\_pUb1]} \cdot \text{[PP2A]} \cdot V\text{\_cytoplasm} \cdot 1e-06)\\
&- V\text{\_dnabound} \cdot (\text{[dnabound\_Foxo1\_Pa0\_Pd0\_Pe1\_pUb1]} \cdot \text{[IKK\_P]} \cdot V\text{\_cytoplasm} \cdot \text{by\_ikk\_phos\_factor} \cdot 5e-05)\\
&+ V\text{\_dnabound} \cdot (\text{[dnabound\_Foxo1\_Pa0\_Pd1\_Pe1\_pUb1]} \cdot \text{[PP2A]} \cdot V\text{\_cytoplasm} \cdot 1e-06)\\
&+ V\text{\_dnabound} \cdot (\text{[dnabound\_Foxo1\_Pa0\_Pd0\_Pe0\_pUb1]} \cdot \text{[JNK\_P]} \cdot V\text{\_cytoplasm} \cdot \text{by\_jnk\_phos\_factor} \cdot 5e-05)\\
&- V\text{\_dnabound} \cdot (\text{[dnabound\_Foxo1\_Pa0\_Pd0\_Pe1\_pUb1]} \cdot \text{[PP2A]} \cdot V\text{\_cytoplasm} \cdot 1e-06)\\
&+ V\text{\_dnabound} \cdot (\text{[dnabound\_Foxo1\_Pa0\_Pd0\_Pe1\_pUb0]} \cdot \text{[SCF]} \cdot V\text{\_cytoplasm} \cdot 1e-06)\\
&+ (\text{[nucleus\_Foxo1\_Pa0\_Pd0\_Pe1\_pUb1]} \cdot V\text{\_nucleus} \cdot 0.25)\\
&- (\text{[dnabound\_Foxo1\_Pa0\_Pd0\_Pe1\_pUb1]} \cdot V\text{\_dnabound} \cdot 0.125)
\end{aligned}
$$

$$
\begin{aligned}
\frac{\mathrm{d}\left(\text{[dnabound\_Foxo1\_Pa0\_Pd1\_Pe0\_pUb0]} \cdot V\text{\_dnabound}\right)}{\mathrm{d}t} =\; &- V\text{\_dnabound} \cdot (\text{[dnabound\_Foxo1\_Pa0\_Pd1\_Pe0\_pUb0]} \cdot \text{[Akt\_P2]} \cdot V\text{\_cytoplasm} \cdot 0.0003)\\
&- V\text{\_dnabound} \cdot (\text{[dnabound\_Foxo1\_Pa0\_Pd1\_Pe0\_pUb0]} \cdot \text{[SGK]} \cdot V\text{\_cytoplasm} \cdot 0.0003)\\
&+ V\text{\_dnabound} \cdot (\text{[dnabound\_Foxo1\_Pa1\_Pd1\_Pe0\_pUb0]} \cdot \text{[PP2A]} \cdot V\text{\_cytoplasm} \cdot 1e-06)\\
&+ V\text{\_dnabound} \cdot (\text{[dnabound\_Foxo1\_Pa0\_Pd0\_Pe0\_pUb0]} \cdot \text{[IKK\_P]} \cdot V\text{\_cytoplasm} \cdot \text{by\_ikk\_phos\_factor} \cdot 5e-05)\\
&- V\text{\_dnabound} \cdot (\text{[dnabound\_Foxo1\_Pa0\_Pd1\_Pe0\_pUb0]} \cdot \text{[PP2A]} \cdot V\text{\_cytoplasm} \cdot 1e-06)\\
&- V\text{\_dnabound} \cdot (\text{[dnabound\_Foxo1\_Pa0\_Pd1\_Pe0\_pUb0]} \cdot \text{[JNK\_P]} \cdot V\text{\_cytoplasm} \cdot \text{by\_jnk\_phos\_factor} \cdot 5e-05)\\
&+ V\text{\_dnabound} \cdot (\text{[dnabound\_Foxo1\_Pa0\_Pd1\_Pe1\_pUb0]} \cdot \text{[PP2A]} \cdot V\text{\_cytoplasm} \cdot 1e-06)\\
&- V\text{\_dnabound} \cdot (\text{[dnabound\_Foxo1\_Pa0\_Pd1\_Pe0\_pUb0]} \cdot \text{[SCF]} \cdot V\text{\_cytoplasm} \cdot 2.2e-05)\\
&+ (\text{[nucleus\_Foxo1\_Pa0\_Pd1\_Pe0\_pUb0]} \cdot V\text{\_nucleus} \cdot 0.25)
\end{aligned}
$$

$$
\begin{aligned}
&- ([\mathrm{dnabound\_Foxo1\_Pa0\_Pd1\_Pe0\_pUb0}] \cdot V\mathrm{\_dnabound} \cdot 0.125)
\end{aligned}
$$

$$
\begin{aligned}
\frac{\mathrm{d}([\mathrm{dnabound\_Foxo1\_Pa0\_Pd1\_Pe0\_pUb1}] \cdot V\mathrm{\_dnabound})}{\mathrm{d}t} = &- V\mathrm{\_dnabound} \cdot ([\mathrm{dnabound\_Foxo1\_Pa0\_Pd1\_Pe0\_pUb1}] \cdot [\mathrm{Akt\_P2}] \cdot V\mathrm{\_cytoplasm} \cdot 0.0003) \\
&- V\mathrm{\_dnabound} \cdot ([\mathrm{dnabound\_Foxo1\_Pa0\_Pd1\_Pe0\_pUb1}] \cdot [\mathrm{SGK}] \cdot V\mathrm{\_cytoplasm} \cdot 0.0003) \\
&+ V\mathrm{\_dnabound} \cdot ([\mathrm{dnabound\_Foxo1\_Pa1\_Pd1\_Pe0\_pUb1}] \cdot [\mathrm{PP2A}] \cdot V\mathrm{\_cytoplasm} \cdot 1e-06) \\
&+ V\mathrm{\_dnabound} \cdot ([\mathrm{dnabound\_Foxo1\_Pa0\_Pd0\_Pe0\_pUb1}] \cdot [\mathrm{IKK\_P}] \cdot V\mathrm{\_cytoplasm} \cdot \mathrm{by\_ikk\_phos\_factor} \cdot 5e-05) \\
&- V\mathrm{\_dnabound} \cdot ([\mathrm{dnabound\_Foxo1\_Pa0\_Pd1\_Pe0\_pUb1}] \cdot [\mathrm{PP2A}] \cdot V\mathrm{\_cytoplasm} \cdot 1e-06) \\
&- V\mathrm{\_dnabound} \cdot ([\mathrm{dnabound\_Foxo1\_Pa0\_Pd1\_Pe0\_pUb1}] \cdot [\mathrm{JNK\_P}] \cdot V\mathrm{\_cytoplasm} \cdot \mathrm{by\_jnk\_phos\_factor} \cdot 5e-05) \\
&+ V\mathrm{\_dnabound} \cdot ([\mathrm{dnabound\_Foxo1\_Pa0\_Pd1\_Pe1\_pUb1}] \cdot [\mathrm{PP2A}] \cdot V\mathrm{\_cytoplasm} \cdot 1e-06) \\
&+ V\mathrm{\_dnabound} \cdot ([\mathrm{dnabound\_Foxo1\_Pa0\_Pd1\_Pe0\_pUb0}] \cdot [\mathrm{SCF}] \cdot V\mathrm{\_cytoplasm} \cdot 2.2e-05) \\
&+ ([\mathrm{nucleus\_Foxo1\_Pa0\_Pd1\_Pe0\_pUb1}] \cdot V\mathrm{\_nucleus} \cdot 0.25) \\
&- ([\mathrm{dnabound\_Foxo1\_Pa0\_Pd1\_Pe0\_pUb1}] \cdot V\mathrm{\_dnabound} \cdot 0.125)
\end{aligned}
$$

$$
\begin{aligned}
\frac{\mathrm{d}([\mathrm{dnabound\_Foxo1\_Pa0\_Pd1\_Pe1\_pUb0}] \cdot V\mathrm{\_dnabound})}{\mathrm{d}t} = &- V\mathrm{\_dnabound} \cdot ([\mathrm{dnabound\_Foxo1\_Pa0\_Pd1\_Pe1\_pUb0}] \cdot [\mathrm{Akt\_P2}] \cdot V\mathrm{\_cytoplasm} \cdot 0.0003) \\
&- V\mathrm{\_dnabound} \cdot ([\mathrm{dnabound\_Foxo1\_Pa0\_Pd1\_Pe1\_pUb0}] \cdot [\mathrm{SGK}] \cdot V\mathrm{\_cytoplasm} \cdot 0.0003) \\
&+ V\mathrm{\_dnabound} \cdot ([\mathrm{dnabound\_Foxo1\_Pa1\_Pd1\_Pe1\_pUb0}] \cdot [\mathrm{PP2A}] \cdot V\mathrm{\_cytoplasm} \cdot 1e-06) \\
&+ V\mathrm{\_dnabound} \cdot ([\mathrm{dnabound\_Foxo1\_Pa0\_Pd0\_Pe1\_pUb0}] \cdot [\mathrm{IKK\_P}] \cdot V\mathrm{\_cytoplasm} \cdot \mathrm{by\_ikk\_phos\_factor} \cdot 5e-05) \\
&- V\mathrm{\_dnabound} \cdot ([\mathrm{dnabound\_Foxo1\_Pa0\_Pd1\_Pe1\_pUb0}] \cdot [\mathrm{PP2A}] \cdot V\mathrm{\_cytoplasm} \cdot 1e-06) \\
&+ V\mathrm{\_dnabound} \cdot ([\mathrm{dnabound\_Foxo1\_Pa0\_Pd1\_Pe0\_pUb0}] \cdot [\mathrm{JNK\_P}] \cdot V\mathrm{\_cytoplasm} \cdot \mathrm{by\_jnk\_phos\_factor} \cdot 5e-05) \\
&- V\mathrm{\_dnabound} \cdot ([\mathrm{dnabound\_Foxo1\_Pa0\_Pd1\_Pe1\_pUb0}] \cdot [\mathrm{PP2A}] \cdot V\mathrm{\_cytoplasm} \cdot 1e-06) \\
&- V\mathrm{\_dnabound} \cdot ([\mathrm{dnabound\_Foxo1\_Pa0\_Pd1\_Pe1\_pUb0}] \cdot [\mathrm{SCF}] \cdot V\mathrm{\_cytoplasm} \cdot 2.2e-05) \\
&+ ([\mathrm{nucleus\_Foxo1\_Pa0\_Pd1\_Pe1\_pUb0}] \cdot V\mathrm{\_nucleus} \cdot 0.25) \\
&- ([\mathrm{dnabound\_Foxo1\_Pa0\_Pd1\_Pe1\_pUb0}] \cdot V\mathrm{\_dnabound} \cdot 0.125)
\end{aligned}
$$

$$
\begin{aligned}
\frac{\mathrm{d}([\mathrm{dnabound\_Foxo1\_Pa0\_Pd1\_Pe1\_pUb1}] \cdot V\mathrm{\_dnabound})}{\mathrm{d}t} = &- V\mathrm{\_dnabound} \cdot ([\mathrm{dnabound\_Foxo1\_Pa0\_Pd1\_Pe1\_pUb1}] \cdot [\mathrm{Akt\_P2}] \cdot V\mathrm{\_cytoplasm} \cdot 0.0003) \\
&- V\mathrm{\_dnabound} \cdot ([\mathrm{dnabound\_Foxo1\_Pa0\_Pd1\_Pe1\_pUb1}] \cdot [\mathrm{SGK}] \cdot V\mathrm{\_cytoplasm} \cdot 0.0003) \\
&+ V\mathrm{\_dnabound} \cdot ([\mathrm{dnabound\_Foxo1\_Pa1\_Pd1\_Pe1\_pUb1}] \cdot [\mathrm{PP2A}] \cdot V\mathrm{\_cytoplasm} \cdot 1e-06) \\
&+ V\mathrm{\_dnabound} \cdot ([\mathrm{dnabound\_Foxo1\_Pa0\_Pd0\_Pe1\_pUb1}] \cdot [\mathrm{IKK\_P}] \cdot V\mathrm{\_cytoplasm} \cdot \mathrm{by\_ikk\_phos\_factor} \cdot 5e-05) \\
&- V\mathrm{\_dnabound} \cdot ([\mathrm{dnabound\_Foxo1\_Pa0\_Pd1\_Pe1\_pUb1}] \cdot [\mathrm{PP2A}] \cdot V\mathrm{\_cytoplasm} \cdot 1e-06) \\
&+ V\mathrm{\_dnabound} \cdot ([\mathrm{dnabound\_Foxo1\_Pa0\_Pd1\_Pe0\_pUb1}] \cdot [\mathrm{JNK\_P}] \cdot V\mathrm{\_cytoplasm} \cdot \mathrm{by\_jnk\_phos\_factor} \cdot 5e-05) \\
&- V\mathrm{\_dnabound} \cdot ([\mathrm{dnabound\_Foxo1\_Pa0\_Pd1\_Pe1\_pUb1}] \cdot [\mathrm{PP2A}] \cdot V\mathrm{\_cytoplasm} \cdot 1e-06) \\
&+ V\mathrm{\_dnabound} \cdot ([\mathrm{dnabound\_Foxo1\_Pa0\_Pd1\_Pe1\_pUb0}] \cdot [\mathrm{SCF}] \cdot V\mathrm{\_cytoplasm} \cdot 2.2e-05) \\
&+ ([\mathrm{nucleus\_Foxo1\_Pa0\_Pd1\_Pe1\_pUb1}] \cdot V\mathrm{\_nucleus} \cdot 0.25) \\
&- ([\mathrm{dnabound\_Foxo1\_Pa0\_Pd1\_Pe1\_pUb1}] \cdot V\mathrm{\_dnabound} \cdot 0.125)
\end{aligned}
$$

$$
\begin{aligned}
\frac{\mathrm{d}([\mathrm{dnabound\_Foxo1\_Pa1\_Pd0\_Pe0\_pUb0}] \cdot V\mathrm{\_dnabound})}{\mathrm{d}t} = &+ V\mathrm{\_dnabound} \cdot ([\mathrm{dnabound\_Foxo1\_Pa0\_Pd0\_Pe0\_pUb0}] \cdot [\mathrm{Akt\_P2}] \cdot V\mathrm{\_cytoplasm} \cdot 0.0003) \\
&+ V\mathrm{\_dnabound} \cdot ([\mathrm{dnabound\_Foxo1\_Pa0\_Pd0\_Pe0\_pUb0}] \cdot [\mathrm{SGK}] \cdot V\mathrm{\_cytoplasm} \cdot 0.0003) \\
&- V\mathrm{\_dnabound} \cdot ([\mathrm{dnabound\_Foxo1\_Pa1\_Pd0\_Pe0\_pUb0}] \cdot [\mathrm{PP2A}] \cdot V\mathrm{\_cytoplasm} \cdot 1e-06) \\
&- V\mathrm{\_dnabound} \cdot ([\mathrm{dnabound\_Foxo1\_Pa1\_Pd0\_Pe0\_pUb0}] \cdot [\mathrm{IKK\_P}] \cdot V\mathrm{\_cytoplasm} \cdot \mathrm{by\_ikk\_phos\_factor} \cdot 5e-05) \\
&+ V\mathrm{\_dnabound} \cdot ([\mathrm{dnabound\_Foxo1\_Pa1\_Pd1\_Pe0\_pUb0}] \cdot [\mathrm{PP2A}] \cdot V\mathrm{\_cytoplasm} \cdot 1e-06) \\
&- V\mathrm{\_dnabound} \cdot ([\mathrm{dnabound\_Foxo1\_Pa1\_Pd0\_Pe0\_pUb0}] \cdot [\mathrm{JNK\_P}] \cdot V\mathrm{\_cytoplasm} \cdot \mathrm{by\_jnk\_phos\_factor} \cdot 5e-05) \\
&+ V\mathrm{\_dnabound} \cdot ([\mathrm{dnabound\_Foxo1\_Pa1\_Pd0\_Pe1\_pUb0}] \cdot [\mathrm{PP2A}] \cdot V\mathrm{\_cytoplasm} \cdot 1e-06) \\
&- V\mathrm{\_dnabound} \cdot ([\mathrm{dnabound\_Foxo1\_Pa1\_Pd0\_Pe0\_pUb0}] \cdot [\mathrm{SCF}] \cdot V\mathrm{\_cytoplasm} \cdot 3e-06) \\
&+ ([\mathrm{nucleus\_Foxo1\_Pa1\_Pd0\_Pe0\_pUb0}] \cdot V\mathrm{\_nucleus} \cdot 0.125) \\
&- ([\mathrm{dnabound\_Foxo1\_Pa1\_Pd0\_Pe0\_pUb0}] \cdot V\mathrm{\_dnabound} \cdot 0.125)
\end{aligned}
$$

$$
\begin{aligned}
\frac{\mathrm{d}([\mathrm{dnabound\_Foxo1\_Pa1\_Pd0\_Pe0\_pUb1}] \cdot V\mathrm{\_dnabound})}{\mathrm{d}t} = &- ([\mathrm{dnabound\_Foxo1\_Pa1\_Pd0\_Pe0\_pUb1}] \cdot V\mathrm{\_dnabound} \cdot 0.125) \\
&+ V\mathrm{\_dnabound} \cdot ([\mathrm{dnabound\_Foxo1\_Pa0\_Pd0\_Pe0\_pUb1}] \cdot [\mathrm{Akt\_P2}] \cdot V\mathrm{\_cytoplasm} \cdot 0.0003) \\
&+ V\mathrm{\_dnabound} \cdot ([\mathrm{dnabound\_Foxo1\_Pa0\_Pd0\_Pe0\_pUb1}] \cdot [\mathrm{SGK}] \cdot V\mathrm{\_cytoplasm} \cdot 0.0003) \\
&- V\mathrm{\_dnabound} \cdot ([\mathrm{dnabound\_Foxo1\_Pa1\_Pd0\_Pe0\_pUb1}] \cdot [\mathrm{PP2A}] \cdot V\mathrm{\_cytoplasm} \cdot 1e-06) \\
&- V\mathrm{\_dnabound} \cdot ([\mathrm{dnabound\_Foxo1\_Pa1\_Pd0\_Pe0\_pUb1}] \cdot [\mathrm{IKK\_P}] \cdot V\mathrm{\_cytoplasm} \cdot \mathrm{by\_ikk\_phos\_factor} \cdot 5e-05) \\
&+ V\mathrm{\_dnabound} \cdot ([\mathrm{dnabound\_Foxo1\_Pa1\_Pd1\_Pe0\_pUb1}] \cdot [\mathrm{PP2A}] \cdot V\mathrm{\_cytoplasm} \cdot 1e-06) \\
&- V\mathrm{\_dnabound} \cdot ([\mathrm{dnabound\_Foxo1\_Pa1\_Pd0\_Pe0\_pUb1}] \cdot [\mathrm{JNK\_P}] \cdot V\mathrm{\_cytoplasm} \cdot \mathrm{by\_jnk\_phos\_factor} \cdot 5e-05) \\
&+ V\mathrm{\_dnabound} \cdot ([\mathrm{dnabound\_Foxo1\_Pa1\_Pd0\_Pe1\_pUb1}] \cdot [\mathrm{PP2A}] \cdot V\mathrm{\_cytoplasm} \cdot 1e-06) \\
&+ V\mathrm{\_dnabound} \cdot ([\mathrm{dnabound\_Foxo1\_Pa1\_Pd0\_Pe0\_pUb0}] \cdot [\mathrm{SCF}] \cdot V\mathrm{\_cytoplasm} \cdot 3e-06) \\
&+ ([\mathrm{nucleus\_Foxo1\_Pa1\_Pd0\_Pe0\_pUb1}] \cdot V\mathrm{\_nucleus} \cdot 0.125)
\end{aligned}
$$

$$
\begin{aligned}
\frac{\mathrm{d}([\mathrm{dnabound\_Foxo1\_Pa1\_Pd0\_Pe1\_pUb0}] \cdot V\mathrm{\_dnabound})}{\mathrm{d}t} = &+ ([\mathrm{nucleus\_Foxo1\_Pa1\_Pd0\_Pe1\_pUb0}] \cdot V\mathrm{\_nucleus} \cdot 0.125) \\
&- ([\mathrm{dnabound\_Foxo1\_Pa1\_Pd0\_Pe1\_pUb0}] \cdot V\mathrm{\_dnabound} \cdot 0.125) \\
&+ V\mathrm{\_dnabound} \cdot ([\mathrm{dnabound\_Foxo1\_Pa0\_Pd0\_Pe1\_pUb0}] \cdot [\mathrm{Akt\_P2}] \cdot V\mathrm{\_cytoplasm} \cdot 0.0003) \\
&+ V\mathrm{\_dnabound} \cdot ([\mathrm{dnabound\_Foxo1\_Pa0\_Pd0\_Pe1\_pUb0}] \cdot [\mathrm{SGK}] \cdot V\mathrm{\_cytoplasm} \cdot 0.0003) \\
&- V\mathrm{\_dnabound} \cdot ([\mathrm{dnabound\_Foxo1\_Pa1\_Pd0\_Pe1\_pUb0}] \cdot [\mathrm{PP2A}] \cdot V\mathrm{\_cytoplasm} \cdot 1e-06) \\
&- V\mathrm{\_dnabound} \cdot ([\mathrm{dnabound\_Foxo1\_Pa1\_Pd0\_Pe1\_pUb0}] \cdot [\mathrm{IKK\_P}] \cdot V\mathrm{\_cytoplasm} \cdot \mathrm{by\_ikk\_phos\_factor} \cdot 5e-05) \\
&+ V\mathrm{\_dnabound} \cdot ([\mathrm{dnabound\_Foxo1\_Pa1\_Pd1\_Pe1\_pUb0}] \cdot [\mathrm{PP2A}] \cdot V\mathrm{\_cytoplasm} \cdot 1e-06) \\
&+ V\mathrm{\_dnabound} \cdot ([\mathrm{dnabound\_Foxo1\_Pa1\_Pd0\_Pe0\_pUb0}] \cdot [\mathrm{JNK\_P}] \cdot V\mathrm{\_cytoplasm} \cdot \mathrm{by\_jnk\_phos\_factor} \cdot 5e-05) \\
&- V\mathrm{\_dnabound} \cdot ([\mathrm{dnabound\_Foxo1\_Pa1\_Pd0\_Pe1\_pUb0}] \cdot [\mathrm{PP2A}] \cdot V\mathrm{\_cytoplasm} \cdot 1e-06) \\
&- V\mathrm{\_dnabound} \cdot ([\mathrm{dnabound\_Foxo1\_Pa1\_Pd0\_Pe1\_pUb0}] \cdot [\mathrm{SCF}] \cdot V\mathrm{\_cytoplasm} \cdot 3e-06)
\end{aligned}
$$

$$
\begin{aligned}
\frac{\mathrm{d}([\mathrm{dnabound\_Foxo1\_Pa1\_Pd0\_Pe1\_pUb1}] \cdot V\mathrm{\_dnabound})}{\mathrm{d}t} = &+ ([\mathrm{nucleus\_Foxo1\_Pa1\_Pd0\_Pe1\_pUb1}] \cdot V\mathrm{\_nucleus} \cdot 0.125) \\
&- ([\mathrm{dnabound\_Foxo1\_Pa1\_Pd0\_Pe1\_pUb1}] \cdot V\mathrm{\_dnabound} \cdot 0.125) \\
&+ V\mathrm{\_dnabound} \cdot ([\mathrm{dnabound\_Foxo1\_Pa0\_Pd0\_Pe1\_pUb1}] \cdot [\mathrm{Akt\_P2}] \cdot V\mathrm{\_cytoplasm} \cdot 0.0003) \\
&+ V\mathrm{\_dnabound} \cdot ([\mathrm{dnabound\_Foxo1\_Pa0\_Pd0\_Pe1\_pUb1}] \cdot [\mathrm{SGK}] \cdot V\mathrm{\_cytoplasm} \cdot 0.0003) \\
&- V\mathrm{\_dnabound} \cdot ([\mathrm{dnabound\_Foxo1\_Pa1\_Pd0\_Pe1\_pUb1}] \cdot [\mathrm{PP2A}] \cdot V\mathrm{\_cytoplasm} \cdot 1e-06) \\
&- V\mathrm{\_dnabound} \cdot ([\mathrm{dnabound\_Foxo1\_Pa1\_Pd0\_Pe1\_pUb1}] \cdot [\mathrm{IKK\_P}] \cdot V\mathrm{\_cytoplasm} \cdot \mathrm{by\_ikk\_phos\_factor} \cdot 5e-05) \\
&+ V\mathrm{\_dnabound} \cdot ([\mathrm{dnabound\_Foxo1\_Pa1\_Pd1\_Pe1\_pUb1}] \cdot [\mathrm{PP2A}] \cdot V\mathrm{\_cytoplasm} \cdot 1e-06) \\
&+ V\mathrm{\_dnabound} \cdot ([\mathrm{dnabound\_Foxo1\_Pa1\_Pd0\_Pe0\_pUb1}] \cdot [\mathrm{JNK\_P}] \cdot V\mathrm{\_cytoplasm} \cdot \mathrm{by\_jnk\_phos\_factor} \cdot 5e-05) \\
&- V\mathrm{\_dnabound} \cdot ([\mathrm{dnabound\_Foxo1\_Pa1\_Pd0\_Pe1\_pUb1}] \cdot [\mathrm{PP2A}] \cdot V\mathrm{\_cytoplasm} \cdot 1e-06) \\
&+ V\mathrm{\_dnabound} \cdot ([\mathrm{dnabound\_Foxo1\_Pa1\_Pd0\_Pe1\_pUb0}] \cdot [\mathrm{SCF}] \cdot V\mathrm{\_cytoplasm} \cdot 3e-06)
\end{aligned}
$$

$$
\begin{aligned}
\frac{\mathrm{d}([\mathrm{dnabound\_Foxo1\_Pa1\_Pd1\_Pe0\_pUb0}] \cdot V\mathrm{\_dnabound})}{\mathrm{d}t} = &+ ([\mathrm{nucleus\_Foxo1\_Pa1\_Pd1\_Pe0\_pUb0}] \cdot V\mathrm{\_nucleus} \cdot 0.125) \\
&- ([\mathrm{dnabound\_Foxo1\_Pa1\_Pd1\_Pe0\_pUb0}] \cdot V\mathrm{\_dnabound} \cdot 0.125) \\
&+ V\mathrm{\_dnabound} \cdot ([\mathrm{dnabound\_Foxo1\_Pa0\_Pd1\_Pe0\_pUb0}] \cdot [\mathrm{Akt\_P2}] \cdot V\mathrm{\_cytoplasm} \cdot 0.0003) \\
&+ V\mathrm{\_dnabound} \cdot ([\mathrm{dnabound\_Foxo1\_Pa0\_Pd1\_Pe0\_pUb0}] \cdot [\mathrm{SGK}] \cdot V\mathrm{\_cytoplasm} \cdot 0.0003) \\
&- V\mathrm{\_dnabound} \cdot ([\mathrm{dnabound\_Foxo1\_Pa1\_Pd1\_Pe0\_pUb0}] \cdot [\mathrm{PP2A}] \cdot V\mathrm{\_cytoplasm} \cdot 1e-06)
\end{aligned}
$$

$$+\,V\text{\_dnabound}\cdot([\text{dnabound\_Foxo1\_Pa1\_Pd0\_Pe0\_pUb0}]\cdot[\text{IKK\_P}]\cdot V\text{\_cytoplasm}\cdot\text{by\_ikk\_phos\_factor}\cdot 5e-05)$$
$$-\,V\text{\_dnabound}\cdot([\text{dnabound\_Foxo1\_Pa1\_Pd1\_Pe0\_pUb0}]\cdot[\text{PP2A}]\cdot V\text{\_cytoplasm}\cdot 1e-06)$$
$$-\,V\text{\_dnabound}\cdot([\text{dnabound\_Foxo1\_Pa1\_Pd1\_Pe0\_pUb0}]\cdot[\text{JNK\_P}]\cdot V\text{\_cytoplasm}\cdot\text{by\_jnk\_phos\_factor}\cdot 5e-05)$$
$$+\,V\text{\_dnabound}\cdot([\text{dnabound\_Foxo1\_Pa1\_Pd1\_Pe1\_pUb0}]\cdot[\text{PP2A}]\cdot V\text{\_cytoplasm}\cdot 1e-06)$$
$$-\,V\text{\_dnabound}\cdot([\text{dnabound\_Foxo1\_Pa1\_Pd1\_Pe0\_pUb0}]\cdot[\text{SCF}]\cdot V\text{\_cytoplasm}\cdot 6.6e-05)$$

$$\frac{\mathrm{d}\left([\text{dnabound\_Foxo1\_Pa1\_Pd1\_Pe0\_pUb1}]\cdot V\text{\_dnabound}\right)}{\mathrm{d}t} = +\,([\text{nucleus\_Foxo1\_Pa1\_Pd1\_Pe0\_pUb1}]\cdot V\text{\_nucleus}\cdot 0.125)$$
$$-\,([\text{dnabound\_Foxo1\_Pa1\_Pd1\_Pe0\_pUb1}]\cdot V\text{\_dnabound}\cdot 0.125)$$
$$+\,V\text{\_dnabound}\cdot([\text{dnabound\_Foxo1\_Pa0\_Pd1\_Pe0\_pUb1}]\cdot[\text{Akt\_P2}]\cdot V\text{\_cytoplasm}\cdot 0.0003)$$
$$+\,V\text{\_dnabound}\cdot([\text{dnabound\_Foxo1\_Pa0\_Pd1\_Pe0\_pUb1}]\cdot[\text{SGK}]\cdot V\text{\_cytoplasm}\cdot 0.0003)$$
$$-\,V\text{\_dnabound}\cdot([\text{dnabound\_Foxo1\_Pa1\_Pd1\_Pe0\_pUb1}]\cdot[\text{PP2A}]\cdot V\text{\_cytoplasm}\cdot 1e-06)$$
$$+\,V\text{\_dnabound}\cdot([\text{dnabound\_Foxo1\_Pa1\_Pd0\_Pe0\_pUb1}]\cdot[\text{IKK\_P}]\cdot V\text{\_cytoplasm}\cdot\text{by\_ikk\_phos\_factor}\cdot 5e-05)$$
$$-\,V\text{\_dnabound}\cdot([\text{dnabound\_Foxo1\_Pa1\_Pd1\_Pe0\_pUb1}]\cdot[\text{PP2A}]\cdot V\text{\_cytoplasm}\cdot 1e-06)$$
$$-\,V\text{\_dnabound}\cdot([\text{dnabound\_Foxo1\_Pa1\_Pd1\_Pe0\_pUb1}]\cdot[\text{JNK\_P}]\cdot V\text{\_cytoplasm}\cdot\text{by\_jnk\_phos\_factor}\cdot 5e-05)$$
$$+\,V\text{\_dnabound}\cdot([\text{dnabound\_Foxo1\_Pa1\_Pd1\_Pe1\_pUb1}]\cdot[\text{PP2A}]\cdot V\text{\_cytoplasm}\cdot 1e-06)$$
$$+\,V\text{\_dnabound}\cdot([\text{dnabound\_Foxo1\_Pa1\_Pd1\_Pe0\_pUb0}]\cdot[\text{SCF}]\cdot V\text{\_cytoplasm}\cdot 6.6e-05)$$

$$\frac{\mathrm{d}\left([\text{dnabound\_Foxo1\_Pa1\_Pd1\_Pe1\_pUb0}]\cdot V\text{\_dnabound}\right)}{\mathrm{d}t} = +\,([\text{nucleus\_Foxo1\_Pa1\_Pd1\_Pe1\_pUb0}]\cdot V\text{\_nucleus}\cdot 0.125)$$
$$-\,([\text{dnabound\_Foxo1\_Pa1\_Pd1\_Pe1\_pUb0}]\cdot V\text{\_dnabound}\cdot 0.125)$$
$$+\,V\text{\_dnabound}\cdot([\text{dnabound\_Foxo1\_Pa0\_Pd1\_Pe1\_pUb0}]\cdot[\text{Akt\_P2}]\cdot V\text{\_cytoplasm}\cdot 0.0003)$$
$$+\,V\text{\_dnabound}\cdot([\text{dnabound\_Foxo1\_Pa0\_Pd1\_Pe1\_pUb0}]\cdot[\text{SGK}]\cdot V\text{\_cytoplasm}\cdot 0.0003)$$
$$-\,V\text{\_dnabound}\cdot([\text{dnabound\_Foxo1\_Pa1\_Pd1\_Pe1\_pUb0}]\cdot[\text{PP2A}]\cdot V\text{\_cytoplasm}\cdot 1e-06)$$
$$+\,V\text{\_dnabound}\cdot([\text{dnabound\_Foxo1\_Pa1\_Pd0\_Pe1\_pUb0}]\cdot[\text{IKK\_P}]\cdot V\text{\_cytoplasm}\cdot\text{by\_ikk\_phos\_factor}\cdot 5e-05)$$
$$-\,V\text{\_dnabound}\cdot([\text{dnabound\_Foxo1\_Pa1\_Pd1\_Pe1\_pUb0}]\cdot[\text{PP2A}]\cdot V\text{\_cytoplasm}\cdot 1e-06)$$
$$+\,V\text{\_dnabound}\cdot([\text{dnabound\_Foxo1\_Pa1\_Pd1\_Pe0\_pUb0}]\cdot[\text{JNK\_P}]\cdot V\text{\_cytoplasm}\cdot\text{by\_jnk\_phos\_factor}\cdot 5e-05)$$
$$-\,V\text{\_dnabound}\cdot([\text{dnabound\_Foxo1\_Pa1\_Pd1\_Pe1\_pUb0}]\cdot[\text{PP2A}]\cdot V\text{\_cytoplasm}\cdot 1e-06)$$
$$-\,V\text{\_dnabound}\cdot([\text{dnabound\_Foxo1\_Pa1\_Pd1\_Pe1\_pUb0}]\cdot[\text{SCF}]\cdot V\text{\_cytoplasm}\cdot 6.6e-05)$$

$$\frac{\mathrm{d}\left([\text{dnabound\_Foxo1\_Pa1\_Pd1\_Pe1\_pUb1}]\cdot V\text{\_dnabound}\right)}{\mathrm{d}t} = +\,([\text{nucleus\_Foxo1\_Pa1\_Pd1\_Pe1\_pUb1}]\cdot V\text{\_nucleus}\cdot 0.125)$$
$$-\,([\text{dnabound\_Foxo1\_Pa1\_Pd1\_Pe1\_pUb1}]\cdot V\text{\_dnabound}\cdot 0.125)$$
$$+\,V\text{\_dnabound}\cdot([\text{dnabound\_Foxo1\_Pa0\_Pd1\_Pe1\_pUb1}]\cdot[\text{Akt\_P2}]\cdot V\text{\_cytoplasm}\cdot 0.0003)$$
$$+\,V\text{\_dnabound}\cdot([\text{dnabound\_Foxo1\_Pa0\_Pd1\_Pe1\_pUb1}]\cdot[\text{SGK}]\cdot V\text{\_cytoplasm}\cdot 0.0003)$$
$$-\,V\text{\_dnabound}\cdot([\text{dnabound\_Foxo1\_Pa1\_Pd1\_Pe1\_pUb1}]\cdot[\text{PP2A}]\cdot V\text{\_cytoplasm}\cdot 1e-06)$$
$$+\,V\text{\_dnabound}\cdot([\text{dnabound\_Foxo1\_Pa1\_Pd0\_Pe1\_pUb1}]\cdot[\text{IKK\_P}]\cdot V\text{\_cytoplasm}\cdot\text{by\_ikk\_phos\_factor}\cdot 5e-05)$$
$$-\,V\text{\_dnabound}\cdot([\text{dnabound\_Foxo1\_Pa1\_Pd1\_Pe1\_pUb1}]\cdot[\text{PP2A}]\cdot V\text{\_cytoplasm}\cdot 1e-06)$$
$$+\,V\text{\_dnabound}\cdot([\text{dnabound\_Foxo1\_Pa1\_Pd1\_Pe0\_pUb1}]\cdot[\text{JNK\_P}]\cdot V\text{\_cytoplasm}\cdot\text{by\_jnk\_phos\_factor}\cdot 5e-05)$$
$$-\,V\text{\_dnabound}\cdot([\text{dnabound\_Foxo1\_Pa1\_Pd1\_Pe1\_pUb1}]\cdot[\text{PP2A}]\cdot V\text{\_cytoplasm}\cdot 1e-06)$$
$$+\,V\text{\_dnabound}\cdot([\text{dnabound\_Foxo1\_Pa1\_Pd1\_Pe1\_pUb0}]\cdot[\text{SCF}]\cdot V\text{\_cytoplasm}\cdot 6.6e-05)$$

$$\text{insconc} = \frac{\text{Ins.ParticleNumber}}{\text{navo}\cdot\text{vextracellular}}$$

$$\text{k\_ros\_perm} = \text{ros\_perm}\cdot\text{membrane\_area}$$

$$\text{ros\_ext\_conc} = \frac{\text{sc\_ros}\cdot\text{extracellular\_ROS.ParticleNumber}}{\text{navo}\cdot V\text{\_extracellular}}$$

$$\text{ros\_cyto\_conc} = \frac{\text{sc\_ros}\cdot\text{ROS.ParticleNumber}}{\text{navo}\cdot V\text{\_cytoplasm}}$$

$$\text{rosconc} = \frac{\text{ROS.ParticleNumber}}{\text{navo}\cdot\text{vcytoplasm}}$$

$$[\text{Foxo1\_Pa0\_tot}] = \frac{\sum_{c\in(\text{cytoplasm,nucleus,dnabound})\,,\ i\in(0,1)\,,\ j\in(0,1)\,,\ k\in(0,1)\,,\ l\in(0,1)} c\text{\_Foxo1\_Pa}i\text{\_Pd}j\text{\_Pe}k\text{\_pUb}l\text{.ParticleNumber}\cdot\delta(i)}{V\text{\_extracellular}}$$

$$[\text{Foxo1\_Pa1\_tot}] = \frac{\sum_{c\in(\text{cytoplasm,nucleus,dnabound})\,,\ i\in(0,1)\,,\ j\in(0,1)\,,\ k\in(0,1)\,,\ l\in(0,1)} c\text{\_Foxo1\_Pa}i\text{\_Pd}j\text{\_Pe}k\text{\_pUb}l\text{.ParticleNumber}\cdot\delta(i-1)}{V\text{\_extracellular}}$$

$$[\text{Foxo1\_Pd0\_tot}] = \frac{\sum_{c\in(\text{cytoplasm,nucleus,dnabound})\,,\ i\in(0,1)\,,\ j\in(0,1)\,,\ k\in(0,1)\,,\ l\in(0,1)} c\text{\_Foxo1\_Pa}i\text{\_Pd}j\text{\_Pe}k\text{\_pUb}l\text{.ParticleNumber}\cdot\delta(j)}{V\text{\_extracellular}}$$

$$[\text{Foxo1\_Pd1\_tot}] = \frac{\sum_{c\in(\text{cytoplasm,nucleus,dnabound})\,,\ i\in(0,1)\,,\ j\in(0,1)\,,\ k\in(0,1)\,,\ l\in(0,1)} c\text{\_Foxo1\_Pa}i\text{\_Pd}j\text{\_Pe}k\text{\_pUb}l\text{.ParticleNumber}\cdot\delta(j-1)}{V\text{\_extracellular}}$$

$$[\text{Foxo1\_Pe0\_tot}] = \frac{\sum_{c\in(\text{cytoplasm,nucleus,dnabound})\,,\ i\in(0,1)\,,\ j\in(0,1)\,,\ k\in(0,1)\,,\ l\in(0,1)} c\text{\_Foxo1\_Pa}i\text{\_Pd}j\text{\_Pe}k\text{\_pUb}l\text{.ParticleNumber}\cdot\delta(k)}{V\text{\_extracellular}}$$

$$[\text{Foxo1\_Pe1\_tot}] = \frac{\sum_{c\in(\text{cytoplasm,nucleus,dnabound})\,,\ i\in(0,1)\,,\ j\in(0,1)\,,\ k\in(0,1)\,,\ l\in(0,1)} c\text{\_Foxo1\_Pa}i\text{\_Pd}j\text{\_Pe}k\text{\_pUb}l\text{.ParticleNumber}\cdot\delta(k-1)}{V\text{\_extracellular}}$$

$$[\text{Foxo1\_pUb0\_tot}] = \frac{\sum_{c\in(\text{cytoplasm,nucleus,dnabound})\,,\ i\in(0,1)\,,\ j\in(0,1)\,,\ k\in(0,1)\,,\ l\in(0,1)} c\text{\_Foxo1\_Pa}i\text{\_Pd}j\text{\_Pe}k\text{\_pUb}l\text{.ParticleNumber}\cdot\delta(l)}{V\text{\_extracellular}}$$

$$[\text{Foxo1\_pUb1\_tot}] = \frac{\sum_{c\in(\text{cytoplasm,nucleus,dnabound})\,,\ i\in(0,1)\,,\ j\in(0,1)\,,\ k\in(0,1)\,,\ l\in(0,1)} c\text{\_Foxo1\_Pa}i\text{\_Pd}j\text{\_Pe}k\text{\_pUb}l\text{.ParticleNumber}\cdot\delta(l-1)}{V\text{\_extracellular}}$$

$$[\text{cytoplasm\_Foxo1\_tot}] = \frac{\sum_{c\in(\text{cytoplasm,nucleus,dnabound})\,,\ i\in(0,1)\,,\ j\in(0,1)\,,\ k\in(0,1)\,,\ l\in(0,1)} c\text{\_Foxo1\_Pa}i\text{\_Pd}j\text{\_Pe}k\text{\_pUb}l\text{.ParticleNumber}\cdot\delta(c=\text{cytoplasm})}{V\text{\_extracellular}}$$

$$[\text{nucleus\_Foxo1\_tot}] = \frac{\sum_{c\in(\text{cytoplasm,nucleus,dnabound})\,,\ i\in(0,1)\,,\ j\in(0,1)\,,\ k\in(0,1)\,,\ l\in(0,1)} c\text{\_Foxo1\_Pa}i\text{\_Pd}j\text{\_Pe}k\text{\_pUb}l\text{.ParticleNumber}\cdot\delta(c=\text{nucleus})}{V\text{\_extracellular}}$$

$$[\text{dnabound\_Foxo1\_tot}] = \frac{\sum_{c\in(\text{cytoplasm,nucleus,dnabound})\,,\ i\in(0,1)\,,\ j\in(0,1)\,,\ k\in(0,1)\,,\ l\in(0,1)} c\text{\_Foxo1\_Pa}i\text{\_Pd}j\text{\_Pe}k\text{\_pUb}l\text{.ParticleNumber}\cdot\delta(c=\text{dnabound})}{V\text{\_extracellular}}$$

$$[\text{Foxo1\_all}] = \frac{\text{cytoplasm\_Foxo1\_tot.ParticleNumber}+\text{nucleus\_Foxo1\_tot.ParticleNumber}+\text{dnabound\_Foxo1\_tot.ParticleNumber}}{V\text{\_extracellular}}$$
