# Supplementary figures and images for: Computational modelling of the regulation of Insulin signalling by oxidative stress
Source: BMC Syst Biol. 2013 May 24;7:41. doi: 10.1186/1752-0509-7-41 (PMC3668293; doi:10.1186/1752-0509-7-41)

Fig S1

A

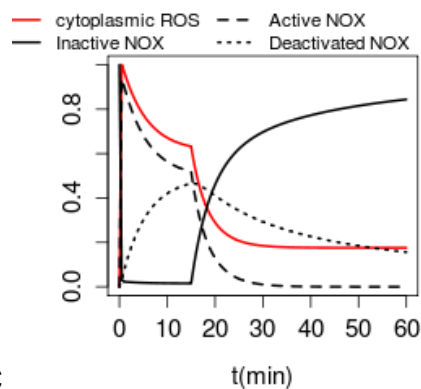

B

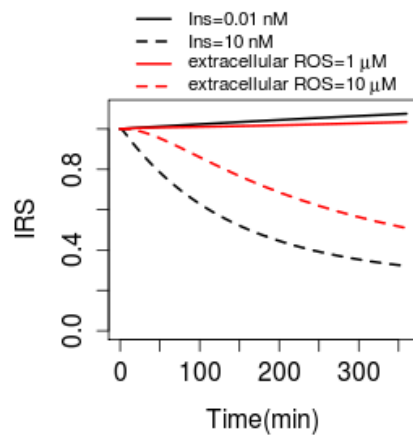

C

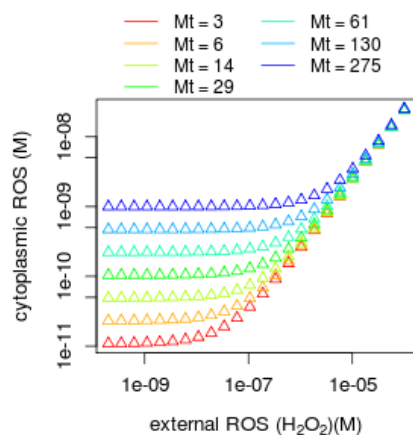

D

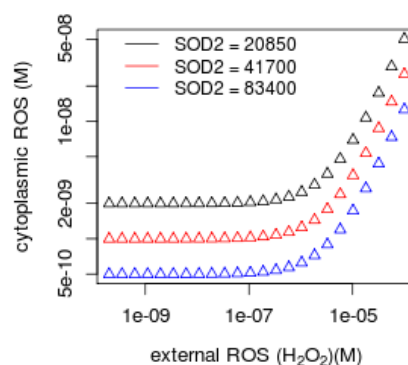

E

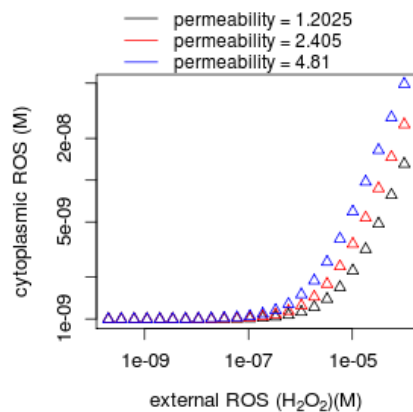

Supplement: Additional file 2 — Description of Data: (A) Model kinetics of NOX activation in response to 15 min insulin, and resulting ROS production; (B) Degradation of IRS1 by prolonged high insulin signalling (black) or high extracellular ROS (red); (C) Effect of variation of basal intracellular ROS production (particle # Mt) on the dependence of intracellular ROS on extracellular ROS; (D) Effect of variation of intracellular antioxidants (particle # of SOD2) on the dependence of intracellular ROS on extracellular ROS; (E) Effect of variation of membrane permeability on the dependence of intracellular ROS on extracellular ROS. [file 1752-0509-7-41-S2.pdf]

Fig S2

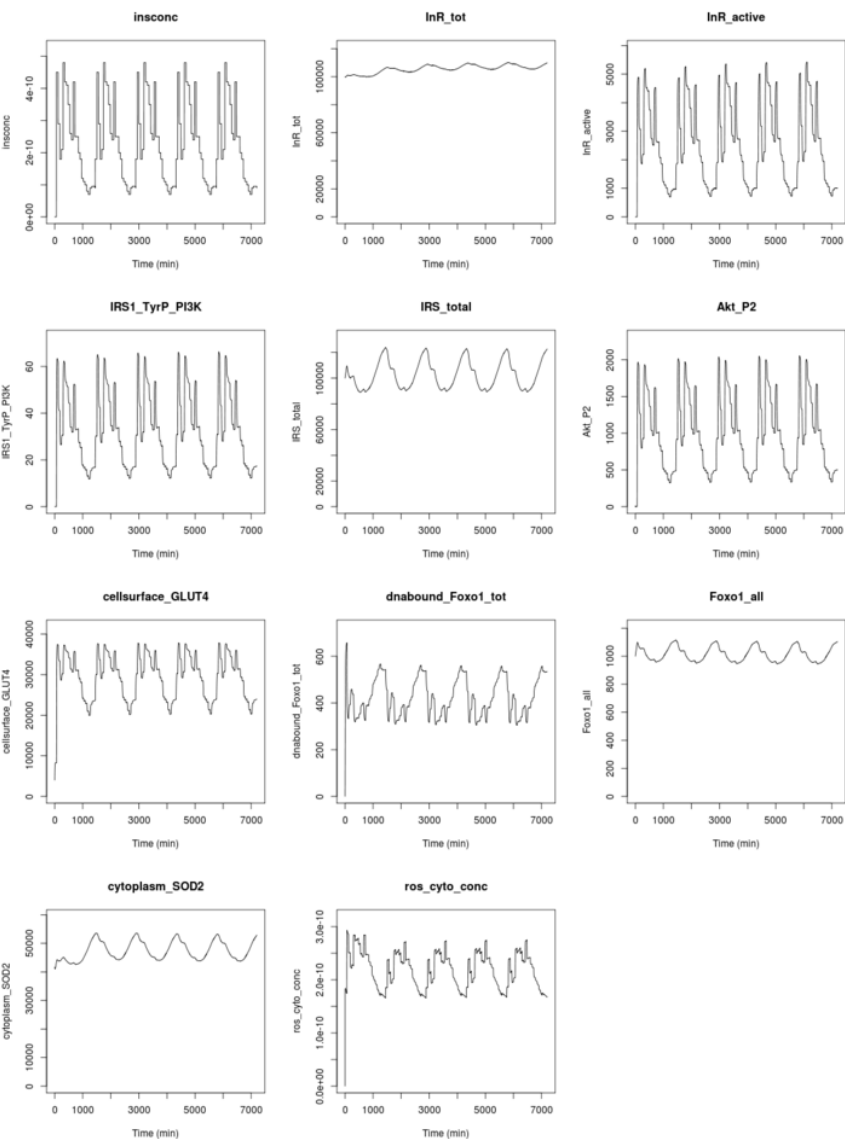

Supplement: Additional file 3 — Description of Data: Behaviour of key model species through 5 days (7200 minutes) of human physiological insulin variation. Insulin data taken from Frayn et al. [73]. [file 1752-0509-7-41-S3.pdf]
