# Supplementary material for: Computational modelling of the regulation of Insulin signalling by oxidative stress
Source: BMC Syst Biol. 2013 May 24;7:41. doi: 10.1186/1752-0509-7-41 (PMC3668293; doi:10.1186/1752-0509-7-41)
Supplement: Additional file 6 — Sensitivity Analysis. [file 1752-0509-7-41-S6.docx]

**Sensitivity Analysis**

We have performed a sensitivity analysis of species concentration on the model parameters, under weak insulin stimulation (0.2nM) and at two conditions of external ROS: zero and 1μM. The sensitivity matrices are included in supplementary material as files 4 and 5. Little is gained in describing the full matrices of such a large model, so we shall discuss the summarized sensitivities, which are root mean square averages of all sensitivities for each parameter. Briefly, all the parameters of the core insulin signalling have moderate or high summary influence, while it is noteworthy that the parameters relating to production of SOD2 have higher influence than those of InR. In the scenario with no external ROS, the parameters of JNK/IKK mediated reactions on IRS and FOXO have low influence, as might be expected since the concentrations of active forms of these kinases will be very low. When external ROS is micromolar, its influence is greater, as is that of transmembrane ROS transport.
