# Supplementary material for: Computational modelling of the regulation of Insulin signalling by oxidative stress
Source: BMC Syst Biol. 2013 May 24;7:41. doi: 10.1186/1752-0509-7-41 (PMC3668293; doi:10.1186/1752-0509-7-41)
Supplement: Additional file 7: Table S5 — Summary of experimental data sets used in fitting the model; see text for additional references. [file 1752-0509-7-41-S7.docx]

Table 5: Summary of experimental data sets used in fitting the model; see text for additional references

| Process | Ref | Experimental System |
| --- | --- | --- |
| insulin binding, PI3K activation, GLUT4 translocation | Stagsted 1993 [1] | rat adipocytes |
| Inhibition of PTPs | Mahadev et al [2, 3] | 3T3-L1 adipocyte-like cells derived from mouse fibroblasts |
| reactivation of PTPs | Lee 1998, [4] | A431 cells (human epidermal carcinoma) |
| Inhibition of PTEN | Lee 2002 [5]; Seo et al 2005[6]. | 3T3 cells and HeLa cells (Lee); human neuroblastoma cells (Seo) |
| ROS transport | Adimora 2010  [7] | Jurkat T cells (human T lymphocytes) |
| IRS Serine phosphorylation, synthesis and degradation | Greene 2003 [8] | H4IIE cells (rat hepatoma) |
| FOXO mediated control of antioxidants | Ambrogini 2010 [9] | mouse osteoblasts |
| interaction of Insulin and hydrogen peroxide | Archuleta, 2009 [10] | Zucker rat skeletal muscle |
| Activation of JNK and IKK by ROS | Bloch-Damti, 2006 [11] | Fao cells (rat hepatoma) |
| IRS threonine phosphorylation | Cedersund, 2008 #227 [12] | Human adipocytes |
| oxidative stress/JNK action on FOXO localisation and transcription | Essers, 2004 [13] | DLD1 (human colon carcinoma); also A14 (3T3 overexpressing InR/ C2C12 (mouse muscle) |
| Circadian variation of physiological insulin | Frayn 1996 [14] | human; entire organism |
| FOXO mediated control of SOD2 | Kops, 2002[15] | DL23 cells (derived from DLD1, human colon carcinoma) |
| FOXO mediated control of InR | Liu, 2007 [16] | rat cardiomyocytes |

The parameters below relate to the FOXO-part of the model , which was developed previously [17] and not refitted here

| process | Ref | experimental system |
| --- | --- | --- |
| Akt-mediated phosphorylation of FOXO | Brunet 1999[18] | CCL39 (Chinese hamster lung) fibroblasts |
| JNK-mediated phosphorylation and transport of FOXO4 | Kops 2002 [15] | DL23 cells (related to DLD-1 human colon carcinoma) |
| IKK-mediated phosphorylation and degradation of FOXO | Hu et al. [19] | 293T and MCF-7 cells |
| Akt-phosphorylation altering transport | Biggs 1999 [20] | in CV1 African green monkey kidney fibroblasts |
| Akt-mediated degradation | Matsuzaki 2003[21]) | HepG2 human liver carcinoma cells |

1. Stagsted J, Hansen T, Roth RA, Goldstein A, Olsson L: **Correlation between insulin receptor occupancy and tyrosine kinase activity at low insulin concentrations and effect of major histocompatibility complex class I-derived peptide**. *J Pharmacol Exp Ther* 1993, **267**(2):997-1001.

2. Mahadev K, Wu X, Zilbering A, Zhu L, Lawrence JT, Goldstein BJ: **Hydrogen peroxide generated during cellular insulin stimulation is integral to activation of the distal insulin signaling cascade in 3T3-L1 adipocytes**. *The Journal of biological chemistry* 2001, **276**(52):48662-48669.

3. Mahadev K, Zilbering A, Zhu L, Goldstein BJ: **Insulin-stimulated hydrogen peroxide reversibly inhibits protein-tyrosine phosphatase 1b in vivo and enhances the early insulin action cascade**. *The Journal of biological chemistry* 2001, **276**(24):21938-21942.

4. Lee SR, Kwon KS, Kim SR, Rhee SG: **Reversible inactivation of protein-tyrosine phosphatase 1B in A431 cells stimulated with epidermal growth factor**. *The Journal of biological chemistry* 1998, **273**(25):15366-15372.

5. Lee SR, Yang KS, Kwon J, Lee C, Jeong W, Rhee SG: **Reversible inactivation of the tumor suppressor PTEN by H2O2**. *The Journal of biological chemistry* 2002, **277**(23):20336-20342.

6. Seo JH, Ahn Y, Lee SR, Yeol Yeo C, Chung Hur K: **The major target of the endogenously generated reactive oxygen species in response to insulin stimulation is phosphatase and tensin homolog and not phosphoinositide-3 kinase (PI-3 kinase) in the PI-3 kinase/Akt pathway**. *Mol Biol Cell* 2005, **16**(1):348-357.

7. Adimora NJ, Jones DP, Kemp ML: **A model of redox kinetics implicates the thiol proteome in cellular hydrogen peroxide responses**. *Antioxid Redox Signal* 2010, **13**(6):731-743.

8. Greene MW, Sakaue H, Wang L, Alessi DR, Roth RA: **Modulation of insulin-stimulated degradation of human insulin receptor substrate-1 by Serine 312 phosphorylation**. *The Journal of biological chemistry* 2003, **278**(10):8199-8211.

9. Ambrogini E, Almeida M, Martin-Millan M, Paik JH, Depinho RA, Han L, Goellner J, Weinstein RS, Jilka RL, O'Brien CA *et al*: **FoxO-mediated defense against oxidative stress in osteoblasts is indispensable for skeletal homeostasis in mice**. *Cell metabolism* 2010, **11**(2):136-146.

10. Archuleta TL, Lemieux AM, Saengsirisuwan V, Teachey MK, Lindborg KA, Kim JS, Henriksen EJ: **Oxidant stress-induced loss of IRS-1 and IRS-2 proteins in rat skeletal muscle: role of p38 MAPK**. *Free Radic Biol Med* 2009, **47**(10):1486-1493.

11. Bloch-Damti A, Potashnik R, Gual P, Le Marchand-Brustel Y, Tanti JF, Rudich A, Bashan N: **Differential effects of IRS1 phosphorylated on Ser307 or Ser632 in the induction of insulin resistance by oxidative stress**. *Diabetologia* 2006, **49**(10):2463-2473.

12. Cedersund G, Roll J, Ulfhielm E, Danielsson A, Tidefelt H, Stralfors P: **Model-based hypothesis testing of key mechanisms in initial phase of insulin signaling**. *PLoS Comput Biol* 2008, **4**(6):e1000096.

13. Essers MA, Weijzen S, de Vries-Smits AM, Saarloos I, de Ruiter ND, Bos JL, Burgering BM: **FOXO transcription factor activation by oxidative stress mediated by the small GTPase Ral and JNK**. *The EMBO journal* 2004, **23**(24):4802-4812.

14. Frayn KN, Humphreys SM, Coppack SW: **Net carbon flux across subcutaneous adipose tissue after a standard meal in normal-weight and insulin-resistant obese subjects**. *Int J Obes Relat Metab Disord* 1996, **20**(9):795-800.

15. Kops GJ, Dansen TB, Polderman PE, Saarloos I, Wirtz KW, Coffer PJ, Huang TT, Bos JL, Medema RH, Burgering BM: **Forkhead transcription factor FOXO3a protects quiescent cells from oxidative stress**. *Nature* 2002, **419**(6904):316-321.

16. Liu TJ, Lai HC, Ting CT, Wang PH: **Bidirectional regulation of upstream IGF-I/insulin receptor signaling and downstream FOXO1 in cardiomyocytes**. *The Journal of endocrinology* 2007, **192**(1):149-158.

17. Smith GR, Shanley DP: **Modelling the Response of FOXO Transcription Factors to Multiple Post-Translational Modifications Made by Ageing-Related Signalling Pathways**. *PloS one* 2010, **5**(6):e11092.

18. Brunet A, Bonni A, Zigmond MJ, Lin MZ, Juo P, Hu LS, Anderson MJ, Arden KC, Blenis J, Greenberg ME: **Akt promotes cell survival by phosphorylating and inhibiting a Forkhead transcription factor**. *Cell* 1999, **96**(6):857-868.

19. Hu MC, Lee DF, Xia W, Golfman LS, Ou-Yang F, Yang JY, Zou Y, Bao S, Hanada N, Saso H *et al*: **IkappaB kinase promotes tumorigenesis through inhibition of forkhead FOXO3a**. *Cell* 2004, **117**(2):225-237.

20. Biggs WH, 3rd, Meisenhelder J, Hunter T, Cavenee WK, Arden KC: **Protein kinase B/Akt-mediated phosphorylation promotes nuclear exclusion of the winged helix transcription factor FKHR1**. *Proceedings of the National Academy of Sciences of the United States of America* 1999, **96**(13):7421-7426.

21. Matsuzaki H, Daitoku H, Hatta M, Tanaka K, Fukamizu A: **Insulin-induced phosphorylation of FKHR (Foxo1) targets to proteasomal degradation**. *Proceedings of the National Academy of Sciences of the United States of America* 2003, **100**(20):11285-11290.
